# Metabolomic profile of genetic liability to type 2 diabetes among 125,000 Mexican adults: a Mendelian randomisation study

**DOI:** 10.2337/dc25-2933

**Published:** 2026-05-01

**Authors:** Fiona Bragg, Jesus Alegre-Diaz, Eirini Trichia, Jason M Torres, Paulina Baca, Adrián Garcilazo-Ávila, Carlos González-Carballo, Raul Ramirez-Reyes, Fernando Rivas, Diego Aguilar-Ramirez, Louisa Gnatiuc Friedrichs, William G Herrington, Michael Hill, Tabitha Hubbard, Alejandra Vergara, Rachel Wade, Rory Collins, Richard Peto, Jaime Berumen, Pablo Kuri-Morales, Roberto Tapia-Conyer, Jonathan R Emberson

**Affiliations:** 1 Clinical Trial Service Unit & Epidemiological Studies Unit, Nuffield Department of Population Health, University of Oxford, Oxford, UK; 2 Health Data Research UK Oxford, University of Oxford, Oxford, UK; 3 Experimental Research Unit from the Faculty of Medicine, National Autonomous University of Mexico, Mexico City, Mexico; 4 Instituto Tecnológico y de Estudios Superiores de Monterrey, Monterrey, Mexico; 5 Faculty of Medicine, National Autonomous University of Mexico, Mexico City, Mexico

## Abstract

**Objective:**

The Mexican population experiences a notably high prevalence of type 2 diabetes (T2D) and high T2D-associated disease risks. We used targeted plasma NMR-metabolomics data within a Mendelian randomisation framework to characterise the metabolomic profile of genetically-predicted liability to T2D in this population.

**Research Design and Methods:**

Between 1998 and 2004, 50,000 men and 100,000 women ≥35 years were recruited from Mexico City. Mendelian randomisation analyses used a genetic risk score (GRS) comprising 1055 established T2D-associated risk variants and eight pathway-specific T2D GRSs constructed from non-overlapping subsets of these variants to estimate associations with 143 metabolic biomarkers (including lipids, lipoproteins, fatty acids, amino acids, ketone bodies and other low molecular weight biomarkers).

**Results:**

Among 125,587 included participants, the T2D GRS explained 6.0% of T2D liability and was not associated with major potential confounders of the relationships of T2D with the circulating metabolome. Genetically-predicted liability to T2D was strongly positively associated with concentrations of VLDL particles and lipids within these, with triglycerides, branch chain amino acids and glycoprotein acetyls, and more modestly positively associated with IDL and LDL, particularly small LDL, particles. Inverse associations were found with relative concentrations of several fatty acids. Pathway-specific T2D GRSs all associated with higher T2D risk but showed differential relationships with circulating metabolic biomarkers.

**Conclusions:**

T2D is associated with widespread changes in the circulating metabolome among adults in Mexico reflecting diverse biological mechanisms and highlighting the importance of effective T2D management, including control of T2D-associated dyslipidaemia, in this population.

## Graphical abstract

**Graphical Abstract:**
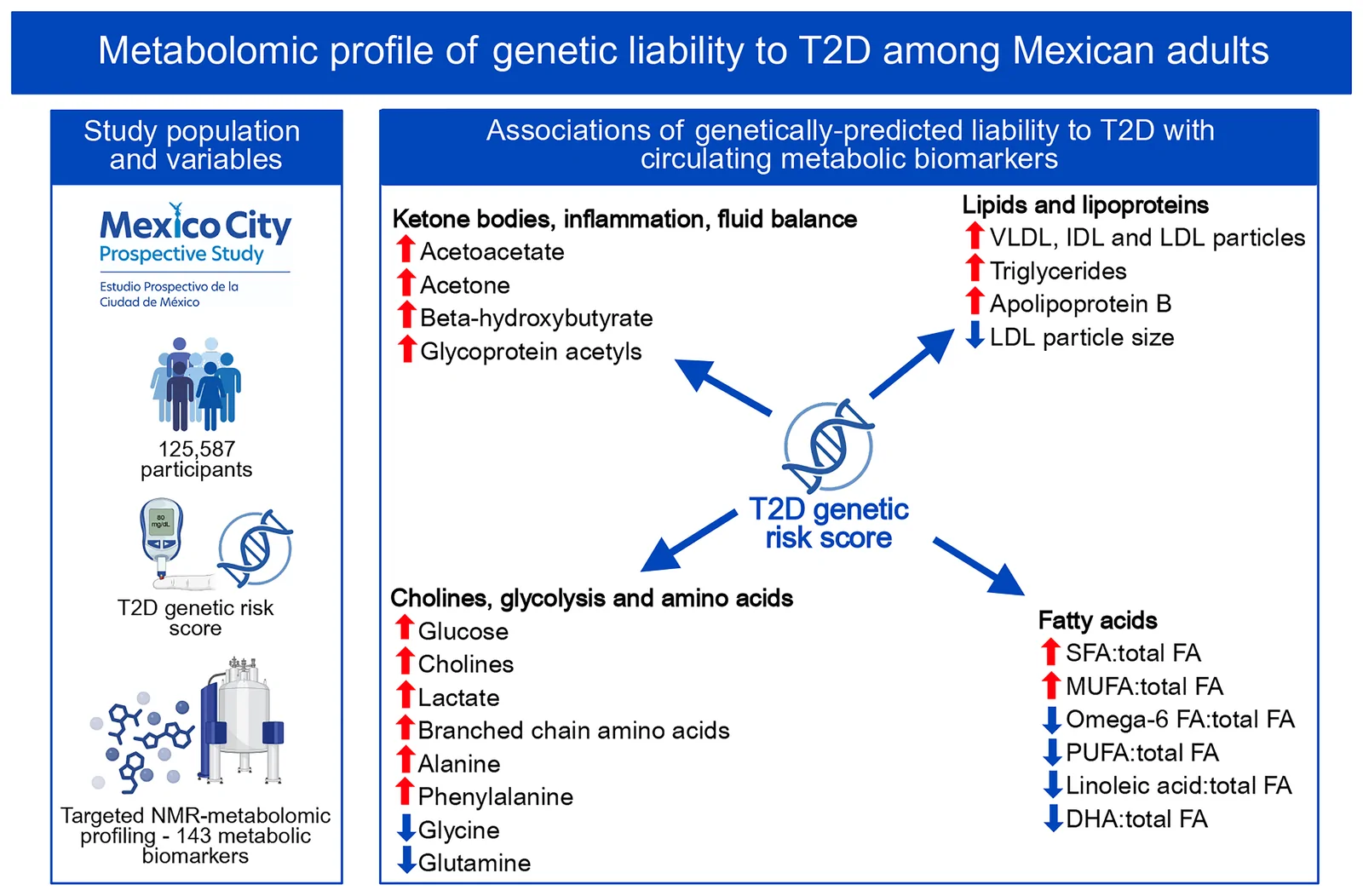

## Introduction

The prevalence of diabetes, most notably type 2 diabetes (T2D), is high and increasing globally (1). This has substantial adverse consequences for health and wellbeing, largely through accompanying risks of various vascular and non-vascular diseases (2–5). The Mexican population experiences a high T2D burden, reflecting both higher T2D prevalence and higher T2D-associated disease risks than in many other populations (1–4,6–8). Factors explaining this high disease burden remain incompletely understood, limiting prediction, prevention and treatment efforts.

The metabolome, reflecting the combined influence of genetic and environmental factors, provides an integrated measure of biologic status and an efficient tool for characterising the molecular basis of disease. Establishing the impact of T2D on the metabolome presents opportunity for improved understanding of the biological basis of the condition and may offer clues to mechanisms underlying its multiple disease associations. Many previous studies investigating the metabolomic profile of T2D have employed observational designs (susceptible to reverse causality, confounding and other important biases) to examine limited metabolic biomarkers among relatively small study populations (9,10). However, advances in high-throughput metabolomic profiling have enabled characterisation of large numbers of biomarkers which, in combination with genetic data, can provide insights into the metabolic consequences of T2D. Using such data, recent Mendelian randomisation (MR) studies based on the UK Biobank population have provided evidence to support causal effects of T2D on various lipids, lipoproteins and amino acids (11–13). However, limited availability of other similar large-scale datasets has restricted independent replication of these findings and constrained investigation of these associations in non-European ancestry populations. Furthermore, previous studies examined only the impact of overall genetic liability to T2D; this limits the extent of our understanding of mechanisms driving observed associations and fails to adequately reflect the marked biological heterogeneity of T2D. Building on these earlier findings, leveraging mechanistic clusters of T2D-associated genetic variants presents opportunities to determine how different pathophysiologic processes underlying T2D impact on the metabolome.

Using data from the Mexico City Prospective Study (MCPS) of approximately 150,000 adults, we report associations of overall and pathway-specific genetically-predicted liability to T2D with approximately 140 circulating metabolic biomarkers quantified using a high-throughput targeted nuclear magnetic resonance (NMR) metabolomics platform.

## Research Design and Methods

A more detailed description of the study design and methods is provided in the Supplementary Text.

### Study design, participants and data collection

Between April 1998 and September 2004, 159,755 adults aged 35 years or older were recruited into the MCPS from two districts (Coyoacán and Iztapalapa) of Mexico City (17). The baseline survey collected detailed information on sociodemographic characteristics, lifestyle factors, medical history and physical measurements. A 10 ml non-fasting venous blood sample was collected, with time since last meal recorded. HbA1c levels were measured in buffy coat samples using a validated high-performance liquid chromatography method (2). Diabetes was ascertained through self-report of a prior doctor diagnosis of diabetes or current diabetes medication use, or an HbA1c level ≥6.5% (48 mmol/mol) (18). Self-reported age at diagnosis and diabetes medication use were used to differentiate between likely type 1 diabetes (T1D) (diagnosed before 35 years and taking insulin at recruitment) and type 2 diabetes (T2D). Metabolomic profiling of baseline plasma samples was undertaken using a targeted NMR metabolomics platform (5).

### Genetic instrument for T2D

Genotyping of study participants used the Illumina Global Screening array v2 (19). Among 1289 single nucleotide polymorphisms (SNPs) associated with T2D in the Type 2 Diabetes Global Genomics Initiative (T2D-GGI) meta-analysis of genome-wide association studies (GWAS) (7), 1055 were available in MCPS. These were used to construct a genetic risk score (GRS) for genetically-predicted liability to T2D as the sum over these SNPs of their effect allele count multiplied by the log odds ratio (OR) for their effect on T2D in the T2D-GGI multi-ancestry meta-regression (20). The T2D-GGI identified 8 non-overlapping subsets of T2D-associated SNPs based on their associations with cardiometabolic traits (20). These were applied to the 1055 SNPs included in the T2D GRS to construct pathway-specific T2D GRSs: beta-cell dysfunction with a positive association with proinsulin (beta-cell +pro-insulin), beta-cell dysfunction with a negative association with proinsulin (beta-cell - pro-insulin), residual glycaemia, body fat, metabolic syndrome, obesity, lipodystrophy, and liver and lipid metabolism.

### Statistical analysis

Genetic analyses were reported in accordance with Strengthening the Reporting of Observational Studies in Epidemiology using Mendelian Randomisation (STROBE-MR) guidelines (21). Nagelkerke’s pseudo R^2^ (22) was used to assess the proportion of T2D variance explained on the liability scale by the T2D GRS. Participant baseline characteristics were assessed across fifths of overall and pathway-specific T2D GRS distributions. MR analyses estimating the associations of genetically-predicted T2D liability with circulating metabolic biomarkers were conducted using the ratio of coefficients method (23). Linear regression was used to assess associations of the T2D GRS with each metabolic biomarker, adjusting for age, sex, the first 7 genetic principal components (PCs) and fasting duration. The derived coefficients were divided by the estimated T2D-GRS association from a logistic regression model relating the T2D GRS to observed T2D (combined previously-diagnosed and undiagnosed), adjusting for age, sex and the first 7 genetic PCs. To improve interpretability, the resulting causal estimates and their SEs were multiplied by 1.10 (i.e. ln 3), corresponding to a trebling in the genetically-predicted odds of T2D (6). Subgroup analyses compared causal effect estimates across strata of age, sex, district and Indigenous American ancestry proportion. Analyses were repeated using the 8 pathway-specific T2D GRSs. To further explore the extent to which associations of genetically-predicted liability to T2D with metabolic biomarkers were due to direct causal associations, where overall or pathway-specific T2D GRSs were found to be associated with potential confounders of the association, multivariable MR (MVMR) was performed (24). Significance testing used the Benjamini-Hochberg method to control the false discovery rate (FDR) at the 5% level (25).

Sensitivity analyses included limiting the study population to participants unrelated to the third family degree and, separately, to participants without prediabetes, and using a Hispanic T2D GRS (20). MR sensitivity analyses employed inverse-variance weighted, MR Pleiotropy Residual Sum and Outlier [MR-PRESSO] (26), weighted median (27), and MR-Egger (28) methods, using associations of individual SNPs with T2D in the T2D-GGI multi-ancestry meta-regression (20) and with metabolic biomarkers in MCPS.

Analyses were conducted using SAS (version 9.4) and R (version 4.3.3).

## Results

### Study population and baseline characteristics

Of the 159,755 participants recruited, 18,923 were excluded from all analyses. These comprised 2959 participants aged 85 years or older at recruitment, a further 14,952 with missing metabolic biomarker data or missing or extreme data on covariates, a further 224 with likely T1D, and a further 788 who reported taking lipid-lowering medication at recruitment. Of the remaining 140,832 participants, 15,245 with missing genotype data or genotype data failing quality control were excluded from genetic analyses, and 6384 with prior diagnosed chronic diseases other than T2D at recruitment were excluded from observational analyses, leaving 125,587 and 134,448 participants for inclusion in the genetic and observational analyses, respectively.

Among 125,587 participants (40,922 men and 84,665 women) in the genetic analysis population, the mean (SD) age was 53 (12) and 67% of participants’ genomic ancestry was, on average, attributable to Indigenous American populations (Table 1). Overall, the mean (SD) BMI was 29.0 (4.8) kg/m^2^ and 18% of participants had T2D at recruitment (13% previously-diagnosed and 5% undiagnosed) but other chronic diseases were uncommon (≤1%). The mean (SD) HbA1c level was 5.5% (0.4%) among participants without T2D and 9.0% (2.4%) among those with previously-diagnosed T2D. Baseline characteristics were similar among the 134,448 participants in the observational analysis population (Supplementary Table S1).

### Overall and pathway-specific T2D GRSs

Per allele effects on T2D of SNPs included in the T2D GRS in the MCPS genetic analysis population were moderately well correlated with those in the T2D-GGI (r= 0.47; Supplementary Figure S1). After accounting for age, sex and the first 7 genetic PCs, the T2D GRS explained an additional 6.0% of variance in T2D and was strongly positively and log-linearly associated with observed T2D overall (OR 1.76 [95% CI 1.74-1.79] per 1 SD higher level) (Supplementary Figure S2) and in major population subgroups (Supplementary Figure S3). The Hispanic T2D GRS was similarly associated with T2D (1.74 [95% CI 1.71-1.76] per 1 SD higher level) (Supplementary Figure S4). There were more modest associations of pathway-specific T2D GRSs with T2D; each 1 SD higher level was associated with 14-33% higher odds, with the exception of the liver and lipid metabolism pathway GRS which was only weakly associated with T2D (OR 1.03 [95% CI 1.02-1.05] per 1 SD higher level) (Supplementary Figure S5) but was based only on 2 SNPs.

The T2D GRS was weakly positively associated with HbA1c level in individuals without diabetes and with Indigenous American ancestry proportion (Supplementary Table S2). More modest directionally consistent trends in HbA1c were observed across fifths of pathway-specific T2D GRSs, most of which also associated positively with Indigenous American ancestry proportion (Supplementary Tables S3-10). The beta-cell +pro-insulin, beta-cell +pro -insulin and lipodystrophy pathway T2D GRSs were inversely associated with BMI, while the obesity pathway T2D GRS was associated positively with BMI and waist circumference. There were no clear differences in other relevant baseline characteristics across fifths of the GRSs.

### Associations of overall T2D GRS with metabolic biomarkers

**Figure 1:**
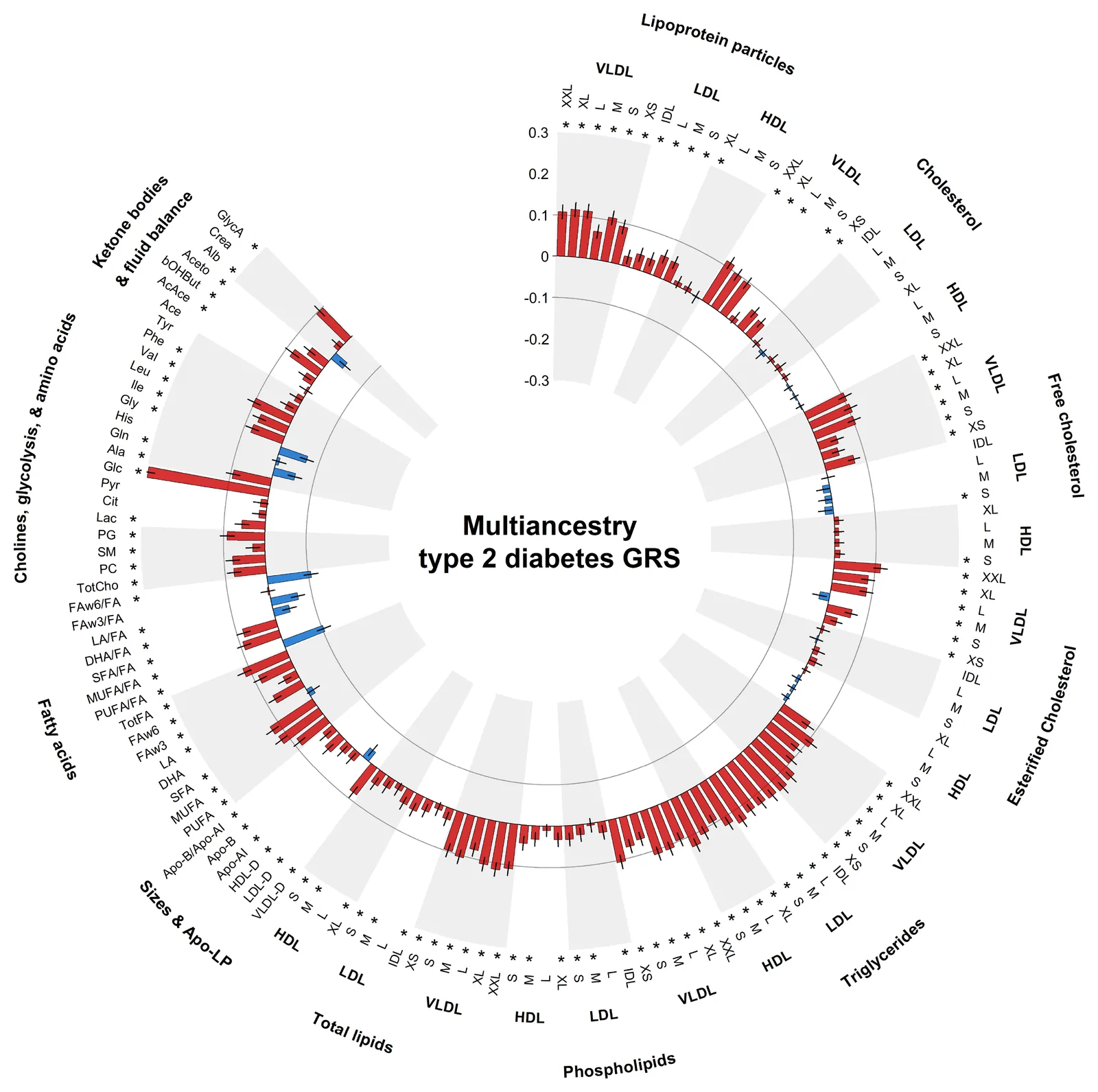
Associations of genetically-predicted liability to type 2 diabetes with circulating metabolic biomarkers among 125,587 participants aged 35-84 years at recruitment Difference (in SD) units of each log-NMR biomarker are per trebling in the genetically-predicted odds of type 2 diabetes and are adjusted for age, sex, the first 7 genetic principal components and fasting time. *False discovery rate controlled p<0.05. Findings based on 86,574 participants for bOHBut, 121,391 participants for citrate, 121,368 participants for creatinine, 120,576 participants for glutamine, 118,255 participants for pyruvate, 121,374 participants for valine. AcAce=acetoacetate; Ace=acetate; Aceto=acetone; Ala=alanine; Alb=albumin; Apo-A1=apolipoprotein A1; Apo-B=apolipoprotein B; bOHBut=beta-hydroxybutyrate; Cit=citrate; Crea=creatinine; DHA=docosahexaenoic acid; FA=fatty acids; FAw3=omega-3; fatty acids; FAw6=omega-6 fatty acids; Glc=glucose; Gln=glutamine; Gly=glycine; Glyc-A=glycoprotein acetyls; HDL=high density lipoproteins; HDL-D=high density lipoprotein particle diameter; His=histidine; IDL=intermediate density lipoproteins; Ile=isoleucine; L=large; LA=linoleic acid; Lac=lactate; LDL=low density lipoproteins; LDL-D=low density lipoprotein particle diameter; Leu=leucine; LP=lipoprotein; M=medium; MUFA=monounsaturated fatty acids; PC=phosphatidylcholines; PG=phosphoglycerides; Phe=phenylalanine; PUFA=polyunsaturated fatty acids; Pyr=pyruvate; S=small; SFA=saturated fatty acids; SM=sphingomyelins; TotFA=total fatty acids; TotCho=total cholines; Tyr=tyrosine; Val=valine; VLDL=very low density lipoproteins; VLDL-D=very low density lipoprotein particle diameter; XL=very large; XS=very small; XXL=extremely large.

The overall T2D GRS was broadly linearly associated (positive, inverse, or flat) with the 143 metabolic biomarkers studied (Supplementary Figure S6). Figure 1 shows the mean difference in SD units of each log-metabolic biomarker per trebling in genetically-predicted odds of T2D (Supplementary Data S1); associations were observed with 105 biomarkers at FDR controlled *p*<0.05 (95 positive and 10 inverse associations). Among the strongest positive associations with higher genetically-predicted T2D liability were marked increases in very low-density lipoprotein (VLDL) particle concentrations (0.07 to 0.12 SD per trebling in genetically-predicted odds of T2D, *p*<0.001) and, with the exception of medium VLDL particles, in cholesterol (0.05 to 0.11 SD, *p*<0.001), triglyceride (0.08 to 0.13 SD, *p*<0.001) and phospholipid (0.06 to 0.11 SD, *p*<0.001) concentrations within these. There were more modest increases in intermediate-density lipoprotein (IDL) (0.02 SD, p=0.01) and low-density lipoprotein (LDL) (0.04 to 0.05 SD, *p*<0.001) particles, and the latter were moderately greater for smaller particles translating to an inverse association of T2D liability with mean LDL particle size (-0.03 SD, *p*=0.003). Reflecting associations with VLDL, IDL and LDL particles, trebling in genetically-predicted odds of T2D was estimated to be associated with a 0.05 SD increase in apolipoprotein-B concentration (*p*<0.001). Genetic liability to T2D was associated only with very large high-density lipoprotein (HDL) particles (0.05 SD per trebling in genetically-predicted odds of T2D, *p*<0.001), and with increased concentrations of phospholipids within very large, medium and small HDL particles (0.03 to 0.04 SD, *p*<0.001). Concentrations of triglycerides across the spectrum of lipoprotein subclasses increased with higher genetically-predicted T2D liability (0.08 to 0.13 SD, *p*<0.001). In contrast, no clear associations were observed with cholesterol concentrations in IDL, LDL or HDL particles.

Absolute fatty acid concentrations increased with higher genetically-predicted liability to T2D (0.03 to 0.12 SD per trebling in genetically-predicted odds of T2D, *p*<0.001) with the exception of docosahexaenoic acid with which there was no clear association. Relative to total fatty acid concentration, there were positive relationships with concentrations of saturated (0.08 SD) and monounsaturated (0.09 SD) fatty acids, but inverse associations with omega-6 (-0.11 SD) and polyunsaturated (-0.10 SD) fatty acids, and with linoleic (-0.07 SD) and docosahexaenoic (-0.04 SD) acids (all *p*<0.001). The strongest association of genetically-predicted T2D liability was with glucose, estimated to increase by 0.30 SD (*p*<0.001) per trebling in genetically-predicted odds of T2D. There were more modest increases in concentrations of cholines (0.03 to 0.09 SD, *p*≤0.001) and various amino acids, including the branched chain amino acids (BCAA) leucine, isoleucine and valine (0.07 to 0.10 SD, *p*<0.001), as well as alanine (0.09 SD, *p*<0.001) and phenylalanine (0.03 SD, *p*=0.003). In contrast, higher genetically-predicted liability to T2D was estimated to decrease glycine (-0.07 SD, *p*<0.001) and glutamine (-0.05 SD, *p*<0.001) levels. Concentrations of lactate (0.06 SD, *p*<0.001) and of ketone bodies acetoacetate (0.03 SD, *p*=0.006), acetone (0.05 SD, *p*<0.001) and beta-hydroxybutyrate (0.08 SD, *p*<0.001) increased with greater T2D liability, as did glycoprotein acetyl levels (0.10 SD, *p*<0.001).

The described associations of genetically-predicted T2D liability with metabolic biomarkers were largely consistent in direction and statistical significance with relationships of T2D in observational analyses (Supplementary Figure S7, S8).

For most metabolic biomarkers, the direction of association and apparent causal relevance of genetic liability to T2D were robust across MR sensitivity analyses based on summary data (Supplementary Data S2). Associations with VLDL particles and lipids within them, BCAA and glycoprotein acetyls were attenuated but largely remained statistically significant in the MR-Egger model, but with evidence of directional pleiotropy for larger VLDL particles, BCAA and glycoprotein acetyls (Egger intercept *p*-values ≤0.02). There was also evidence of directional pleiotropy in associations with total fatty acids and with relative concentrations of many individual fatty acids (Egger intercept p-values <0.02), for which the causal estimate from MR-Egger was compatible with the null. In contrast, for cholesterol concentrations in large and medium HDL particles, evidence of horizontal pleiotropy (Egger intercept p-values ≤0.05) was accompanied by estimated increases in these metabolic biomarkers with higher genetically-predicted T2D liability (p<0.05).

### Associations of pathway-specific T2D GRSs with metabolic biomarkers

**Figure 2:**
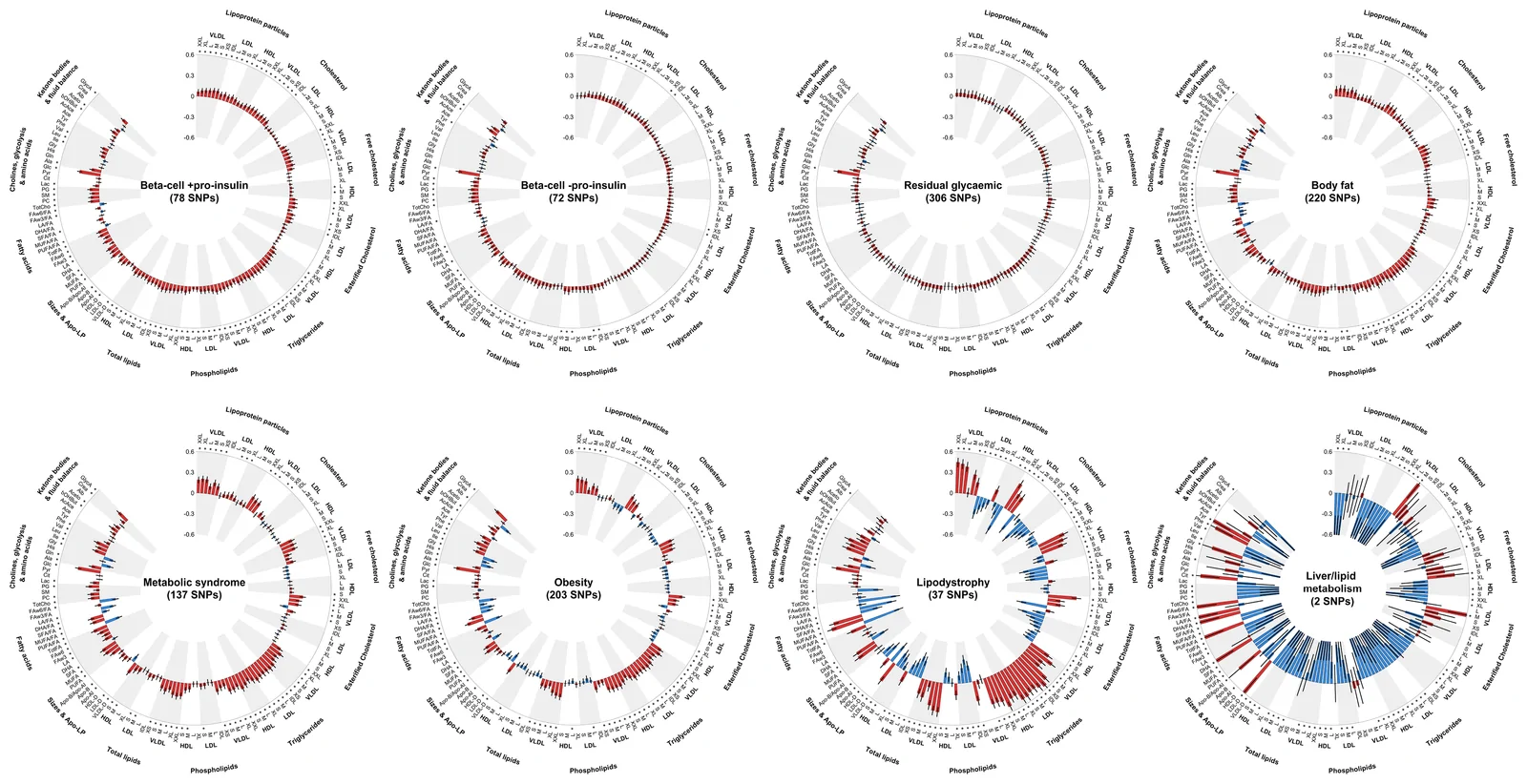
Associations of pathway-specific type 2 diabetes GRSs with circulating metabolic biomarkers among 125,587 participants aged 35-84 years at recruitment Difference (in SD) units of each log-NMR biomarker are per trebling in the genetically-predicted odds of type 2 diabetes and are adjusted for age, sex, the first 7 genetic principal components and fasting time. *False discovery rate controlled p<0.05. Findings based on 86,574 participants for bOHBut, 121,391 participants for citrate, 121,368 participants for creatinine, 120,576 participants for glutamine, 118,255 participants for pyruvate, 121,374 participants for valine. AcAce=acetoacetate; Ace=acetate; Aceto=acetone; Ala=alanine; Alb=albumin; Apo-A1=apolipoprotein A1; Apo-B=apolipoprotein B; bOHBut=beta-hydroxybutyrate; Cit=citrate; Crea=creatinine; DHA=docosahexaenoic acid; FA=fatty acids; FAw3=omega-3; fatty acids; FAw6=omega-6 fatty acids; Glc=glucose; Gln=glutamine; Gly=glycine; Glyc-A=glycoprotein acetyls; GRS=genetic risk score; HDL=high density lipoproteins; HDL-D=high density lipoprotein particle diameter; His=histidine; IDL=intermediate density lipoproteins; Ile=isoleucine; L=large; LA=linoleic acid; Lac=lactate; LDL=low density lipoproteins; LDL-D=low density lipoprotein particle diameter; Leu=leucine; LP=lipoprotein; M=medium; MUFA=monounsaturated fatty acids; PC=phosphatidylcholines; PG=phosphoglycerides; Phe=phenylalanine; PUFA=polyunsaturated fatty acids; Pyr=pyruvate; S=small; SFA=saturated fatty acids; SM=sphingomyelins; TotFA=total fatty acids; TotCho=total cholines; Tyr=tyrosine; Val=valine; VLDL=very low density lipoproteins; VLDL-D=very low density lipoprotein particle diameter; XL=very large; XS=very small; XXL=extremely large.

Figure 2 shows the effects of the pathway-specific T2D GRSs on circulating metabolic biomarker concentrations (Supplementary Data S1). Associations of the liver/lipid metabolism pathway GRS, including 2 SNPs and weakly associated with T2D, should be interpreted with caution. The remaining 7 pathway-specific T2D GRSs were strongly positively associated with plasma glucose concentration, which was the only biomarker influenced by the residual glycaemia pathway GRS. Increases in the beta-cell +pro-insulin, body fat, metabolic syndrome, obesity and lipodystrophy pathway T2D GRSs were associated with increased levels of VLDL particles and lipids within them and of triglycerides across lipoprotein subclasses. These associations were generally strongest for the lipodystrophy pathway T2D GRS. The beta-cell -pro-insulin pathway T2D GRS was largely unrelated to these biomarkers, but increases in both beta-cell pathway GRSs were reasonably consistently associated with higher concentrations of IDL and LDL particles and of cholesterol and phospholipid concentrations in the former. This was in clear contrast with strong inverse associations of the lipodystrophy pathway T2D GRS with these biomarkers and with cholesterol and phospholipid concentrations in LDL particles. Similarly, increases in the beta-cell pathway T2D GRSs were associated with increased HDL particle and apolipoprotein-A1 concentrations, while the obesity and lipodystrophy pathway T2D GRSs were largely inversely associated with these measures and with cholesterol, specifically esterified cholesterol, in these particles. Most pathway-specific T2D GRSs were positively associated with BCAAs, but associated variably with other amino acids. Increases in metabolic syndrome and lipodystrophy pathway T2D GRSs only were associated with increased creatinine levels. Otherwise, most associations of the 6 pathway-specific T2D GRSs were broadly qualitatively consistent with those of the overall T2D GRS.

In MVMR analyses, after adjusting beta-cell and lipodystrophy pathway T2D GRS estimates for BMI (using a previously described BMI GRS (29)), the associations with circulating metabolic biomarkers persisted largely unchanged (Supplementary Figure S9, Supplementary Data S3). In contrast, when obesity pathway T2D GRS estimates were adjusted for BMI and WC (29), associations with 21 metabolic biomarkers (including cholesterol concentrations in HDL particles, non-BCAAs and relative concentrations of some fatty acids) were no longer statistically significant and others were more modestly attenuated. In addition, the direction of associations with 13 biomarkers (including concentrations of HDL particles, apolipoprotein-A1 and of cholesterol in IDL particles) were reversed, and 33 associations not evidenced previously (including increases in concentrations of LDL particles, apolipoprotein-B, cholesterol in smaller VLDL particles and cholines with higher T2D liability) were observed.

### Sensitivity analyses

The associations with metabolic biomarkers of overall genetically-predicted T2D liability and of genetic liability to T2D mechanistic subtypes differed little across population subgroups defined by age, sex, district of residence or Indigenous American ancestry proportion (Supplementary Figures S10-S14). In sensitivity analyses, associations did not differ appreciably when analyses were repeated using the Hispanic T2D GRS (Supplementary Figure S15) or, separately, among participants unrelated up to the third family degree (Supplementary Figure S16, S17) or after limiting the study population to participants without prediabetes (Supplementary Figure S18).

## Conclusions

Combining genetic and targeted plasma metabolomics data for over 125,000 adults from Mexico City, this study provides detailed characterisation of the metabolomic profile of liability to T2D in a non-European population at high risk of complications of T2D. Greater genetically-predicted T2D liability was associated with widespread changes in the circulating metabolome, including marked increases in concentrations of VLDL particles and lipids within them, triglycerides, amino acids, including BCAAs, and glycoprotein acetyls. Varied associations of pathway-specific T2D GRSs with metabolic biomarkers provide insights into likely mechanisms underlying these effects.

The metabolomic platform used in the present study enabled detailed exploration of the effects of T2D liability on lipids and lipoproteins. This revealed marked increases in concentrations of VLDL particles, lipids within these and triglycerides across all lipoprotein subclasses with higher T2D liability, and more modest increases in IDL and LDL, particularly small LDL, particles. These are characteristic features of insulin resistance (30), and the presented findings, including stronger associations of insulin resistance related pathway-specific T2D GRSs (i.e. obesity, lipodystrophy and metabolic syndrome) with many of these metabolic biomarkers, support these as consequences of T2D development. Qualitatively consistent associations with VLDL particles and triglycerides have been reported in European ancestry two-sample MR studies (11–13), although these may be weaker than in the MCPS population (12). Moreover, these previous studies described decreased concentrations of IDL, LDL and HDL particles and cholesterol within these with greater genetically-predicted liability to T2D (11–13), contrasting with findings from the present study in which there was no clear association of the overall T2D GRS with HDL particles or lipids within them. These differences could reflect population differences in the typical pathophysiology of T2D, and pleiotropic effects of some T2D-associated genetic variants (eg, on adiposity) might contribute. Differences in frequency of use of lipid-lowering medications, which were uncommon in MCPS participants (<0.6% at recruitment), may also be relevant. Previous MR analyses in a population with high prevalence of statin use showed that this can distort associations of genetically-predicted liability to T2D with the circulating metabolome (12). Using age as a proxy for statin use, this previous study observed differences across age groups in the associations of genetically-predicted T2D liability with certain lipids and lipoproteins (eg, triglycerides and VLDL, IDL, LDL and HDL measures); associations of genetically-predicted T2D liability in the MCPS population align most closely with those in the youngest age group (with lowest statin use) (12). The associations of T2D liability with increased concentrations of known atherogenic apolipoprotein-B containing lipoprotein particles and triglycerides (31–33) may contribute to increased risks of atherosclerotic cardiovascular diseases in T2D. Moreover, the notably strong effects of T2D liability on these lipid and lipoprotein measures in the MCPS population might contribute to reported stronger causal relationships of T2D with CVD mortality in this population (6), highlighting the importance of effective control of T2D-related dyslipidaemia.

The increase in BCAA concentrations with greater genetically-predicted T2D liability in MCPS is consistent (11–13,34) with findings from European ancestry studies. We were able to build on these earlier findings, to demonstrate that this predominantly reflected the associations of obesity, metabolic syndrome and lipodystrophy pathway T2D GRSs, supporting the assertion that higher BCAA concentrations result from insulin resistance (35). The apparent effects of genetically-predicted T2D on other amino acids in MCPS were similarly largely comparable with previous findings (11–13,34), including increases in alanine and phenylalanine and lowering of glycine concentrations. Likewise, the associations with glycolysis related biomarkers, relative fatty acid concentrations and glycoprotein acetyls (a marker of low-grade inflammation) in the present study provide valuable replication of associations reported previously in European populations (11–13,34). Conversely, increased concentrations of several choline biomarkers and of ketone bodies with increasing genetically-predicted liability to T2D in MCPS contrast with reductions (11–13) and null associations (11,12), respectively, with these biomarkers in European ancestry population studies. The reasons for these differences remain unclear, highlighting the need for further investigation of the metabolomic profile of genetic liability to T2D among diverse populations.

T2D is a highly heterogeneous condition resulting from dysfunction across multiple molecular pathways. While these share a dysglycaemic effect, the presented associations of pathway-specific T2D GRSs demonstrate the extent to which pathophysiological processes underlying T2D differ in their contributions to changes in the metabolome. Findings from multiple studies (20,36) including MCPS (6) have shown varied disease associations of these pathway-specific T2D GRSs. These include consistently observed stronger associations of the obesity pathway T2D GRS with vascular disease risks (6,20). This might reflect the strong associations of this pathway-specific T2D GRS with atherogenic lipids and lipoproteins, and suggests obesity partially explains the effects of genetic liability to T2D on the metabolome. These findings highlight the potential value of these pathway-specific T2D GRSs in advancing understanding of mechanisms through which T2D has its effects, including on downstream disease risks, as well as informing patient stratification for risk, enabling improved disease prediction, prevention and management.

Use of large-scale metabolomic profiling and genotyping data in a previously understudied population are major strengths of the present analyses, facilitating detailed and reliable characterisation of the impact of genetically-predicted T2D liability on the circulating metabolome among Mexican adults. The use of an established targeted metabolomics platform facilitated comparison with findings of previous studies (11–13,34), providing insights into potential mechanisms underlying the higher T2D-associated disease risks in Mexico (4,6). Moreover, investigation of the relevance of mechanistic clusters of T2D-associated genetic variants contrasts with previous studies’ focus only on overall genetic liability to T2D. The infrequent use of lipid-lowering medications in the MCPS population facilitated exclusion from analyses of participants taking such medications without consequent risks of collider bias (37), reducing potential treatment-associated biases and confounding. However, the study has limitations. The paucity of Mexican population datasets including relevant data limited independent replication of the findings. Moreover, the focus on a Mexican population may limit generalisability of the findings, although comparable associations of multi-ancestry and Hispanic ancestry T2D GRSs suggests their wider applicability. The emphasis of the metabolomics platform used on lipid and lipoprotein measures limited the breadth of investigation of the metabolic effects of T2D. In addition, metabolic biomarkers were quantified in non-fasting blood samples. However, fasting duration has been found to account for only a small proportion of variability in concentrations of these biomarkers (38), which would be further reduced by adjustment for fasting duration in the present analyses. Differentiation between T1D and T2D was based on self-reported age at diabetes diagnosis and medication use, with potential for misclassification. Although this may have resulted in exclusion of some participants with young-onset T2D, the impact of this on the findings presented would be expected to be minimal given fewer than 1% (n=224) of participants with diabetes were excluded due to likely T1D. Furthermore, the impact of liability to T2D was examined; this may have effects on the metabolome independent of T2D status (39), necessitating caution in inferring the effects of T2D itself based on the presented findings. The optimal approach for defining mechanistic clusters of T2D-associated genetic variants remains uncertain (40), and the pathway-specific T2D GRSs studied (20) may not fully reflect T2D pathogenic pathways.

Genetically-predicted T2D liability is associated with diverse changes in the circulating metabolome, reflecting differential associations of distinct genetically-determined T2D biological pathways. The findings presented highlight specific features of the metabolomic profile of genetically-predicted liability to T2D in the Mexican population. These include strong adverse effects of liability to T2D on apolipoprotein-B containing lipoprotein particles and triglycerides, but also increases in choline biomarkers and ketone bodies not previously observed in European population studies. The findings usefully advance our understanding of possible mechanisms underlying high T2D-associated risks of both vascular and non-vascular diseases in this population and demonstrate the importance of effective T2D management, including through optimised secondary prevention such as use of lipid-lowering therapies.

## Supplementary Material

Graphical Abstract

Online Supplementary Material

STROBE-MR Checklist

Supplementary Data

## Figures and Tables

**Table 1:** Baseline characteristics of 125,587 participants aged 35-84 years at recruitment

	Men	Women	Total
No. of participants	40922	84665	125587
**Age, ancestry and socioeconomic factors**			
Age, years	54 (13)	52 (12)	53 (12)
Indigenous American ancestry, %	67	67	67
Resident of Coyoacan	16982 (41)	31237 (37)	48219 (38)
Resident of Iztapalapa	23940 (59)	53428 (63)	77368 (62)
University or high school educated	9523 (23)	9499 (11)	19022 (15)
**Lifestyle factors**			
Current smoker	17958 (44)	16836 (20)	34794 (28)
Current alcohol drinker	31150 (76)	52301 (62)	83451 (66)
Physical activity 1+ times/week	12208 (30)	15677 (19)	27885 (22)
**Physical measurements, HbA1c, eGFR and fasting duration**			
Height, cm	165 (7)	151 (6)	156 (9)
Weight, kg	75.8 (12.7)	67.8 (12.5)	70.4 (13.1)
BMI, kg/m^2^	27.9 (4.1)	29.6 (5.1)	29.0 (4.8)
Waist circumference, cm	96 (10)	93 (12)	94 (11)
Waist-to-hip ratio	0.95 (0.06)	0.88 (0.06)	0.90 (0.07)
Blood pressure, mmHg			
Systolic	129 (16)	127 (17)	127 (17)
Diastolic	84 (10)	82 (10)	83 (10)
HbA1c, %*	5.5 (0.4)	5.5 (0.4)	5.5 (0.4)
eGFR, mL/min/1.73 m^2†^	97 (16)	98 (16)	97 (16)
Fasting duration, hours	4.1 (4.4)	3.9 (4.2)	3.9 (4.3)
**Medical history**			
Type 2 diabetes			
Previously-diagnosed	5412 (13)	11193 (13)	16605 (13)
Undiagnosed	2131 (5)	4214 (5)	6345 (5)
Total	7543 (18)	15407 (18)	22950 (18)
Ischaemic heart disease	784 (2)	988 (1)	1772 (1)
Stroke	402 (1)	829 (1)	1231 (1)
Chronic kidney disease	261 (1)	653 (1)	914 (1)
Cirrhosis	115 (<0.5)	63 (<0.5)	178 (<0.5)
Cancer	246 (1)	1227 (1)	1473 (1)
Emphysema	177 (<0.5)	175 (<0.5)	352 (<0.5)
**Long-term medication use**			
Any diabetes medication	4193 (10)	9019 (11)	13212 (11)
Any anti-hypertensive medication	4690 (11)	14795 (17)	19485 (16)
Any anti-thrombotic medication	1181 (3)	2505 (3)	3686 (3)

Mean (SD) or n (%)

* Among participants without diabetes; to convert % to mmol/mol, use the formula: HbA1c (mmol/mol) = 10.929 x (HbA1c [%] −2.15)

† Estimated using the Chronic Kidney Disease Epidemiology Collaboration 2009 creatinine equation (PMID: 34554658)

BMI=body mass index; eGFR=estimated glomerular filtration rate

## Data Availability

Data from the Mexico City Prospective Study are available to bona fide academic researchers. For more details, the study’s Data and Sample Sharing policy may be viewed (in English or Spanish) at https://www.ctsu.ox.ac.uk/research/mcps. Available study data can be examined in detail through the study’s Data Showcase, available at https://datashare.ndph.ox.ac.uk/mexico/. MCPS ancestry-specific allele frequencies are available in a public browser (https://rgc-mcps.regeneron.com/).

## Twitter summary

Genetically-predicted liability to T2D is associated with widespread changes in the circulating metabolome in Mexican adults possibly driving high T2D-associated vascular risk in this population

## Article Highlights

- **Why did we undertake this study?**
  To improve understanding of the metabolomic profile of type 2 diabetes (T2D) among Mexican adults.
- **What is the specific question(s) we wanted to answer?**
  How is genetically-predicted T2D liability, overall and stratified by mechanistic clusters, associated with circulating metabolic biomarkers in Mexican adults?
- **What did we find?**
  Genetically-predicted T2D liability was associated with widespread changes in the metabolome, including marked changes in concentrations of lipids, lipoproteins, amino acids, fatty acids and inflammatory markers. These associations differed across different pathway-specific T2D genetic risk scores.
- **What are the implications of our findings?**
  The observed associations of genetically-predicted T2D liability highlight distinct features of the metabolomic profile of T2D and its mechanistic subtypes among Mexican adults, providing clues about possible mechanisms underlying T2D-associated disease risks.
