# Supplementary material for: Metabolomic profile of genetic liability to type 2 diabetes among 125,000 Mexican adults: a Mendelian randomisation study
Source: Diabetes Care. Author manuscript; Available in PMC 2026 Nov 2. (PMC7619673; doi:10.2337/dc25-2933)
Supplement: Supplementary file 1 [file EMS212945-supplement-Graphical_Abstract.pdf]

Metabolomic profile of genetic liability to T2D among Mexican adults

Study population and variables

Mexico City Prospective Study

Estudio Prospectivo de la Ciudad de México

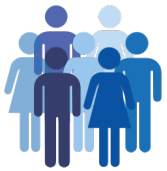

125,587 participants

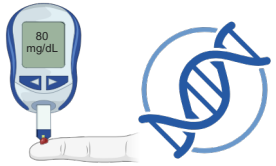

T2D genetic risk score

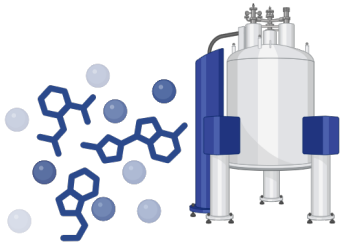

Targeted NMR-metabolomic profiling - 143 metabolic biomarkers

Associations of genetically-predicted liability to T2D with circulating metabolic biomarkers

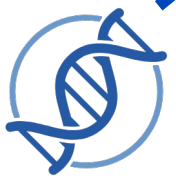

T2D genetic risk score

Ketone bodies, inflammation, fluid balance

- ↑ Acetoacetate
- ↑ Acetone
- ↑ Beta-hydroxybutyrate
- ↑ Glycoprotein acetyls

Lipids and lipoproteins

- ↑ VLDL, IDL and LDL particles
- ↑ Triglycerides
- ↑ Apolipoprotein B
- ↓ LDL particle size

Cholines, glycolysis and amino acids

- ↑ Glucose
- ↑ Cholines
- ↑ Lactate
- ↑ Branched chain amino acids
- ↑ Alanine
- ↑ Phenylalanine
- ↓ Glycine
- ↓ Glutamine

Fatty acids

- ↑ SFA:total FA
- ↑ MUFA:total FA
- ↓ Omega-6 FA:total FA
- ↓ PUFA:total FA
- ↓ Linoleic acid:total FA
- ↓ DHA:total FA
