# Supplementary material for: Metabolomic profile of genetic liability to type 2 diabetes among 125,000 Mexican adults: a Mendelian randomisation study
Source: Diabetes Care. Author manuscript; Available in PMC 2026 Nov 2. (PMC7619673; doi:10.2337/dc25-2933)
Supplement: Supplementary file 2 [file EMS212945-supplement-Online_Supplementary_Material.pdf]

Metabolomic profile of genetic liability to type 2 diabetes (T2D) among 125,000 Mexican adults: a Mendelian randomisation study

Supplementary Material, Table of Contents

Supplementary tables	Page
1. Baseline characteristics of 134,448 participants (observational analysis population) aged 35-84 years at recruitment	3
Baseline characteristics of 125,587 participants aged 35-84 years at recruitment by fifths of:	
2. Multi-ancestry T2D GRS	4
3. Beta-cell +pro-insulin pathway T2D GRS	5
4. Beta-cell -pro-insulin pathway T2D GRS	6
5. Residual glycaemic pathway T2D GRS	7
6. Body fat pathway T2D GRS	8
7. Metabolic syndrome pathway T2D GRS	9
8. Obesity pathway T2D GRS	10
9. Lipodystrophy pathway T2D GRS	11
10. Liver and lipid metabolism pathway T2D GRS	12

Supplementary figures	
1. Genetic variant effect size estimates for T2D in Mexico City Prospective Study and in T2D Global Genomics Initiative multi-ancestry GWAS	13
2. Association of T2D GRS with observed T2D status among 125,587 participants aged 35-84 years at recruitment	14
3. Association of T2D GRS with observed T2D status by sex, age and Indigenous American ancestry proportion among 125,587 participants aged 35-84 years at recruitment	15
4. Association of Hispanic T2D GRS with observed T2D status among 125,587 participants aged 35-84 years at recruitment	16
5. Association of pathway-specific T2D GRSs with observed T2D status among 125,587 participants aged 35-84 years at recruitment	17
6. Associations of T2D GRS with circulating metabolic biomarkers among 125,587 participants aged 35-84 years at recruitment	18
7. Associations of observed T2D status at recruitment with circulating metabolic biomarkers among 134,448 participants aged 35-84 years at recruitment	25
8. Associations of observed previously-diagnosed and undiagnosed T2D status at recruitment with circulating metabolic biomarkers among 134,448 participants aged 35-84 years at recruitment	26
9. Associations of pathway-specific T2D GRSs with circulating metabolic biomarkers among 125,587 participants aged 35-84 years at recruitment, before and after adjustment for genetically-predicted measures of adiposity	27
10. Associations of genetically-predicted liability to T2D with circulating metabolic biomarkers among 125,587 participants aged 35-84 years at recruitment, by age, sex, district and Indigenous American ancestry proportion	28
Associations of pathway-specific T2D GRSs with circulating metabolic biomarkers among 125,587 participants aged 35-84 years at recruitment	
11. By age	29
12. By sex	30
13. By district	31
14. By Indigenous American ancestry proportion	32

15. Associations of Hispanic T2D GRS with circulating metabolic biomarkers among 125,587 participants aged 35-84 years at recruitment 33
16. Associations of genetically-predicted liability to T2D with circulating metabolic biomarkers among participants aged 35-84 years at recruitment, by relatedness 34
17. Associations of pathway-specific T2D GRSs with circulating metabolic biomarkers among participants aged 35-84 years at recruitment, by relatedness 35
18. Associations of genetically-predicted liability to type 2 diabetes with circulating metabolic biomarkers among 97,542 participants aged 35-84 years and without prediabetes at recruitment 36

Supplementary text 37

Supplementary Table S1: Baseline characteristics of 134,448 participants (observational analysis population) aged 35-84 years at recruitment

	Men	Women	Total
No. of participants	43899	90549	134448
**Age and socioeconomic factors**			
Age, years	53 (12)	52 (12)	52 (12)
Resident of Coyoacán	18732 (43)	34119 (38)	52851 (39)
Resident of Iztapalapa	25167 (57)	56430 (62)	81597 (61)
University or high school educated	10250 (23)	10252 (11)	20502 (15)
**Lifestyle factors**			
Current smoker	19571 (45)	18093 (20)	37664 (28)
Current alcohol drinker	33758 (77)	56322 (62)	90080 (67)
Physical activity 1+ times/week	12987 (30)	16649 (18)	29636 (22)
**Physical measurements, HbA1c, eGFR and fasting duration**			
Height, cm	165 (7)	151 (6)	156 (9)
Weight, kg	75.8 (12.7)	67.8 (12.4)	70.4 (13.0)
BMI, kg/m^2	27.9 (4.1)	29.6 (5.1)	29.0 (4.8)
Waist circumference, cm	96 (10)	93 (12)	94 (11)
Waist-to-hip ratio	0.95 (0.06)	0.88 (0.06)	0.90 (0.07)
Blood pressure, mmHg			
Systolic	128 (16)	127 (17)	127 (17)
Diastolic	84 (10)	82 (10)	83 (10)
HbA1c, %*	5.5 (0.4)	5.5 (0.4)	5.5 (0.4)
eGFR, mL/min/1.73 m^2†	97 (15)	98 (16)	98 (16)
Fasting duration, hours	4.1 (4.4)	3.9 (4.2)	3.9 (4.3)
**Medical history**			
Type 2 diabetes			
Previously diagnosed	5559 (13)	11603 (13)	17162 (13)
Undiagnosed	2260 (5)	4464 (5)	6724 (5)
Total	7819 (18)	16067 (18)	23886 (18)
**Long-term medication use**			
Any diabetes medication	4304 (10)	9362 (10)	13666 (10)
Any anti-hypertensive medication	4374 (10)	14913 (16)	19287 (14)
Any anti-thrombotic medication	952 (2)	2355 (3)	3307 (2)

Mean (SD) or n (%)
*Among participants without diabetes; to convert % to mmol/mol, use the formula: HbA1c (mmol/mol) = 10.929 x (HbA1c [%] −2.15)
†Estimated using the Chronic Kidney Disease Epidemiology Collaboration 2009 creatinine equation (PMID: 34554658)
BMI=body mass index; eGFR=estimated glomerular filtration rate

Supplementary Table S2: Baseline characteristics of 125,587 participants aged 35-84 years at recruitment by fifths of multi-ancestry type 2 diabetes GRS

	Fifth of type 2 diabetes GRS					Overall
	I	II	III	IV	V	
No. of participants	25117	25118	25117	25118	25117	125587
**Age, sex, ancestry and socioeconomic factors**						
Age, years	53 (13)	53 (13)	53 (12)	53 (12)	52 (12)	53 (12)
Men	8244 (33)	8189 (33)	8219 (33)	8131 (32)	8139 (32)	40922 (33)
Indigenous American ancestry, %	63	67	68	69	69	67
Resident of Coyoacán	10030 (40)	9653 (38)	9554 (38)	9585 (38)	9397 (37)	48219 (38)
Resident of Iztapalapa	15087 (60)	15465 (62)	15563 (62)	15533 (62)	15720 (63)	77368 (62)
University or high school educated	4220 (17)	3816 (15)	3786 (15)	3674 (15)	3526 (14)	19022 (15)
**Lifestyle factors**						
Current smoker	6966 (28)	6845 (27)	6991 (28)	6939 (28)	7053 (28)	34794 (28)
Current alcohol drinker	16957 (68)	16851 (67)	16810 (67)	16489 (66)	16344 (65)	83451 (66)
Physical activity 1+ times/week	5950 (24)	5570 (22)	5541 (22)	5405 (22)	5419 (22)	27885 (22)
**Physical measurements, HbA1c, eGFR and fasting duration**						
Height, cm	156 (9)	156 (9)	156 (9)	155 (9)	155 (9)	156 (9)
Weight, kg	71.2 (13.5)	70.8 (13.2)	70.3 (13.0)	70.3 (12.9)	69.5 (12.7)	70.4 (13.1)
BMI, kg/m^2	29.2 (5.0)	29.2 (4.9)	29.0 (4.8)	29.1 (4.8)	28.7 (4.7)	29.0 (4.8)
Waist circumference, cm	95 (12)	95 (11)	94 (11)	94 (11)	94 (11)	94 (11)
Waist-to-hip ratio	0.90 (0.07)	0.90 (0.07)	0.90 (0.07)	0.91 (0.07)	0.90 (0.07)	0.90 (0.07)
Blood pressure, mmHg						
Systolic	127 (17)	128 (17)	127 (16)	128 (17)	127 (17)	127 (17)
Diastolic	83 (10)	83 (10)	83 (10)	83 (10)	83 (10)	83 (10)
HbA1c, %*	5.5 (0.7)	5.6 (0.8)	5.6 (0.9)	5.7 (1.0)	5.8 (1.2)	5.6 (1.0)
eGFR, mL/min/1.73 m^2†	97 (16)	97 (16)	98 (16)	98 (16)	98 (17)	97 (16)
Fasting duration, hours	3.9 (4.3)	3.9 (4.3)	3.9 (4.3)	3.9 (4.3)	3.9 (4.3)	3.9 (4.3)
**Medical history**						
Type 2 diabetes						
Previously diagnosed	1528 (6)	2447 (10)	3177 (13)	3871 (15)	5582 (22)	16605 (13)
Undiagnosed	694 (3)	1018 (4)	1251 (5)	1483 (6)	1899 (8)	6345 (5)
Total	2222 (9)	3465 (14)	4428 (18)	5354 (21)	7481 (30)	22950 (18)
Ischaemic heart disease	362 (1)	369 (1)	332 (1)	315 (1)	394 (2)	1772 (1)
Stroke	234 (1)	249 (1)	237 (1)	267 (1)	244 (1)	1231 (1)
Chronic kidney disease	191 (1)	171 (1)	179 (1)	169 (1)	204 (1)	914 (1)
Cirrhosis	36 (<0.5)	38 (<0.5)	28 (<0.5)	37 (<0.5)	39 (<0.5)	178 (<0.5)
Cancer	318 (1)	315 (1)	275 (1)	297 (1)	268 (1)	1473 (1)
Emphysema	94 (<0.5)	79 (<0.5)	66 (<0.5)	55 (<0.5)	58 (<0.5)	352 (<0.5)
**Long-term medication use**						
Any diabetes medication	1185 (5)	1904 (8)	2496 (10)	3131 (12)	4496 (18)	13212 (11)
Any anti-hypertensive medication	3793 (15)	4062 (16)	3834 (15)	3930 (16)	3866 (15)	19485 (16)
Any anti-thrombotic medication	797 (3)	803 (3)	731 (3)	667 (3)	688 (3)	3686 (3)

Mean (SD) or n (%)

*Among participants without diabetes; to convert % to mmol/mol, use the formula: HbA1c (mmol/mol) = 10.929 x (HbA1c [%] −2.15)

†Estimated using the Chronic Kidney Disease Epidemiology Collaboration 2009 creatinine equation (PMID: 34554658)

BMI=body mass index; eGFR=estimated glomerular filtration rate; GRS=genetic risk score

Supplementary Table S3: Baseline characteristics of 125,587 participants aged 35-84 years at recruitment by fifths of beta-cell +pro-insulin pathway type 2 diabetes GRS (78 SNPs)

	Fifth of type 2 diabetes GRS					Overall
	**I**	**II**	**III**	**IV**	**V**	
No. of participants	25117	25118	25117	25118	25117	125587
**Age, sex, ancestry and socioeconomic factors**						
Age, years	53 (12)	53 (12)	53 (12)	53 (12)	53 (12)	53 (12)
Men	8172 (33)	8265 (33)	8125 (32)	8176 (33)	8184 (33)	40922 (33)
Indigenous American ancestry, %	71	69	68	66	61	67
Resident of Coyoacán	9259 (37)	9507 (38)	9527 (38)	9739 (39)	10187 (41)	48219 (38)
Resident of Iztapalapa	15858 (63)	15611(62)	15590 (62)	15379(61)	14930 (59)	77368 (62)
University or high school educated	3459 (14)	3647 (15)	3710 (15)	3912 (16)	4294 (17)	19022 (15)
**Lifestyle factors**						
Current smoker	6777 (27)	6806 (27)	6958 (28)	6994 (28)	7259 (29)	34794 (28)
Current alcohol drinker	16635 (66)	16670 (66)	16783 (67)	16573 (66)	16790 (67)	83451 (66)
Physical activity 1+ times/week	5364 (21)	5410 (22)	5467 (22)	5662 (23)	5982 (24)	27885 (22)
**Physical measurements, HbA1c, eGFR and fasting duration**						
Height, cm	155 (9)	156 (9)	156 (9)	156 (9)	156 (9)	156 (9)
Weight, kg	70.9 (13.3)	70.6 (13.2)	70.4 (13.1)	70.2 (12.9)	69.9 (12.9)	70.4 (13.1)
BMI, kg/m^2	29.4 (5.0)	29.2 (4.9)	29.1 (4.8)	28.9 (4.8)	28.6 (4.7)	29.0 (4.8)
Waist circumference, cm	95 (12)	95 (11)	94 (11)	94 (11)	94 (11)	94 (11)
Waist-to-hip ratio	0.90 (0.07)	0.90 (0.07)	0.90 (0.07)	0.90 (0.07)	0.90 (0.07)	0.90 (0.07)
Blood pressure, mmHg						
Systolic	127 (17)	127 (17)	127 (17)	127 (17)	127 (16)	127 (17)
Diastolic	83 (10)	83 (10)	83 (10)	83 (10)	83 (10)	83 (10)
HbA1c, %*	5.6 (0.8)	5.6 (0.9)	5.7 (1.0)	5.7 (1.0)	5.7 (1.1)	5.6 (1.0)
eGFR, mL/min/1.73 m^{2†}	98 (16)	98 (16)	98 (16)	97 (16)	97 (16)	97 (16)
Fasting duration, hours	4.0 (4.3)	3.9 (4.2)	4.0 (4.3)	3.9 (4.3)	3.9 (4.3)	3.9 (4.3)
**Medical history**						
Type 2 diabetes						
Previously diagnosed	2456 (10)	3040 (12)	3260 (13)	3779 (15)	4070 (16)	16605 (13)
Undiagnosed	975 (4)	1151 (5)	1300 (5)	1380 (5)	1539 (6)	6345 (5)
Total	3431 (14)	4191 (17)	4560 (18)	5159 (21)	5609 (22)	22950 (18)
Ischaemic heart disease	326 (1)	358 (1)	337 (1)	343 (1)	408 (2)	1772 (1)
Stroke	286 (1)	246 (1)	247 (1)	235 (1)	217 (1)	1231 (1)
Chronic kidney disease	182 (1)	154 (1)	190 (1)	184 (1)	204 (1)	914 (1)
Cirrhosis	45 (<0.5)	31 (<0.5)	32 (<0.5)	39 (<0.5)	31 (<0.5)	178 (<0.5)
Cancer	280 (1)	279 (1)	274 (1)	324 (1)	316 (1)	1473 (1)
Emphysema	85 (<0.5)	62 (<0.5)	66 (<0.5)	53 (<0.5)	86 (<0.5)	352 (<0.5)
**Long-term medication use**						
Any diabetes medication	1949 (8)	2388 (10)	2601 (10)	3006 (12)	3268 (13)	13212 (11)
Any anti-hypertensive medication	3828 (15)	3863 (15)	3806 (15)	3932 (16)	4056 (16)	19485 (16)
Any anti-thrombotic medication	708 (3)	717 (3)	711 (3)	759 (3)	791 (3)	3686 (3)

Mean (SD) or n (%)
*Among participants without diabetes; to convert % to mmol/mol, use the formula: HbA1c (mmol/mol) = 10.929 x (HbA1c [%] −2.15)
†Estimated using the Chronic Kidney Disease Epidemiology Collaboration 2009 creatinine equation (PMID: 34554658)
BMI=body mass index; eGFR=estimated glomerular filtration rate; GRS=genetic risk score

Supplementary Table S4: Baseline characteristics of 125,587 participants aged 35-84 years at recruitment by fifths of beta-cell -pro-insulin pathway type 2 diabetes GRS (72 SNPs)

	Fifth of type 2 diabetes GRS					Overall
	I	II	III	IV	V	
No. of participants	25117	25118	25117	25118	25117	125587
**Age, sex, ancestry and socioeconomic factors**						
Age, years	53 (12)	53 (12)	53 (12)	53 (12)	53 (12)	53 (12)
Men	8231 (33)	8217 (33)	8138 (32)	8209 (33)	8127 (32)	40922 (33)
Indigenous American ancestry, %	61	65	67	69	72	67
Resident of Coyoacán	10145 (40)	9593 (38)	9597 (38)	9591 (38)	9293 (37)	48219 (38)
Resident of Iztapalapa	14972 (60)	15525 (62)	15520 (62)	15527 (62)	15824 (63)	77368 (62)
University or high school educated	4297 (17)	3859 (15)	3851 (15)	3683 (15)	3332 (13)	19022 (15)
**Lifestyle factors**						
Current smoker	7264 (28)	7055 (28)	6953 (28)	6811 (27)	6711 (27)	34794 (28)
Current alcohol drinker	16848 (67)	16753 (67)	16714 (67)	16691 (66)	16445 (65)	83451 (66)
Physical activity 1+ times/week	5821 (23)	5648 (22)	5555 (22)	5531 (22)	5330 (21)	27885 (22)
**Physical measurements, HbA1c, eGFR and fasting duration**						
Height, cm	156 (9)	156 (9)	156 (9)	155 (9)	155 (9)	156 (9)
Weight, kg	71.3 (13.3)	70.7 (13.1)	70.3 (13.1)	70.2 (13.0)	69.4 (12.7)	70.4 (13.1)
BMI, kg/m^2	29.2 (5.0)	29.1 (4.9)	29.1 (4.9)	29.0 (4.8)	28.9 (4.7)	29.0 (4.8)
Waist circumference, cm	95 (12)	95 (11)	94 (11)	94 (11)	94 (11)	94 (11)
Waist-to-hip ratio	0.90 (0.07)	0.90 (0.07)	0.90 (0.07)	0.90 (0.07)	0.90 (0.07)	0.90 (0.07)
Blood pressure, mmHg						
Systolic	128 (17)	127 (17)	127 (17)	127 (17)	127 (17)	127 (17)
Diastolic	83 (10)	83 (10)	83 (10)	83 (10)	83 (10)	83 (10)
HbA1c, %*	5.6 (0.9)	5.6 (0.9)	5.6 (1.0)	5.7 (1.0)	5.7 (1.0)	5.6 (1.0)
eGFR, mL/min/1.73 $m^{2\dagger}$	97 (16)	97 (16)	98 (16)	98 (16)	98 (16)	97 (16)
Fasting duration, hours	3.9 (4.3)	3.9 (4.3)	3.9 (4.3)	3.9 (4.3)	3.9 (4.3)	3.9 (4.3)
**Medical history**						
Type 2 diabetes						
Previously diagnosed	2457 (10)	2971 (12)	3325 (13)	3576 (14)	4276 (17)	16605 (13)
Undiagnosed	1052 (4)	1187 (5)	1268 (5)	1377 (5)	1461 (6)	6345 (5)
Total	3509 (14)	4158 (17)	4593 (18)	4953 (20)	5737 (23)	22950 (18)
Ischaemic heart disease	375 (1)	386 (2)	347 (1)	343 (1)	321 (1)	1772 (1)
Stroke	231 (1)	254 (1)	258 (1)	253 (1)	235 (1)	1231 (1)
Chronic kidney disease	176 (1)	200 (1)	190 (1)	167 (1)	181 (1)	914 (1)
Cirrhosis	36 (<0.5)	33 (<0.5)	35 (<0.5)	32 (<0.5)	42 (<0.5)	178 (<0.5)
Cancer	328 (1)	312 (1)	293 (1)	263 (1)	277 (1)	1473 (1)
Emphysema	95 (<0.5)	53 (<0.5)	79 (<0.5)	62 (<0.5)	63 (<0.5)	352 (<0.5)
**Long-term medication use**						
Any diabetes medication	1921 (8)	2362 (9)	2628 (10)	2842 (11)	3459 (14)	13212 (11)
Any anti-hypertensive medication	3996 (16)	3992 (16)	3807 (15)	3867 (15)	3823 (15)	19485 (16)
Any anti-thrombotic medication	806 (3)	765 (3)	728 (3)	717 (3)	670 (3)	3686 (3)

Mean (SD) or n (%)
*Among participants without diabetes; to convert % to mmol/mol, use the formula: HbA1c (mmol/mol) = 10.929 x (HbA1c [%] −2.15)
†Estimated using the Chronic Kidney Disease Epidemiology Collaboration 2009 creatinine equation (PMID: 34554658)
BMI=body mass index; eGFR=estimated glomerular filtration rate; GRS=genetic risk score

Supplementary Table S5: Baseline characteristics of 125,587 participants aged 35-84 years at recruitment by fifths of residual glycaemic pathway type 2 diabetes GRS (306 SNPs)

	Fifth of type 2 diabetes GRS					Overall
	**I**	**II**	**III**	**IV**	**V**	
No. of participants	25117	25118	25117	25118	25117	125587
**Age, sex, ancestry and socioeconomic factors**						
Age, years	53 (13)	53 (12)	53 (12)	53 (12)	52 (12)	53 (12)
Men	8260 (33)	8166 (33)	8154 (32)	8206 (33)	8136 (32)	40922 (33)
Indigenous American ancestry, %	64	66	67	68	69	67
Resident of Coyoacán	9787 (39)	9716 (39)	9705 (39)	9501 (38)	9510 (38)	48219 (38)
Resident of Iztapalapa	15330 (61)	15402 (61)	15412 (61)	15617 (62)	15607 (62)	77368 (62)
University or high school educated	4081 (16)	3872 (15)	3702 (15)	3746 (15)	3621 (14)	19022 (15)
**Lifestyle factors**						
Current smoker	6964 (28)	6928 (28)	7022 (28)	6924 (28)	6956 (28)	34794 (28)
Current alcohol drinker	16851 (67)	16582 (66)	16763 (67)	16604 (66)	16651 (66)	83451 (66)
Physical activity 1+ times/week	5806 (23)	5674 (23)	5457 (22)	5468 (22)	5480 (22)	27885 (22)
**Physical measurements, HbA1c, eGFR and fasting duration**						
Height, cm	156 (9)	156 (9)	156 (9)	156 (9)	156 (9)	156 (9)
Weight, kg	70.6 (13.3)	70.5 (13.0)	70.5 (13.1)	70.3 (13.0)	70.1 (12.9)	70.4 (13.1)
BMI, kg/m^2	29.1 (4.9)	29.1 (4.8)	29.1 (4.9)	29.0 (4.8)	29.0 (4.7)	29.0 (4.8)
Waist circumference, cm	95 (11)	95 (11)	94 (11)	94 (11)	94 (11)	94 (11)
Waist-to-hip ratio	0.90 (0.07)	0.90 (0.07)	0.90 (0.07)	0.90 (0.07)	0.90 (0.07)	0.90 (0.07)
Blood pressure, mmHg						
Systolic	127 (17)	127 (17)	127 (17)	127 (17)	127 (17)	127 (17)
Diastolic	83 (10)	83 (10)	83 (10)	83 (10)	83 (10)	83 (10)
HbA1c, %*	5.6 (0.9)	5.6 (0.9)	5.6 (1.0)	5.7 (1.1)	5.7 (1.1)	5.6 (1.0)
eGFR, mL/min/1.73 m^2†	97 (16)	97 (16)	98 (16)	98 (16)	98 (16)	97 (16)
Fasting duration, hours	3.9 (4.3)	3.9 (4.3)	3.9 (4.3)	3.9 (4.3)	3.9 (4.3)	3.9 (4.3)
**Medical history**						
Type 2 diabetes						
Previously diagnosed	2481 (10)	3050 (12)	3307 (13)	3590 (14)	4177 (17)	16605 (13)
Undiagnosed	1026 (4)	1154 (5)	1265 (5)	1373 (5)	1527 (6)	6345 (5)
Total	3507 (14)	4204 (17)	4572 (18)	4963 (20)	5704 (23)	22950 (18)
Ischaemic heart disease	376 (1)	364 (1)	380 (2)	318 (1)	334 (1)	1772 (1)
Stroke	234 (1)	254 (1)	243 (1)	255 (1)	245 (1)	1231 (1)
Chronic kidney disease	187 (1)	194 (1)	180 (1)	172 (1)	181 (1)	914 (1)
Cirrhosis	38 (<0.5)	33 (<0.5)	34 (<0.5)	38 (<0.5)	35 (<0.5)	178 (<0.5)
Cancer	307 (1)	324 (1)	283 (1)	293 (1)	266 (1)	1473 (1)
Emphysema	69 (<0.5)	80 (<0.5)	71 (<0.5)	63 (<0.5)	69 (<0.5)	352 (<0.5)
**Long-term medication use**						
Any diabetes medication	1955 (8)	2395 (10)	2659 (11)	2868 (11)	3335 (13)	13212 (11)
Any anti-hypertensive medication	4009 (16)	3932 (16)	3910 (16)	3810 (15)	3824 (15)	19485 (16)
Any anti-thrombotic medication	821 (3)	768 (3)	701 (3)	715 (3)	681 (3)	3686 (3)

Mean (SD) or n (%)
*Among participants without diabetes; to convert % to mmol/mol, use the formula: HbA1c (mmol/mol) = 10.929 x (HbA1c [%] −2.15)
†Estimated using the Chronic Kidney Disease Epidemiology Collaboration 2009 creatinine equation (PMID: 34554658)
BMI=body mass index; eGFR=estimated glomerular filtration rate; GRS=genetic risk score

Supplementary Table S6: Baseline characteristics of 125,587 participants aged 35-84 years at recruitment by fifths of body fat pathway type 2 diabetes GRS (220 SNPs)

	Fifth of type 2 diabetes GRS					Overall
	I	II	III	IV	V	
No. of participants	25117	25118	25117	25118	25117	125587
**Age, sex, ancestry and socioeconomic factors**						
Age, years	53 (12)	53 (12)	53 (12)	53 (12)	53 (12)	53 (12)
Men	8105 (32)	8176 (33)	8159 (32)	8174 (33)	8308 (33)	40922 (33)
Indigenous American ancestry, %	71	69	67	66	62	67
Resident of Coyoacán	9357 (37)	9418 (37)	9524 (38)	9947 (40)	9973 (40)	48219 (38)
Resident of Iztapalapa	15760 (63)	15700 (63)	15593 (62)	15171 (60)	15144 (60)	77368 (62)
University or high school educated	3555 (14)	3828 (15)	3719 (15)	3908 (16)	4012 (16)	19022 (15)
**Lifestyle factors**						
Current smoker	6498 (26)	6805 (27)	6976 (28)	7086 (28)	7429 (30)	34794 (28)
Current alcohol drinker	16591 (66)	16704 (67)	16687 (66)	16701 (66)	16768 (67)	83451 (66)
Physical activity 1+ times/week	5395 (21)	5462 (22)	5553 (22)	5657 (23)	5818 (23)	27885 (22)
**Physical measurements, HbA1c, eGFR and fasting duration**						
Height, cm	155 (9)	156 (9)	156 (9)	156 (9)	156 (9)	156 (9)
Weight, kg	70.3 (13.0)	70.5 (13.1)	70.4 (13.1)	70.3 (13.1)	70.5 (13.1)	70.4 (13.1)
BMI, kg/m^2	29.1 (4.8)	29.2 (4.9)	29.0 (4.8)	29.0 (4.9)	28.9 (4.9)	29.0 (4.8)
Waist circumference, cm	95 (11)	95 (11)	94 (11)	94 (11)	94 (11)	94 (11)
Waist-to-hip ratio	0.90 (0.07)	0.90 (0.07)	0.90 (0.07)	0.90 (0.07)	0.90 (0.07)	0.90 (0.07)
Blood pressure, mmHg						
Systolic	127 (17)	127 (17)	127 (17)	127 (17)	128 (17)	127 (17)
Diastolic	83 (10)	83 (10)	83 (10)	83 (10)	83 (10)	83 (10)
HbA1c, %*	5.6 (0.9)	5.6 (1.0)	5.7 (1.0)	5.7 (1.0)	5.7 (1.0)	5.6 (1.0)
eGFR, mL/min/1.73 m^2†	98 (16)	98 (16)	97 (16)	97 (16)	97 (17)	97 (16)
Fasting duration, hours	3.9 (4.3)	3.9 (4.3)	4.0 (4.4)	3.9 (4.3)	3.9 (4.3)	3.9 (4.3)
**Medical history**						
Type 2 diabetes						
Previously diagnosed	2746 (11)	3103 (12)	3307 (13)	3521 (14)	3928 (17)	16605 (13)
Undiagnosed	1081 (4)	1288 (5)	1298 (5)	1290 (5)	1388 (6)	6345 (5)
Total	3827 (15)	4391 (17)	4605 (18)	4811 (19)	5316 (21)	22950 (18)
Ischaemic heart disease	318 (1)	335 (1)	336 (1)	369 (1)	414 (2)	1772 (1)
Stroke	243 (1)	255 (1)	235 (1)	243 (1)	255 (1)	1231 (1)
Chronic kidney disease	157 (1)	196 (1)	201 (1)	176 (1)	184 (1)	914 (1)
Cirrhosis	38 (<0.5)	39 (<0.5)	27 (<0.5)	43 (<0.5)	31 (<0.5)	178 (<0.5)
Cancer	289 (1)	300 (1)	283 (1)	286 (1)	315 (1)	1473 (1)
Emphysema	65 (<0.5)	66 (<0.5)	67 (<0.5)	75 (<0.5)	79 (<0.5)	352 (<0.5)
**Long-term medication use**						
Any diabetes medication	2197 (9)	2485 (10)	2626 (10)	2791 (11)	3113 (12)	13212 (11)
Any anti-hypertensive medication	3752 (15)	3821 (15)	3849 (15)	3922 (16)	4141 (16)	19485 (16)
Any anti-thrombotic medication	724 (3)	695 (3)	750 (3)	741 (3)	776 (3)	3686 (3)

Mean (SD) or n (%)
*Among participants without diabetes; to convert % to mmol/mol, use the formula: HbA1c (mmol/mol) = 10.929 x (HbA1c [%] −2.15)
†Estimated using the Chronic Kidney Disease Epidemiology Collaboration 2009 creatinine equation (PMID: 34554658)
BMI=body mass index; eGFR=estimated glomerular filtration rate; GRS=genetic risk score

Supplementary Table S7: Baseline characteristics of 125,587 participants aged 35-84 years at recruitment by fifths of metabolic syndrome pathway type 2 diabetes GRS (137 SNPs)

	Fifth of type 2 diabetes GRS					Overall
	I	II	III	IV	V	
No. of participants	25117	25118	25117	25118	25117	125587
**Age, sex, ancestry and socioeconomic factors**						
Age, years	53 (12)	53 (12)	53 (12)	53 (12)	53 (12)	53 (12)
Men	8324 (33)	8220 (33)	8131 (32)	8046 (32)	8201 (33)	40922 (33)
Indigenous American ancestry, %	63	66	68	69	70	67
Resident of Coyoacán	10057 (40)	9743 (39)	9553 (38)	9489 (38)	9377 (37)	48219 (38)
Resident of Iztapalapa	15060 (60)	15375 (61)	15564 (62)	15629 (62)	15740 (63)	77368 (62)
University or high school educated	4147 (17)	3833 (15)	3742 (15)	3698 (15)	3602 (14)	19022 (15)
**Lifestyle factors**						
Current smoker	7114 (28)	6965 (28)	6974 (28)	6918 (28)	6823 (27)	34794 (28)
Current alcohol drinker	16992 (68)	16770 (67)	16569 (66)	16732 (67)	16388 (65)	83451 (66)
Physical activity 1+ times/week	5860 (23)	5588 (22)	5565 (22)	5482 (22)	5390 (21)	27885 (22)
**Physical measurements, HbA1c, eGFR and fasting duration**						
Height, cm	156 (9)	156 (9)	156 (9)	155 (9)	155 (9)	156 (9)
Weight, kg	71.5 (13.4)	70.8 (13.2)	70.4 (13.0)	69.9 (12.9)	69.4 (12.7)	70.4 (13.1)
BMI, kg/m^2	29.2 (4.9)	29.2 (4.9)	29.1 (4.8)	29.0 (4.8)	28.8 (4.7)	29.0 (4.8)
Waist circumference, cm	95 (12)	95 (11)	94 (11)	94 (11)	94 (11)	94 (11)
Waist-to-hip ratio	0.90 (0.07)	0.90 (0.07)	0.90 (0.07)	0.90 (0.07)	0.90 (0.07)	0.90 (0.07)
Blood pressure, mmHg						
Systolic	127 (16)	127 (17)	127 (17)	127 (17)	127 (17)	127 (17)
Diastolic	83 (10)	83 (10)	83 (10)	83 (10)	83 (10)	83 (10)
HbA1c, %*	5.6 (0.9)	5.6 (0.9)	5.6 (1.0)	5.7 (1.0)	5.7 (1.1)	5.6 (1.0)
eGFR, mL/min/1.73 $m^{2†}$	97 (16)	98 (16)	98 (16)	98 (16)	98 (16)	97 (16)
Fasting duration, hours	3.9 (4.3)	3.9 (4.3)	3.9 (4.3)	3.9 (4.3)	4.0 (4.3)	3.9 (4.3)
**Medical history**						
Type 2 diabetes						
Previously diagnosed	2520 (10)	2936 (12)	3341 (13)	3659 (15)	4149 (17)	16605 (13)
Undiagnosed	1019 (4)	1148 (5)	1228 (5)	1382 (6)	1568 (6)	6345 (5)
Total	3539 (14)	4084 (16)	4569 (18)	5041 (20)	5717 (23)	22950 (18)
Ischaemic heart disease	411 (2)	308 (1)	360 (1)	344 (1)	349 (1)	1772 (1)
Stroke	230 (1)	254 (1)	231 (1)	249 (1)	267 (1)	1231 (1)
Chronic kidney disease	178 (1)	187 (1)	189 (1)	169 (1)	191 (1)	914 (1)
Cirrhosis	28 (<0.5)	49 (<0.5)	42 (<0.5)	23 (<0.5)	36 (<0.5)	178 (<0.5)
Cancer	318 (1)	304 (1)	325 (1)	253 (1)	273 (1)	1473 (1)
Emphysema	87 (<0.5)	78 (<0.5)	75 (<0.5)	53 (<0.5)	59 (<0.5)	352 (<0.5)
**Long-term medication use**						
Any diabetes medication	1981 (8)	2324 (9)	2627 (10)	2952 (12)	3328 (13)	13212 (11)
Any anti-hypertensive medication	3965 (16)	3951 (16)	3860 (15)	3845 (15)	3864 (15)	19485 (16)
Any anti-thrombotic medication	812 (3)	755 (3)	716 (3)	714 (3)	689 (3)	3686 (3)

Mean (SD) or n (%)

*Among participants without diabetes; to convert % to mmol/mol, use the formula: HbA1c (mmol/mol) = 10.929 x (HbA1c [%] −2.15)

†Estimated using the Chronic Kidney Disease Epidemiology Collaboration 2009 creatinine equation (PMID: 34554658)

BMI=body mass index; eGFR=estimated glomerular filtration rate; GRS=genetic risk score

Supplementary Table S8: Baseline characteristics of 125,587 participants aged 35-84 years at recruitment by fifths of obesity pathway type 2 diabetes GRS (203 SNPs)

	Fifth of type 2 diabetes GRS					Overall
	I	II	III	IV	V	
No. of participants	25117	25118	25117	25118	25117	125587
**Age, sex, ancestry and socioeconomic factors**						
Age, years	53 (13)	53 (12)	53 (12)	52 (12)	52 (12)	53 (12)
Men	8249 (33)	8241 (33)	8014 (32)	8214 (33)	8204 (33)	40922 (33)
Indigenous American ancestry, %	60	65	68	70	72	67
Resident of Coyoacán	10538 (42)	9827 (39)	9555 (38)	9321 (37)	8978 (36)	48219 (38)
Resident of Iztapalapa	14579 (58)	15291 (61)	15562 (62)	15797 (63)	16139 (64)	77368 (62)
University or high school educated	4540 (18)	3899 (16)	3732 (15)	3530 (14)	3321 (13)	19022 (15)
**Lifestyle factors**						
Current smoker	7033 (28)	7027 (28)	6789 (27)	7006 (28)	6939 (28)	34794 (28)
Current alcohol drinker	17107 (68)	16769 (67)	16606 (66)	16582 (66)	16387 (65)	83451 (66)
Physical activity 1+ times/week	6196 (25)	5747 (23)	5452 (22)	5326 (21)	5164 (21)	27885 (22)
**Physical measurements, HbA1c, eGFR and fasting duration**						
Height, cm	156 (9)	156 (9)	155 (9)	155 (9)	155 (9)	156 (9)
Weight, kg	69.3 (12.8)	69.8 (13.0)	70.3 (13.0)	71.0 (13.1)	71.7 (13.3)	70.4 (13.1)
BMI, kg/m^2	28.3 (4.7)	28.7 (4.7)	29.1 (4.8)	29.4 (4.9)	29.7 (4.9)	29.0 (4.8)
Waist circumference, cm	93 (11)	94 (11)	94 (11)	95 (11)	96 (11)	94 (11)
Waist-to-hip ratio	0.90 (0.07)	0.90 (0.07)	0.90 (0.07)	0.91 (0.07)	0.91 (0.07)	0.90 (0.07)
Blood pressure, mmHg						
Systolic	127 (17)	127 (17)	127 (17)	128 (17)	128 (17)	127 (17)
Diastolic	83 (10)	83 (10)	83 (10)	83 (10)	83 (10)	83 (10)
HbA1c, %*	5.6 (0.8)	5.6 (0.9)	5.6 (1.0)	5.7 (1.0)	5.7 (1.1)	5.6 (1.0)
eGFR, mL/min/1.73 m^{2†}	96 (16)	97 (16)	97 (16)	98 (16)	98 (16)	97 (16)
Fasting duration, hours	3.9 (4.3)	3.9 (4.3)	3.9 (4.3)	4.0 (4.3)	3.9 (4.3)	3.9 (4.3)
**Medical history**						
Type 2 diabetes						
Previously diagnosed	2500 (10)	2959 (12)	3323 (13)	3526 (14)	4297 (17)	16605 (13)
Undiagnosed	993 (4)	1150 (5)	1267 (5)	1368 (5)	1567 (6)	6345 (5)
Total	3493 (14)	4109 (16)	4590 (18)	4894 (19)	5864 (23)	22950 (18)
Ischaemic heart disease	373 (1)	350 (1)	336 (1)	340 (1)	373 (1)	1772 (1)
Stroke	237 (1)	220 (1)	259 (1)	257 (1)	258 (1)	1231 (1)
Chronic kidney disease	181 (1)	169 (1)	187 (1)	203 (1)	174 (1)	914 (1)
Cirrhosis	30 (<0.5)	37 (<0.5)	27 (<0.5)	42 (<0.5)	42 (<0.5)	178 (<0.5)
Cancer	318 (1)	293 (1)	302 (1)	266 (1)	294 (1)	1473 (1)
Emphysema	108 (<0.5)	56 (<0.5)	66 (<0.5)	64 (<0.5)	58 (<0.5)	352 (<0.5)
**Long-term medication use**						
Any diabetes medication	1967 (8)	2336 (9)	2652 (11)	2795 (11)	3462 (14)	13212 (11)
Any anti-hypertensive medication	3849 (15)	3923 (16)	3854 (15)	3787 (15)	4072 (16)	19485 (16)
Any anti-thrombotic medication	830 (3)	764 (3)	700 (3)	698 (3)	694 (3)	3686 (3)

Mean (SD) or n (%)
*Among participants without diabetes; to convert % to mmol/mol, use the formula: HbA1c (mmol/mol) = 10.929 x (HbA1c [%] −2.15)
†Estimated using the Chronic Kidney Disease Epidemiology Collaboration 2009 creatinine equation (PMID: 34554658)
BMI=body mass index; eGFR=estimated glomerular filtration rate; GRS=genetic risk score

Supplementary Table S9: Baseline characteristics of 125,587 participants aged 35-84 years at recruitment by fifths of lipodystrophy pathway type 2 diabetes GRS (37 SNPs)

	Fifth of type 2 diabetes GRS					Overall
	I	II	III	IV	V	
No. of participants	25117	25118	25117	25118	25117	125587
**Age, sex, ancestry and socioeconomic factors**						
Age, years	53 (12)	53 (12)	53 (12)	53 (12)	52 (12)	53 (12)
Men	8333 (33)	8149 (32)	8107 (32)	8305 (33)	8028 (32)	40922 (33)
Indigenous American ancestry, %	63	66	68	69	70	67
Resident of Coyoacán	10020 (40)	9800 (39)	9514 (38)	9455 (38)	9430 (38)	48219 (38)
Resident of Iztapalapa	15097 (60)	15318 (61)	15603 (62)	15663 (62)	15687 (62)	77368 (62)
University or high school educated	4160 (17)	3852 (15)	3754 (15)	3647 (15)	3609 (14)	19022 (15)
**Lifestyle factors**						
Current smoker	7264 (29)	7034 (28)	6890 (27)	6868 (27)	6738 (27)	34794 (28)
Current alcohol drinker	16864 (67)	16823 (67)	16655 (66)	16651 (66)	16458 (66)	83451 (66)
Physical activity 1+ times/week	5785 (23)	5652 (23)	5495 (22)	5497 (22)	5456 (22)	27885 (22)
**Physical measurements, HbA1c, eGFR and fasting duration**						
Height, cm	156 (9)	156 (9)	156 (9)	156 (9)	155 (9)	156 (9)
Weight, kg	71.7 (13.6)	70.9 (13.2)	70.2 (12.8)	70.0 (12.9)	69.3 (12.8)	70.4 (13.1)
BMI, kg/m^2	29.4 (5.1)	29.2 (4.9)	29.0 (4.8)	28.9 (4.7)	28.7 (4.6)	29.0 (4.8)
Waist circumference, cm	95 (12)	95 (11)	94 (11)	94 (11)	94 (11)	94 (11)
Waist-to-hip ratio	0.90 (0.07)	0.90 (0.07)	0.90 (0.07)	0.90 (0.07)	0.90 (0.07)	0.90 (0.07)
Blood pressure, mmHg						
Systolic	127 (17)	127 (17)	127 (17)	127 (16)	128 (17)	127 (17)
Diastolic	83 (10)	83 (10)	83 (10)	83 (10)	83 (10)	83 (10)
HbA1c, %*	5.6 (0.9)	5.6 (0.9)	5.6 (1.0)	5.7 (1.0)	5.7 (1.1)	5.6 (1.0)
eGFR, mL/min/1.73 m^{2†}	97 (16)	97 (16)	98 (16)	98 (16)	98 (16)	97 (16)
Fasting duration, hours	3.9 (4.3)	3.9 (4.3)	3.9 (4.3)	3.9 (4.3)	3.9 (4.2)	3.9 (4.3)
**Medical history**						
Type 2 diabetes						
Previously diagnosed	2816 (11)	3059 (12)	3285 (13)	3513 (14)	3932 (16)	16605 (13)
Undiagnosed	1106 (4)	1198 (5)	1296 (5)	1309 (5)	1436 (6)	6345 (5)
Total	3922 (16)	4257 (17)	4581 (18)	4822 (19)	5368 (21)	22950 (18)
Ischaemic heart disease	389 (2)	384 (2)	331 (1)	346 (1)	322 (1)	1772 (1)
Stroke	250 (1)	239 (1)	235 (1)	234 (1)	273 (1)	1231 (1)
Chronic kidney disease	178 (1)	194 (1)	172 (1)	184 (1)	186 (1)	914 (1)
Cirrhosis	38 (<0.5)	30 (<0.5)	35 (<0.5)	33 (<0.5)	42 (<0.5)	178 (<0.5)
Cancer	308 (1)	291 (1)	295 (1)	293 (1)	286 (1)	1473 (1)
Emphysema	82 (<0.5)	75 (<0.5)	71 (<0.5)	68 (<0.5)	56 (<0.5)	352 (<0.5)
**Long-term medication use**						
Any diabetes medication	2210 (9)	2382 (9)	2652 (11)	2790 (11)	3178 (13)	13212 (11)
Any anti-hypertensive medication	3865 (15)	3959 (16)	3891 (15)	3905 (16)	3865 (15)	19485 (16)
Any anti-thrombotic medication	768 (3)	736 (3)	749 (3)	732 (3)	701 (3)	3686 (3)

Mean (SD) or n (%)
*Among participants without diabetes; to convert % to mmol/mol, use the formula: HbA1c (mmol/mol) = 10.929 x (HbA1c [%] −2.15)
†Estimated using the Chronic Kidney Disease Epidemiology Collaboration 2009 creatinine equation (PMID: 34554658)
BMI=body mass index; eGFR=estimated glomerular filtration rate; GRS=genetic risk score

Supplementary Table S10: Baseline characteristics of 125,587 participants aged 35-84 years at recruitment by fifths of liver and lipid metabolism pathway type 2 diabetes GRS (2 SNPs)

	Fifth of type 2 diabetes GRS					Overall
	I	II	III	IV	V	
No. of participants	29482	20988	18420	31782	24915	125587
**Age, sex, ancestry and socioeconomic factors**						
Age, years	53 (12)	53 (12)	53 (13)	53 (12)	52 (12)	53 (12)
Men	9620 (33)	6747 (32)	6035 (33)	10314 (32)	8206 (33)	40922 (33)
Indigenous American ancestry, %	61	68	66	69	72	67
Resident of Coyoacán	11857 (40)	8028 (38)	7119 (39)	11995 (38)	9220 (37)	48219 (38)
Resident of Iztapalapa	17625 (60)	12960 (62)	11301 (61)	19787 (62)	15695 (63)	77368 (62)
University or high school educated	5090 (17)	3196 (15)	2842 (15)	4647 (15)	3247 (13)	19022 (15)
**Lifestyle factors**						
Current smoker	8741 (30)	5747 (27)	5141 (28)	8444 (27)	6721 (27)	34794 (28)
Current alcohol drinker	19746 (67)	13945 (66)	12302 (67)	21029 (66)	16429 (66)	83451 (66)
Physical activity 1+ times/week	7021 (24)	4682 (23)	4099 (22)	6886 (22)	5197 (21)	27885 (22)
**Physical measurements, HbA1c, eGFR and fasting duration**						
Height, cm	156 (9)	155 (9)	156 (9)	155 (9)	155 (9)	156 (9)
Weight, kg	70.9 (13.2)	70.3 (13.0)	70.5 (13.1)	70.3 (13.1)	70.0 (19.1)	70.4 (13.1)
BMI, kg/m^2	29.0 (5.0)	29.1 (4.8)	29.0 (4.8)	29.1 (4.8)	29.0 (4.8)	29.0 (4.8)
Waist circumference, cm	94 (12)	94 (11)	94 (11)	95 (11)	94 (11)	94 (11)
Waist-to-hip ratio	0.90 (0.07)	0.90 (0.07)	0.90 (0.07)	0.90 (0.07)	0.91 (0.07)	0.90 (0.07)
Blood pressure, mmHg						
Systolic	128 (17)	127 (17)	127 (17)	127 (17)	127 (17)	127 (17)
Diastolic	83 (10)	83 (10)	83 (10)	83 (10)	83 (10)	83 (10)
HbA1c, %*	5.6 (0.9)	5.6 (0.9)	5.6 (1.0)	5.7 (1.0)	5.7 (1.0)	5.6 (1.0)
eGFR, mL/min/1.73 m^2†	97 (16)	98 (16)	97 (16)	98 (16)	98 (16)	97 (16)
Fasting duration, hours	3.9 (4.3)	3.9 (4.3)	4.0 (4.3)	3.9 (4.3)	3.9 (4.3)	3.9 (4.3)
**Medical history**						
Type 2 diabetes						
Previously diagnosed	3567 (12)	2752 (13)	2339 (13)	4314 (14)	3633 (15)	16605 (13)
Undiagnosed	1381 (5)	1056 (5)	918 (5)	1624 (5)	1366 (5)	6345 (5)
Total	4948 (17)	3808 (18)	3257 (18)	5938 (19)	4999 (20)	22950 (18)
Ischaemic heart disease	474 (2)	275 (1)	300 (2)	427 (1)	296 (1)	1772 (1)
Stroke	328 (1)	181 (1)	162 (1)	317 (1)	243 (1)	1231 (1)
Chronic kidney disease	228 (1)	130 (1)	133 (1)	236 (1)	187 (1)	914 (1)
Cirrhosis	36 (<0.5)	25 (<0.5)	18 (<0.5)	45 (<0.5)	54 (<0.5)	178 (<0.5)
Cancer	399 (1)	256 (1)	215 (1)	352 (1)	251 (1)	1473 (1)
Emphysema	112 (<0.5)	51 (<0.5)	55 (<0.5)	93 (<0.5)	41 (<0.5)	352 (<0.5)
**Long-term medication use**						
Any diabetes medication	2840 (10)	2184 (10)	1859 (10)	3419 (11)	2910 (12)	13212 (11)
Any anti-hypertensive medication	5020 (17)	3322 (16)	2853 (15)	4767 (15)	3523 (14)	19485 (16)
Any anti-thrombotic medication	970 (3)	614 (3)	558 (3)	889 (3)	655 (3)	3686 (3)

Mean (SD) or n (%)
*Among participants without diabetes; to convert % to mmol/mol, use the formula: HbA1c (mmol/mol) = 10.929 x (HbA1c [%] −2.15)
†Estimated using the Chronic Kidney Disease Epidemiology Collaboration 2009 creatinine equation (PMID: 34554658)
BMI=body mass index; eGFR=estimated glomerular filtration rate; GRS=genetic risk score

Supplementary Figure S1: Genetic variant effect size estimates for type 2 diabetes in Mexico City Prospective Study and in T2D Global Genomics Initiative multi-ancestry GWAS

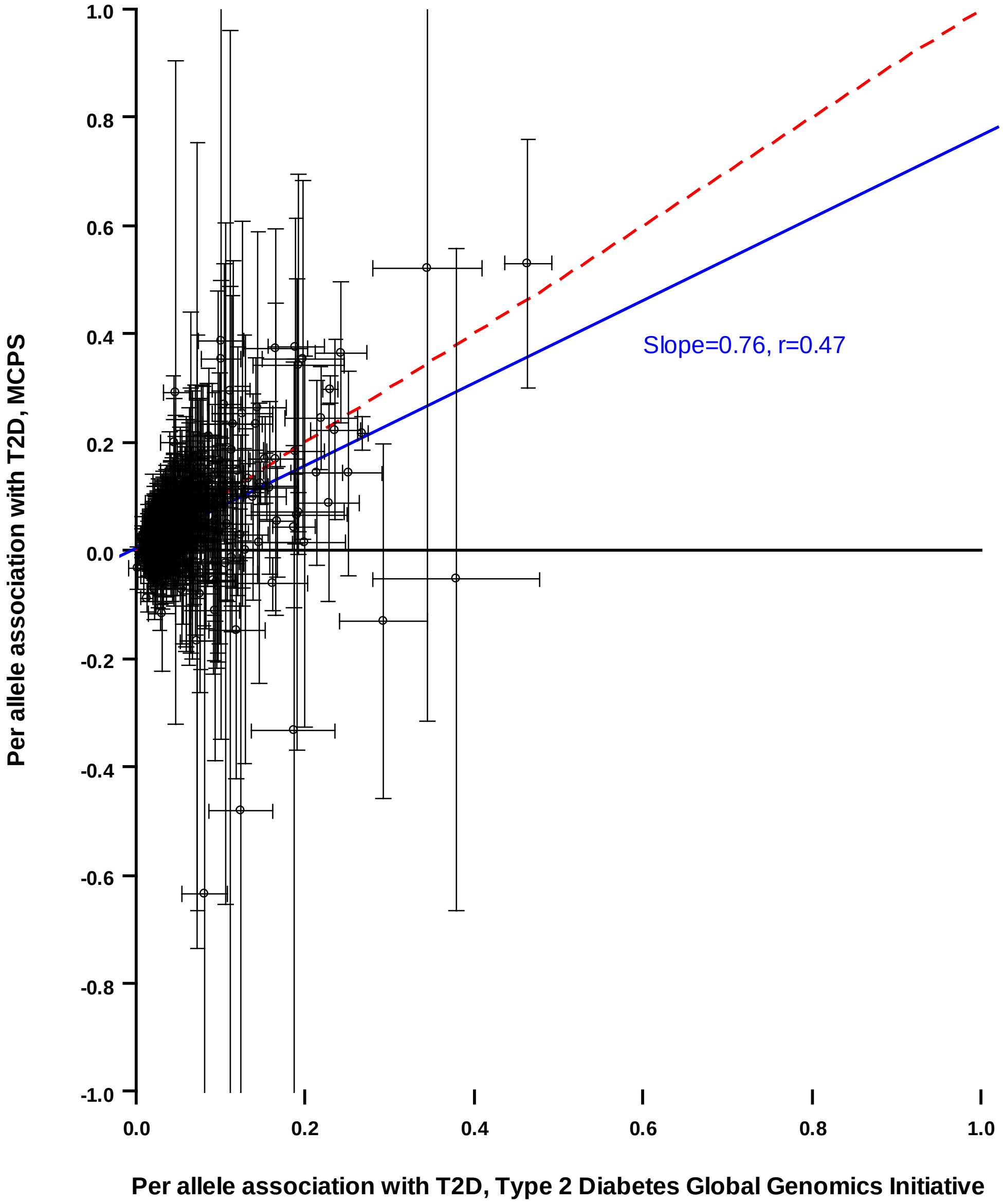

Red dashed line is line of equality. Blue line is line of best fit from a linear regression. Type 2 Diabetes Global Genomics Initiative: Nature 2024;627(8003):347-357. MCPS=Mexico City Prospective Study; r=Pearson correlation coefficient; T2D=type 2 diabetes.

Supplementary Figure S2: Association of type 2 diabetes GRS with observed type 2 diabetes status among 125,587 participants aged 35-84 years at recruitment

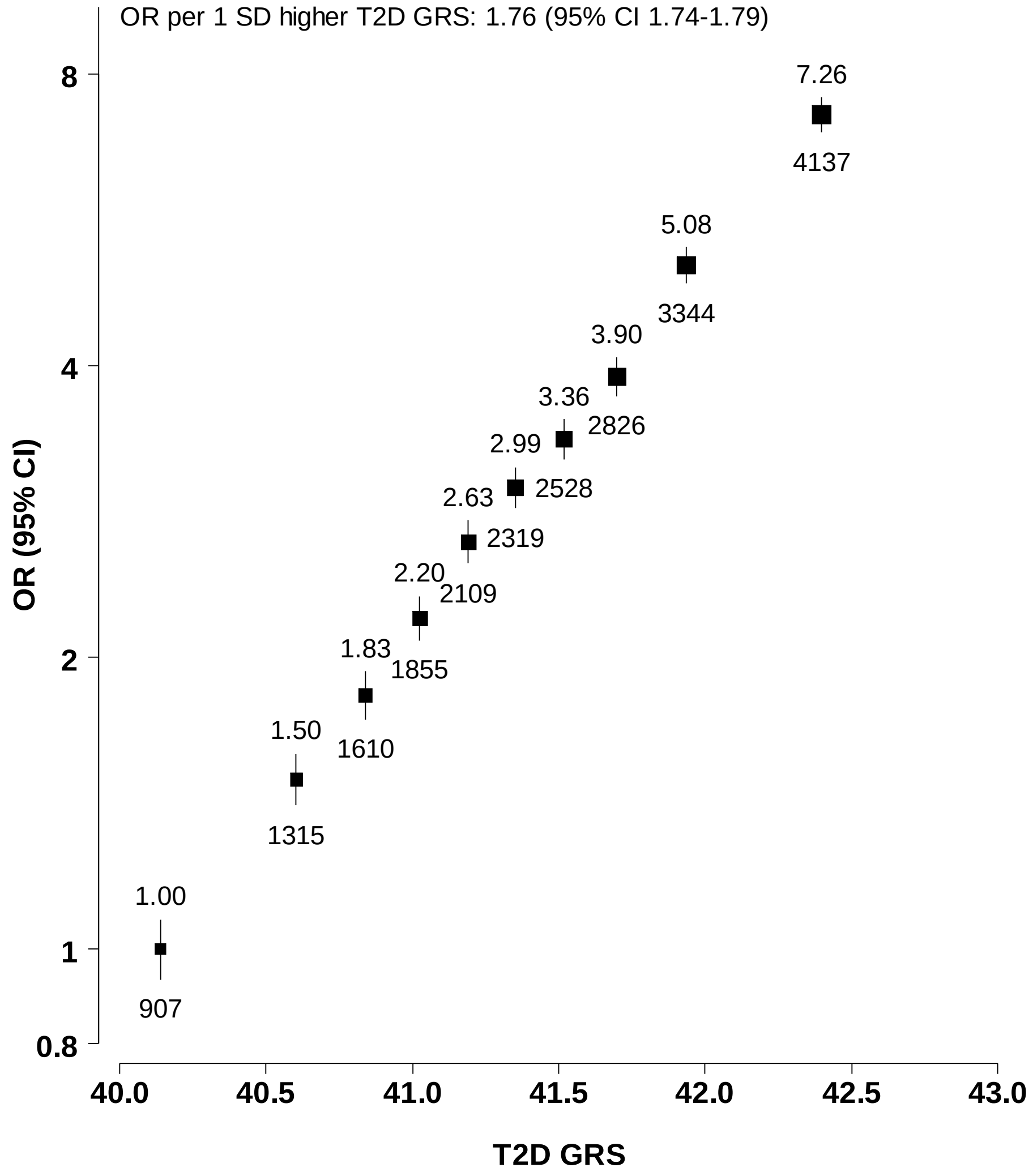

Observed type 2 diabetes (T2D) status refers to previously diagnosed or undiagnosed T2D at recruitment. The T2D genetic risk score (GRS) represents the sum across all SNPs of their effect allele count multiplied by the log odds ratio (OR) for the association of the SNP with T2D in the T2D Global Genomics Initiative multi-ancestry meta-regression. T2D GRS SD=0.64 units. T2D ORs are adjusted for age, sex and the first 7 principal components. The numbers above the squares are the ORs and the numbers below the squares are the number of participants with T2D in that group. The size of each square is proportional to the amount of data available. Error bars represent 95% confidence intervals (CI).

Supplementary Figure S3: Association of type 2 diabetes GRS with observed type 2 diabetes status by sex, age and Indigenous American ancestry proportion among 125,587 participants aged 35-84 years at recruitment

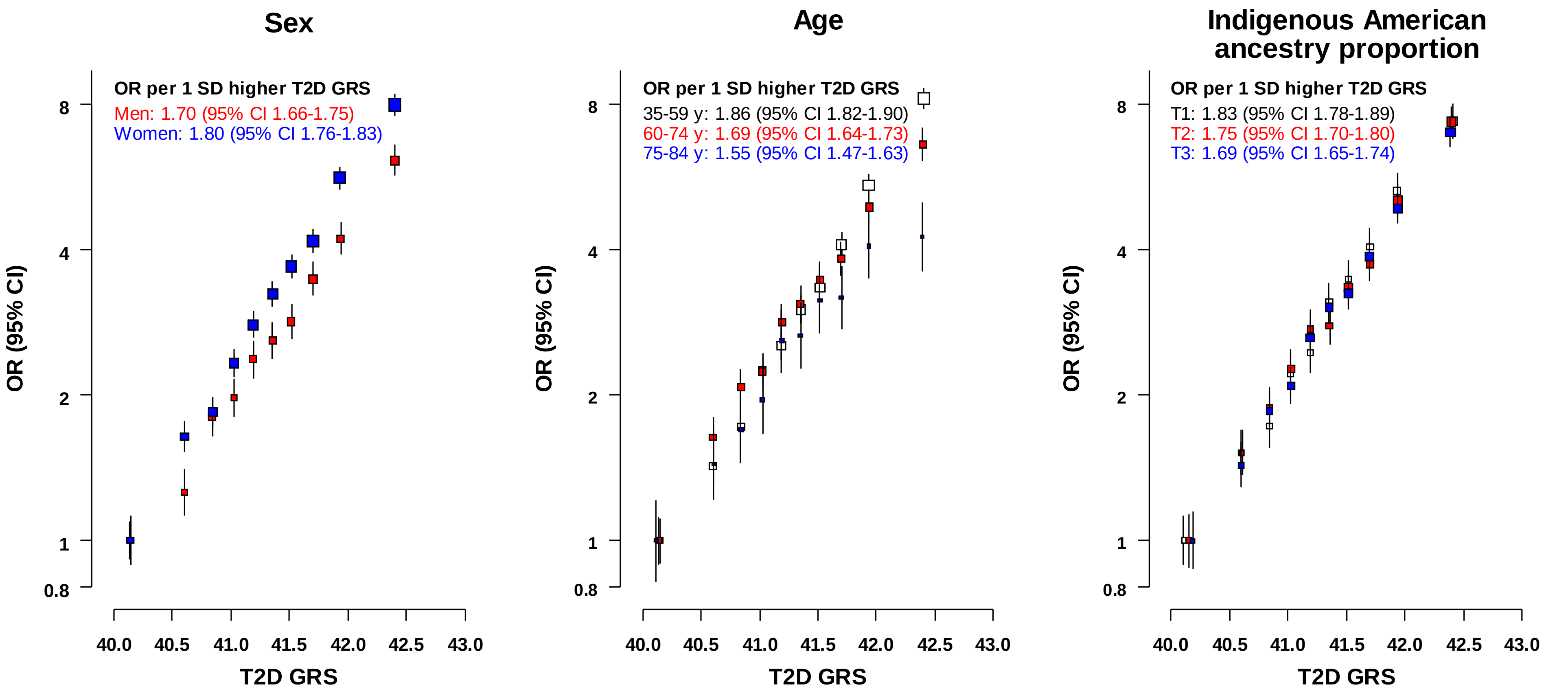

Observed type 2 diabetes (T2D) status refers to previously diagnosed or undiagnosed T2D at recruitment. The T2D genetic risk score (GRS) represents the sum across all SNPs of their effect allele count multiplied by the log odds ratio (OR) for the association of the SNP with T2D in the T2D Global Genomics Initiative multi-ancestry metaregression. T2D GRS SD: men=0.64 units; women=0.64 units; 35-59 y=0.64 units; 60-74 y=0.64 units; 75-84 y=0.63 units; Indigenous American ancestry proportion T1=0.67 units; Indigenous American ancestry proportion T2=0.64 units; Indigenous American ancestry proportion T3=0.60 units. T2D ORs are adjusted for age, sex and the first 7 principal components, where appropriate. The size of each square is proportional to the amount of data available. Error bars represent 95% confidence intervals (CI). T=third.

Supplementary Figure S4: Association of Hispanic type 2 diabetes GRS with observed type 2 diabetes status among 125,587 participants aged 35-84 years at recruitment

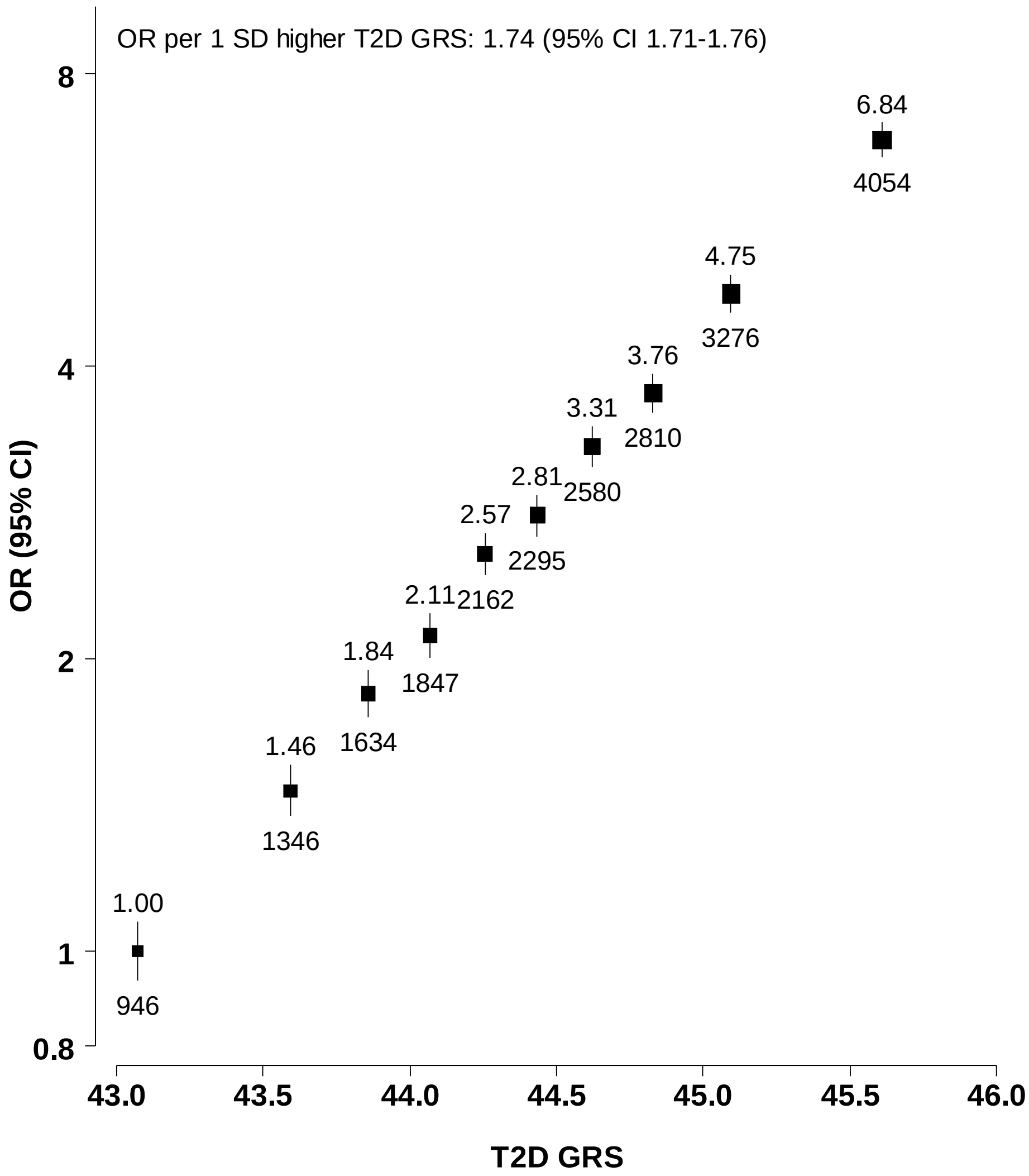

Observed type 2 diabetes (T2D) status refers to previously diagnosed or undiagnosed T2D at recruitment. The T2D genetic risk score (GRS) represents the sum across all SNPs of their effect allele count multiplied by the log odds ratio (OR) for the association of the SNP with T2D from Hispanic ancestry groups within the T2D Global Genomics Initiative. T2D GRS SD=0.72 units. T2D ORs are adjusted for age, sex and the first 7 principal components. The numbers above the squares are the ORs and the numbers below the squares are the number of participants with T2D in that group. The size of each square is proportional to the amount of data available. Error bars represent 95% confidence intervals (CI).

Supplementary Figure S5: Association of pathway-specific type 2 diabetes GRSs with type 2 diabetes status among 125,587 participants aged 35-84 years at recruitment

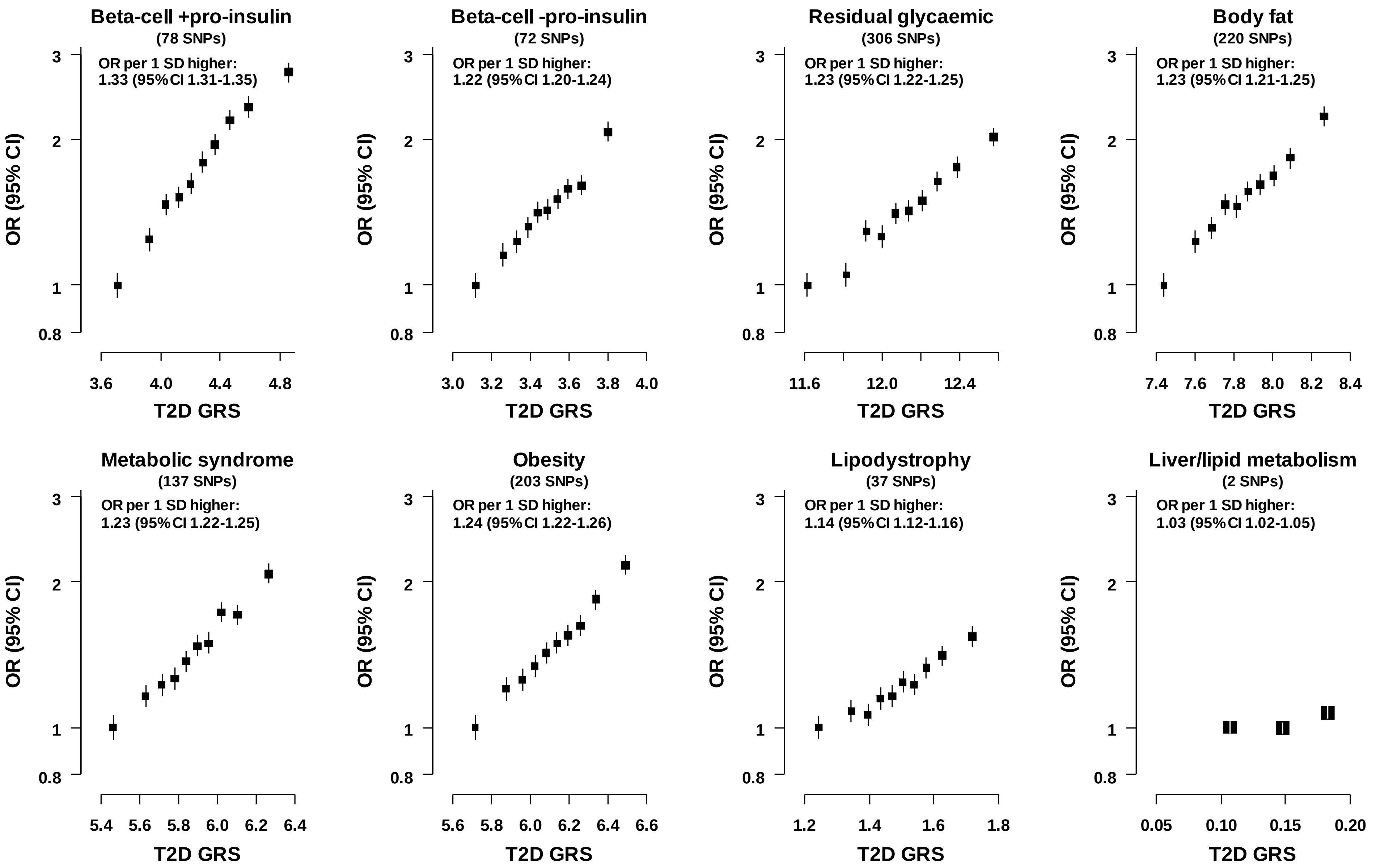

Type 2 diabetes (T2D) refers to previously diagnosed or undiagnosed T2D at recruitment. The T2D genetic risk score (GRS) represents the sum across all SNPs of their effect allele count multiplied by the log odds ratio (OR) for the association of the SNP with T2D in the T2D Global Genomics Initiative multi-ancestry metaregression. Beta-cell +pro-insulin GRS SD: 0.0.32 units; Beta-cell -pro-insulin GRS SD: 0.19 units; Residual glycaemic GRS SD: 0.27 units; Body fat GRS SD: 0.23 units; Metabolic syndrome GRS SD: 0.23 units; Obesity GRS SD: 0.22 units; Lipodystrophy GRS SD: 0.13 units; Liver/lipid metabolism GRS SD: 0.03 units. T2D ORs are adjusted for age, sex and the first 7 principal components. The size of each square is proportional to the amount of data available. Error bars represent 95% confidence intervals (CI).

Supplementary Figure S6: Associations of type 2 diabetes GRS with circulating metabolic biomarkers among 125,587 participants aged 35-84 years at recruitment

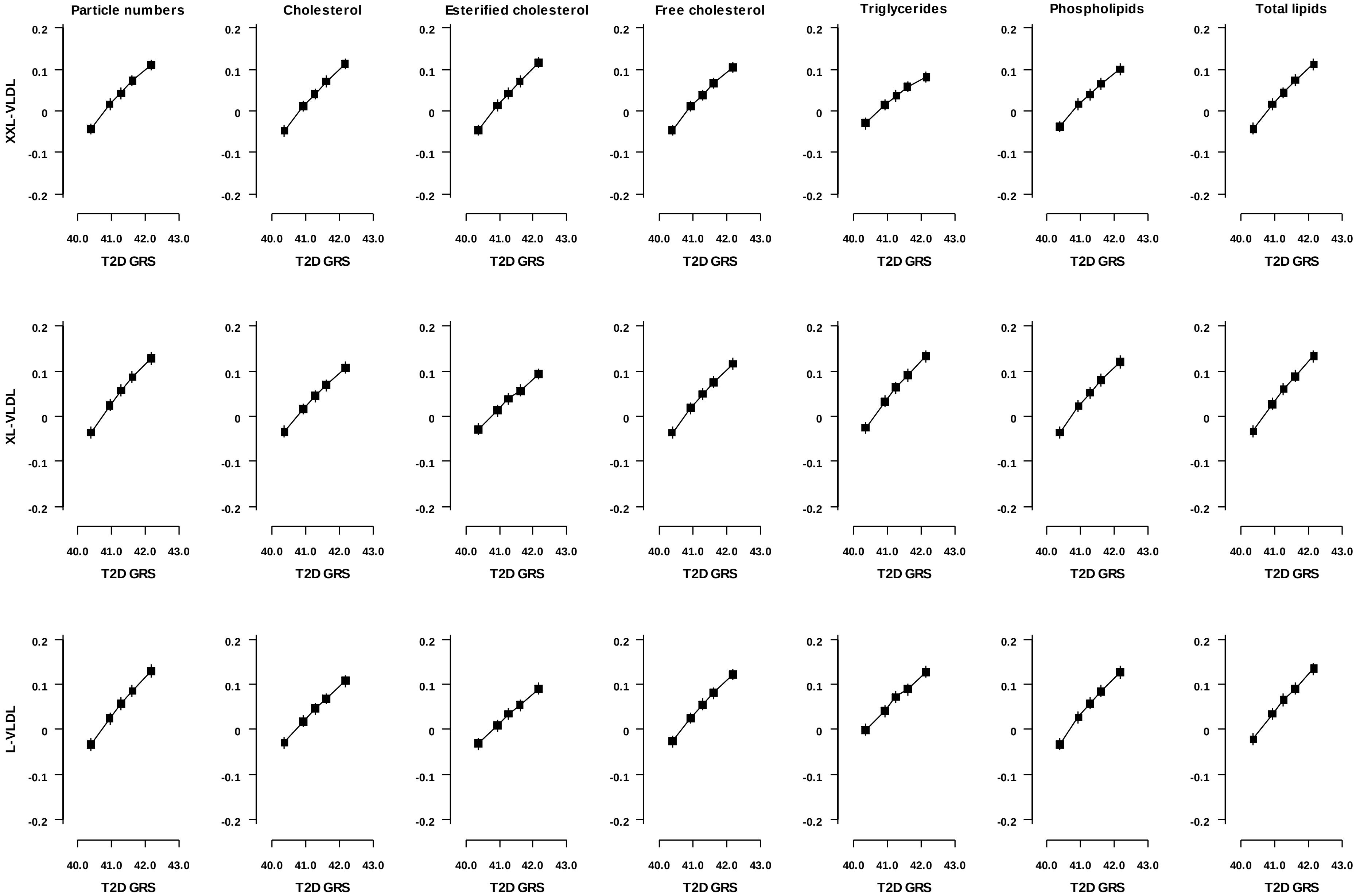

Points represent means (with 95% CI) of log−transformed (and then scaled to a mean of 0 and an SD of 1) metabolic biomarkers for each fifth of the type 2 diabetes (T2D) genetic risk score (GRS). Estimates are adjusted for age, sex, the first 7 genetic principal components and fasting time. FA=fatty acid; HDL=high-density lipoproteins; IDL=intermediate density lipoproteins; L=large; LDL=low-density lipoproteins; M=medium; S=small; VLDL=very low density lipoproteins; XL=very large; XXL=extremely large; XS=very small.

Supplementary Figure S6: Associations of type 2 diabetes GRS with circulating metabolic biomarkers among 125,587 participants aged 35-84 years at recruitment

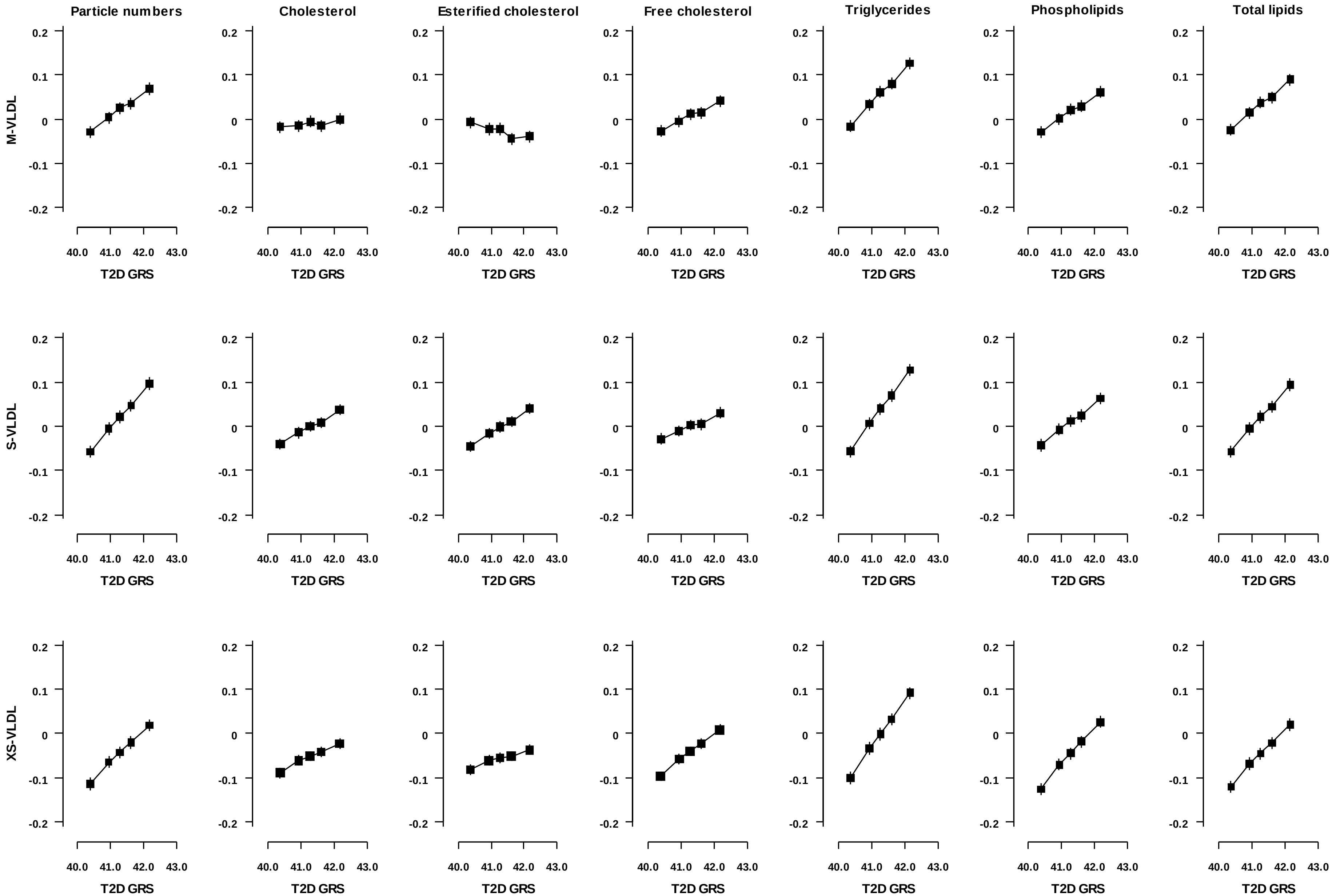

Points represent means (with 95% CI) of log−transformed (and then scaled to a mean of 0 and an SD of 1) metabolic biomarkers for each fifth of the type 2 diabetes (T2D) genetic risk score (GRS). Estimates are adjusted for age, sex, the first 7 genetic principal components and fasting time. FA=fatty acid; HDL=high-density lipoproteins; IDL=intermediate density lipoproteins; L=large; LDL=low-density lipoproteins; M=medium; S=small; VLDL=very low density lipoproteins; XL=very large; XXL=extremely large; XS=very small.

Supplementary Figure S6: Associations of type 2 diabetes GRS with circulating metabolic biomarkers among 125,587 participants aged 35-84 years at recruitment

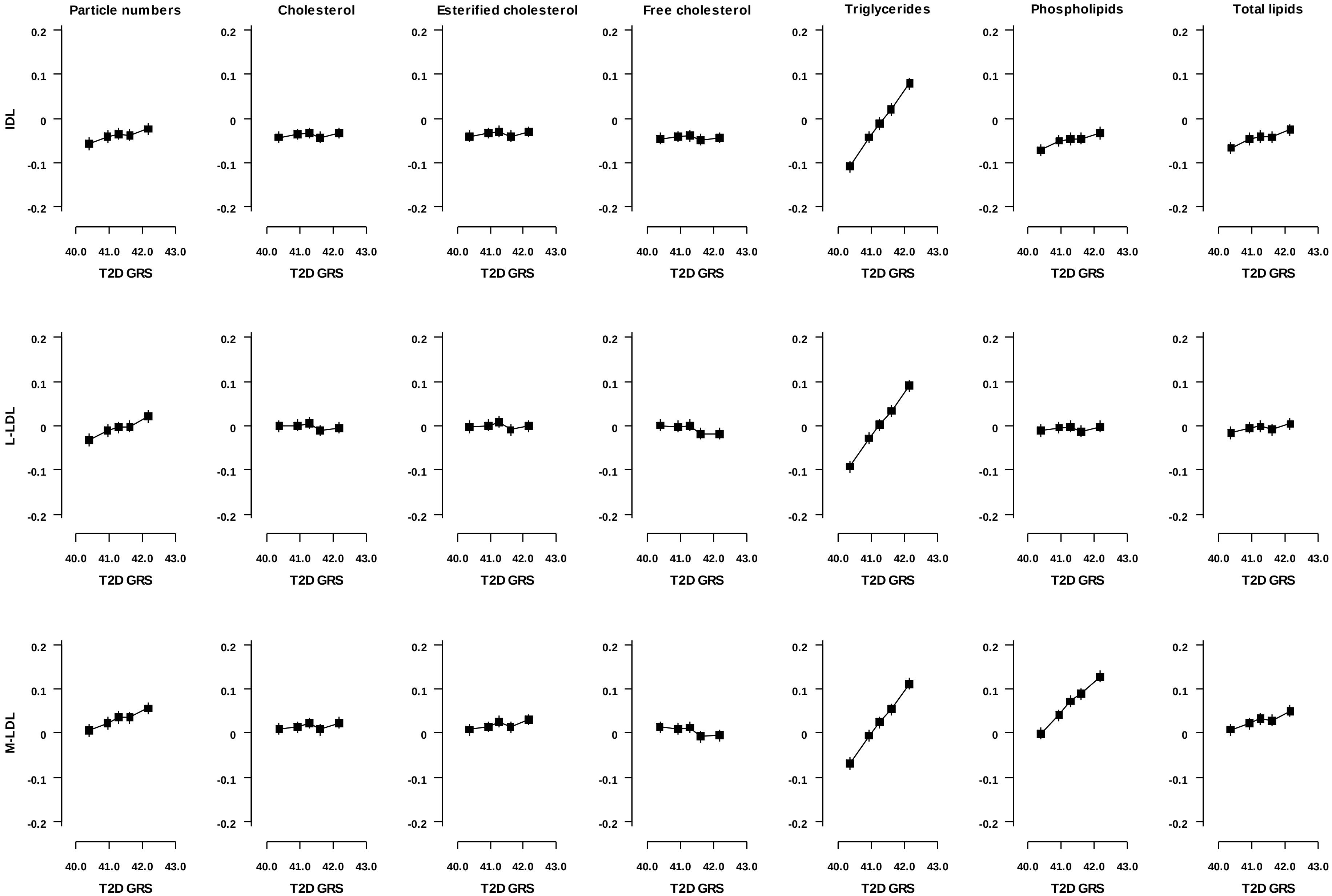

Points represent means (with 95% CI) of log−transformed (and then scaled to a mean of 0 and an SD of 1) metabolic biomarkers for each fifth of the type 2 diabetes (T2D) genetic risk score (GRS). Estimates are adjusted for age, sex, the first 7 genetic principal components and fasting time. FA=fatty acid; HDL=high-density lipoproteins; IDL=intermediate density lipoproteins; L=large; LDL=low-density lipoproteins; M=medium; S=small; VLDL=very low density lipoproteins; XL=very large; XXL=extremely large; XS=very small.

Supplementary Figure S6: Associations of type 2 diabetes GRS with circulating metabolic biomarkers among 125,587 participants aged 35-84 years at recruitment

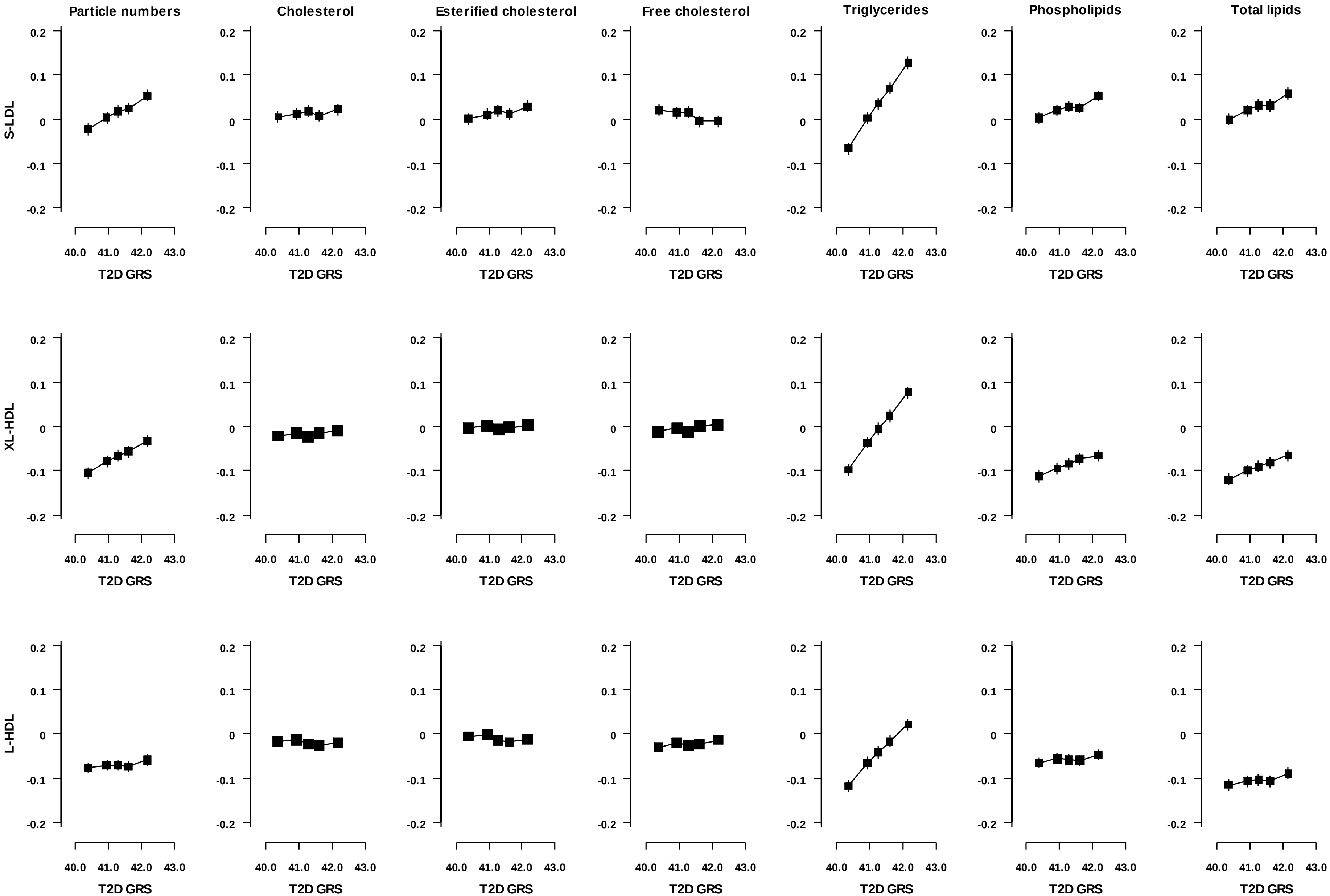

Points represent means (with 95% CI) of log−transformed (and then scaled to a mean of 0 and an SD of 1) metabolic biomarkers for each fifth of the type 2 diabetes (T2D) genetic risk score (GRS). Estimates are adjusted for age, sex, the first 7 genetic principal components and fasting time. FA=fatty acid; HDL=high-density lipoproteins; IDL=intermediate density lipoproteins; L=large; LDL=low-density lipoproteins; M=medium; S=small; VLDL=very low density lipoproteins; XL=very large; XXL=extremely large; XS=very small.

Supplementary Figure S6: Associations of type 2 diabetes GRS with circulating metabolic biomarkers among 125,587 participants aged 35-84 years at recruitment

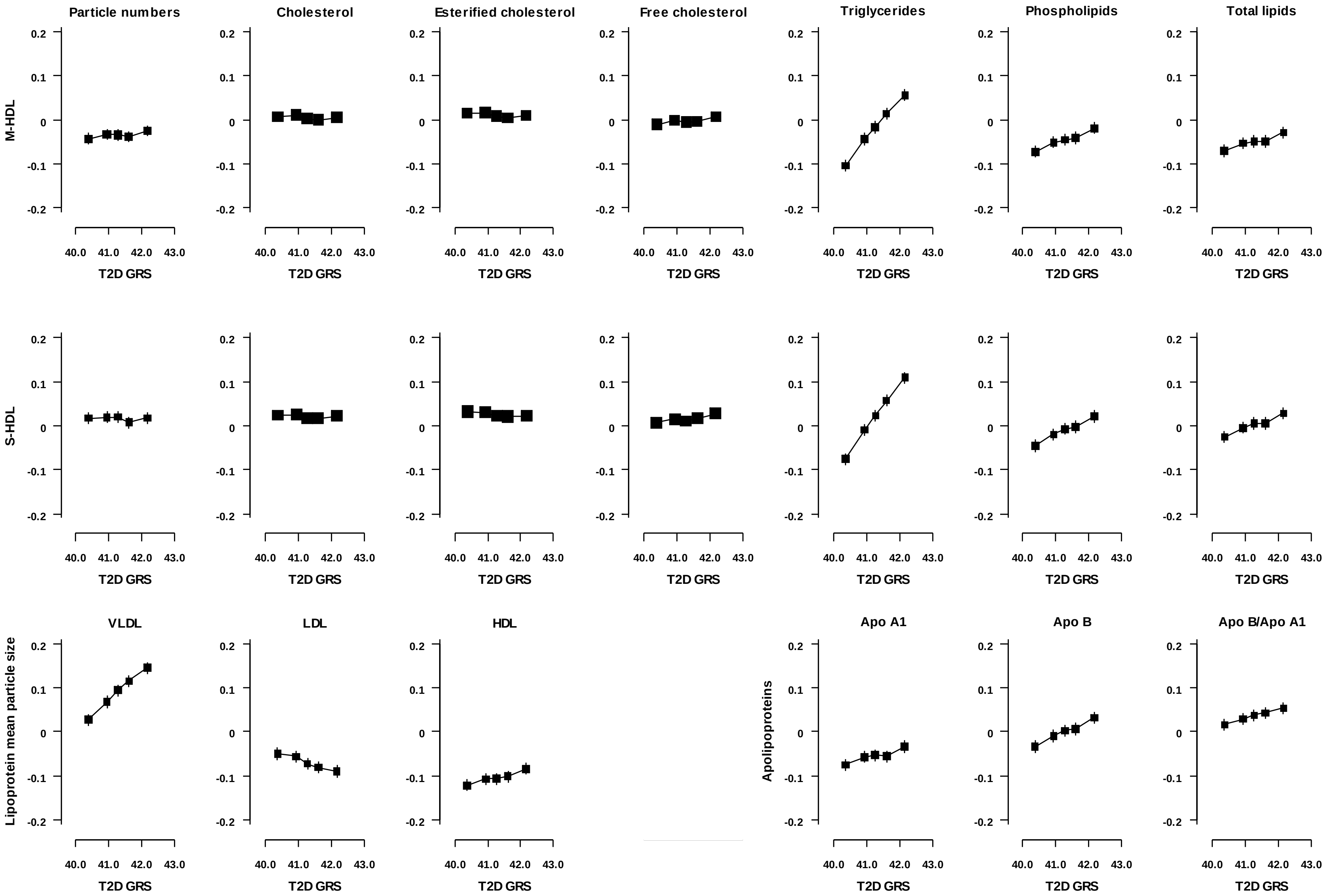

Points represent means (with 95% CI) of log−transformed (and then scaled to a mean of 0 and an SD of 1) metabolic biomarkers for each fifth of the type 2 diabetes (T2D) genetic risk score (GRS). Estimates are adjusted for age, sex, the first 7 genetic principal components and fasting time. FA=fatty acid; HDL=high-density lipoproteins; IDL=intermediate density lipoproteins; L=large; LDL=low-density lipoproteins; M=medium; S=small; VLDL=very low density lipoproteins; XL=very large; XXL=extremely large; XS=very small.

Supplementary Figure S6: Associations of type 2 diabetes GRS with circulating metabolic biomarkers among 125,587 participants aged 35-84 years at recruitment

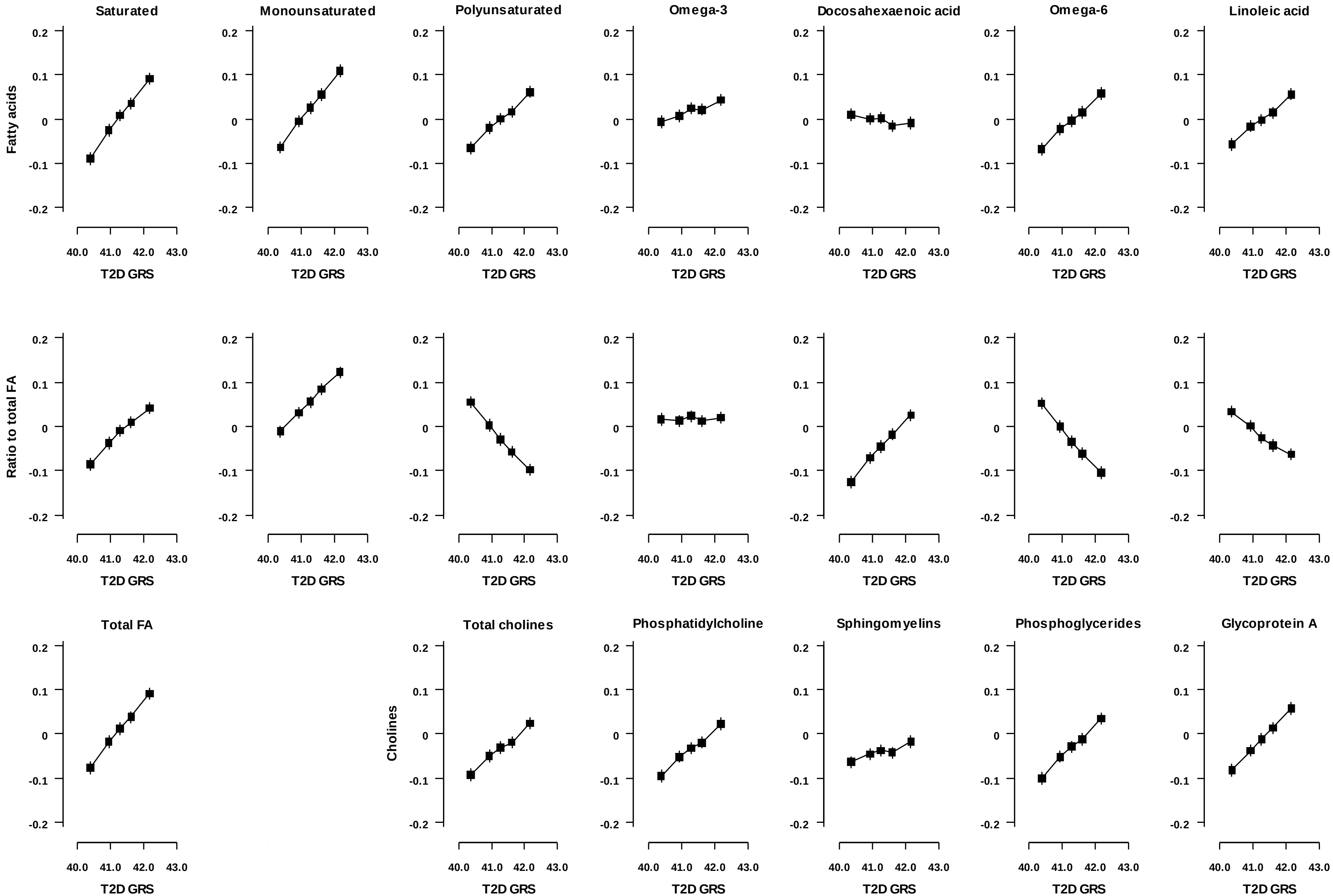

Points represent means (with 95% CI) of log−transformed (and then scaled to a mean of 0 and an SD of 1) metabolic biomarkers for each fifth of the type 2 diabetes (T2D) genetic risk score (GRS). Estimates are adjusted for age, sex, the first 7 genetic principal components and fasting time. FA=fatty acid; HDL=high-density lipoproteins; IDL=intermediate density lipoproteins; L=large; LDL=low-density lipoproteins; M=medium; S=small; VLDL=very low density lipoproteins; XL=very large; XXL=extremely large; XS=very small..

Supplementary Figure S6: Associations of type 2 diabetes GRS with circulating metabolic biomarkers among 125,587 participants aged 35-84 years at recruitment

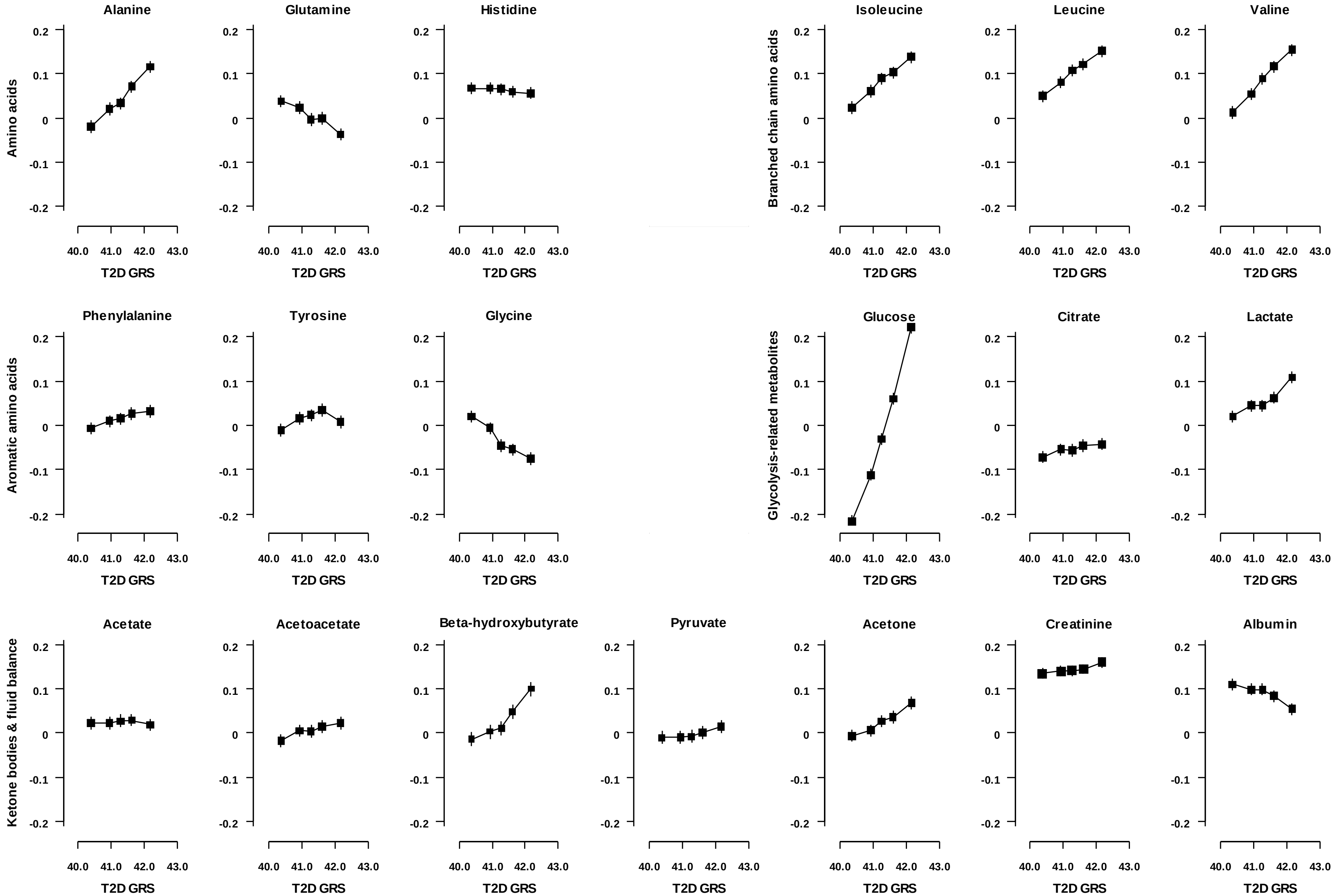

Points represent means (with 95% CI) of log−transformed (and then scaled to a mean of 0 and an SD of 1) metabolic biomarkers for each fifth of the type 2 diabetes (T2D) genetic risk score (GRS). Estimates are adjusted for age, sex, the first 7 genetic principal components and fasting time. FA=fatty acid; HDL=high-density lipoproteins; IDL=intermediate density lipoproteins; L=large; LDL=low-density lipoproteins; M=medium; S=small; VLDL=very low density lipoproteins; XL=very large; XXL=extremely large; XS=very small.

Supplementary Figure S7: Associations of observed type 2 diabetes status at recruitment with circulating metabolic biomarkers among 134,448 participants aged 35−84 years at recruitment

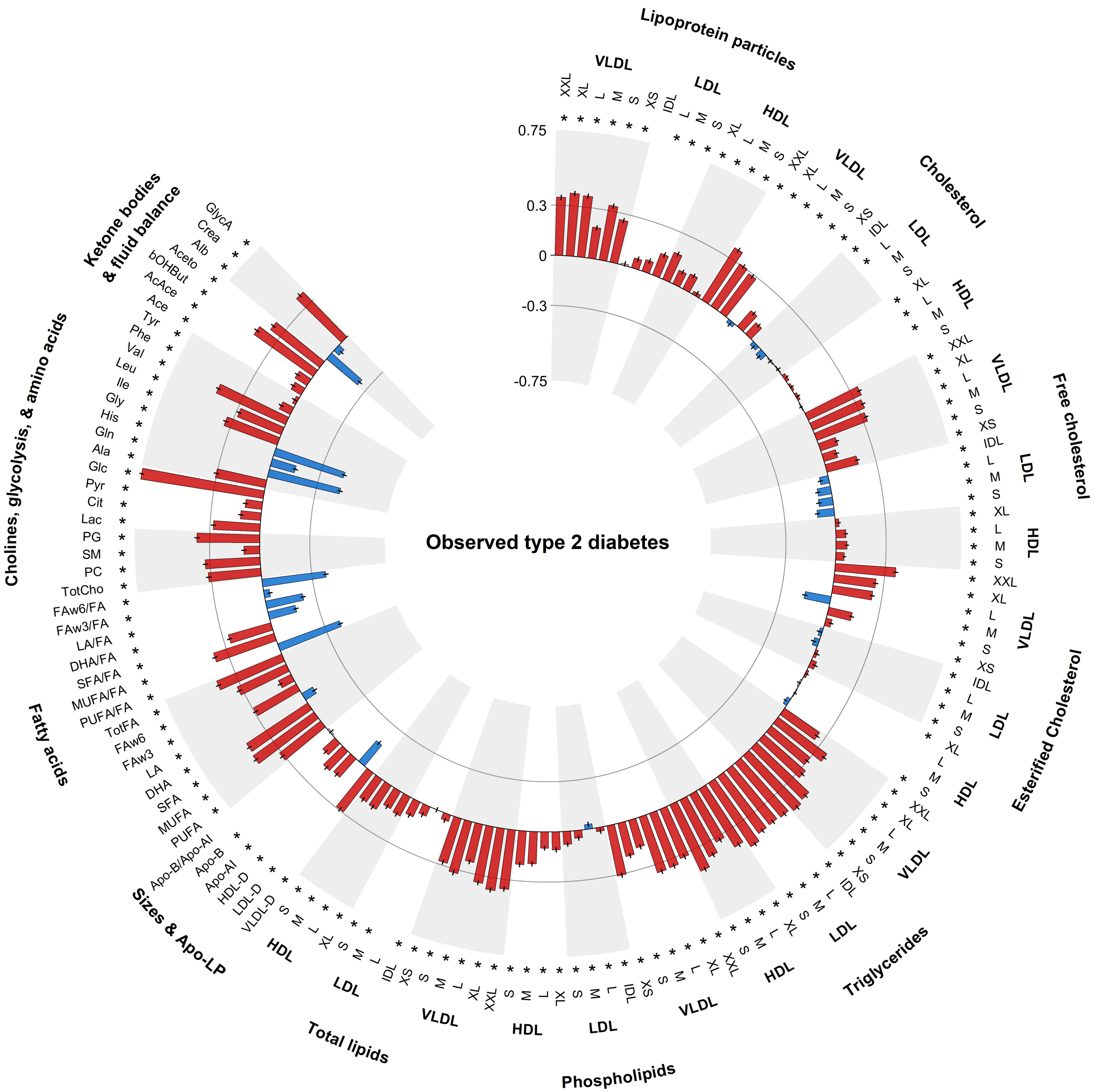

Observed type 2 diabetes refers to previously diagnosed or undiagnosed type 2 diabetes. Difference (in SD) units of each log−NMR biomarker are adjusted for age, sex, district, educational level, smoking status, alcohol drinking, height, weight, waist circumference, hip circumference and fasting duration. *False discovery rate controlled p<0.05. Regression models based on 92,993 participants for bOHBut, 129,934 participants for citrate, 130,061 participants for creatinine, 129,238 participants for glutamine, 126,843 participants for pyruvate, 130,050 participants for valine. AcAce=acetoacetate; Ace=acetate; Aceto=acetone; Ala=alanine; Alb=albumin; Apo−A1=apolipoprotein A1; Apo−B=apolipoprotein B; bOHBut=beta−hydroxybutyrate; Cit=citrate; Crea=creatinine; DHA=docosahexaenoic acid; FA=fatty acids; FAw3=omega−3; fatty acids; FAw6=omega−6 fatty acids; Glc=glucose; Gln=glutamine; Gly=glycine; Glyc−A=glycoprotein acetyls; HDL=high density lipoproteins; HDL−D=high density lipoprotein particle diameter; His=histidine; IDL=intermediate density lipoproteins; Ile=isoleucine; L=large; LA=linoleic acid; Lac=lactate; LDL=low density lipoproteins; LDL−D=low density lipoprotein particle diameter; Leu=leucine; LP=lipoprotein; M=medium; MUFA=monounsaturated fatty acids; PC=phosphatidylcholines; PG=phosphoglycerides; Phe=phenylalanine; PUFA=polyunsaturated fatty acids; Pyr=pyruvate; S=small; SFA=saturated fatty acids; SM=sphingomyelins; TotFA=total fatty acids; TotCho=total cholines; Tyr=tyrosine; Val=valine; VLDL=very low density lipoproteins; VLDL−D=very low density lipoprotein particle diameter; XL=very large; XS=very small; XXL=extremely large.

Supplementary Figure S8: Associations of observed previously diagnosed and undiagnosed type 2 diabetes status at recruitment with circulating metabolic biomarkers among 134,448 participants aged 35−84 years at recruitment

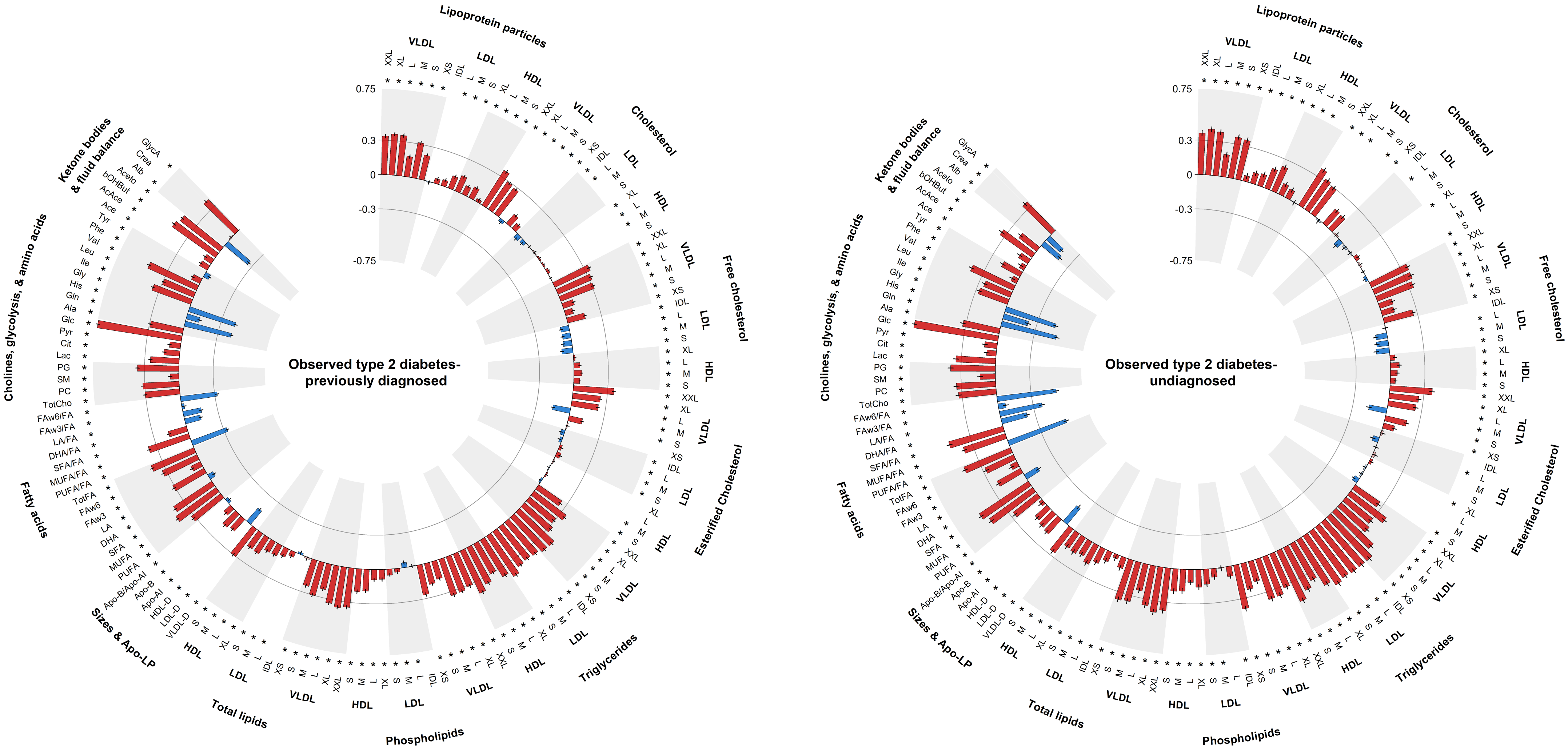

Difference (in SD) units of each log−NMR biomarker are adjusted for age, sex, district, educational level, smoking status, alcohol drinking, height, weight, waist circumference, hip circumference and fasting duration versus participants with no diabetes. *False discovery rate controlled p<0.05. Regression models based on 92,993 participants for bOHBut, 129,934 participants for citrate, 130,061 participants for creatinine, 129,238 participants for glutamine, 126,843 participants for pyruvate, 130,050 participants for valine. AcAce=acetoacetate; Ace=acetate; Aceto=acetone; Ala=alanine; Alb=albumin; Apo−A1=apolipoprotein A1; Apo−B=apolipoprotein B; bOHBut=beta−hydroxybutyrate; Cit=citrate; Crea=creatinine; DHA=docosahexaenoic acid; FA=fatty acids; FAw3=omega−3 fatty acids; FAw6=omega−6 fatty acids; Glc=glucose; Gln=glutamine; Gly=glycine; Glyc−A=glycoprotein acetyls; HDL=high density lipoproteins; HDL−D=high density lipoprotein particle diameter; His=histidine; IDL=intermediate density lipoproteins; Ile=isoleucine; L=large; LA=linoleic acid; Lac=lactate; LDL=low density lipoproteins; LDL−D=low density lipoprotein particle diameter; Leu=leucine; LP=lipoprotein; M=medium; MUFA=monounsaturated fatty acids; PC=phosphatidylcholines; PG=phosphoglycerides; Phe=phenylalanine; PUFA=polyunsaturated fatty acids; Pyr=pyruvate; S=small; SFA=saturated fatty acids; SM=sphingomyelins; TotFA=total fatty acids; TotCho=total cholines; Tyr=tyrosine; Val=valine; VLDL=very low density lipoproteins; VLDL−D=very low density lipoprotein particle diameter; XL=very large; XS=very small; XXL=extremely large.

Supplementary Figure S9: Associations of pathway−specific type 2 diabetes GRSs with circulating metabolic biomarkers among 125,587 participants aged 35−84 years at recruitment, before and after adjustment for genetically−predicted measures of adiposity

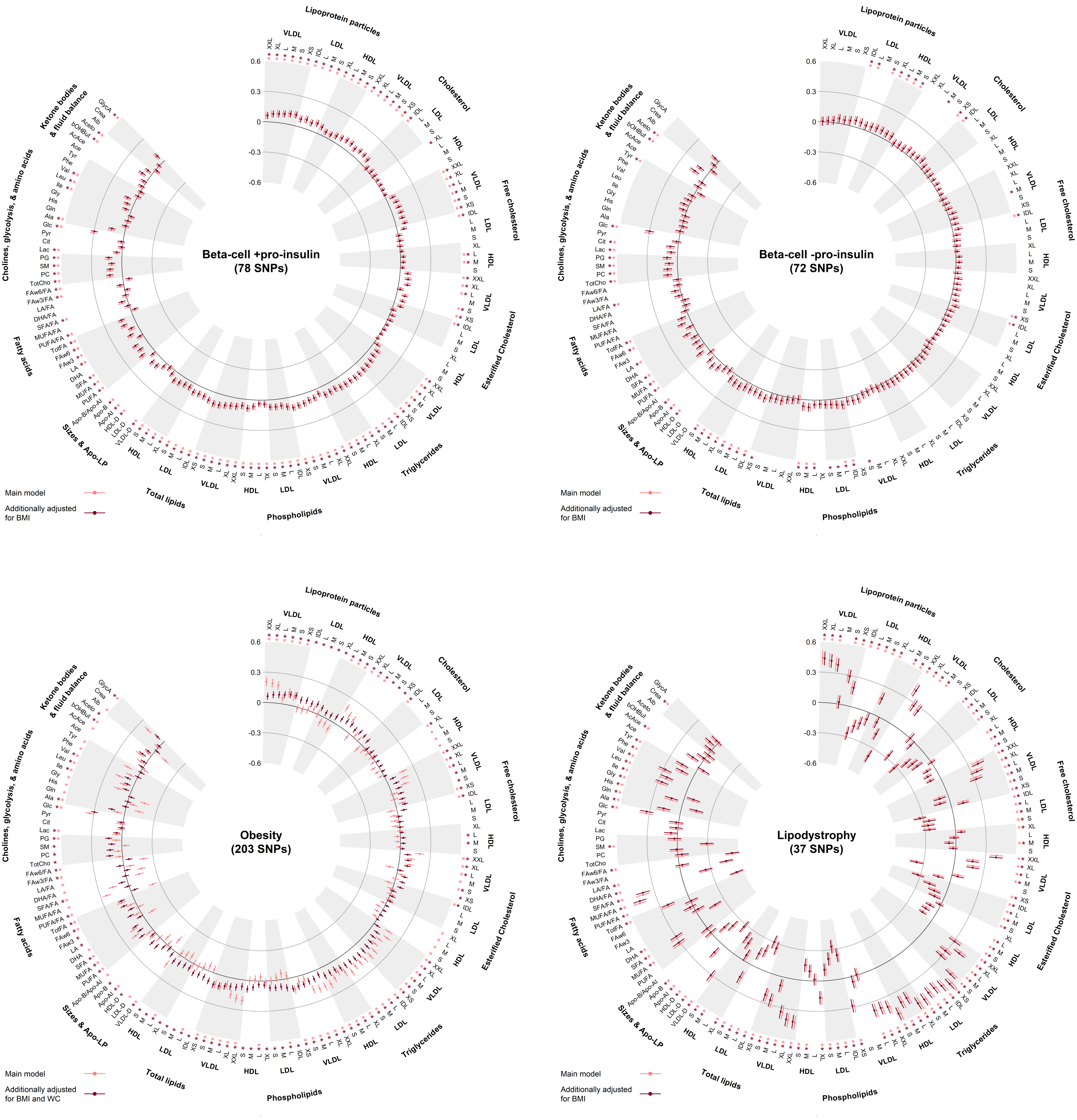

Difference (in SD units) of each log−NMR biomarker are per trebling in the genetically−predicted odds of type 2 diabetes and are adjusted for age, sex, the first 7 genetic principal components, fasting time and genetically−predicted measures of adiposity. *False discovery rate controlled p<0.05. Findings based on 86,574 participants for bOHBut, 121,391 participants for citrate, 121,368 participants for creatinine, 120,576 participants for glutamine, 118,255 participants for pyruvate, 121,374 participants for valine. AcAce=acetoacetate; Ace=acetate; Aceto=acetone; Ala=alanine; Alb=albumin; Apo−A1=apolipoprotein A1; Apo−B=apolipoprotein B; BMI=body mass index; bOHBut=beta−hydroxybutyrate; Cit=citrate; Crea=creatinine; DHA=docosahexaenoic acid; FA=fatty acids; FAw3=omega−3; fatty acids; FAw6=omega−6 fatty acids; Glc=glucose; Gln=glutamine; Gly=glycine; Glyc−A=glycoprotein acetyls; GRS=genetic risk score; HDL=high density lipoproteins; HDL−D=high density lipoprotein particle diameter; His=histidine; IDL=intermediate density lipoproteins; Ile=isoleucine; L=large; LA=linoleic acid; Lac=lactate; LDL=low density lipoproteins; LDL−D=low density lipoprotein particle diameter; Leu=leucine; LP=lipoprotein; M=medium; MUFA=monounsaturated fatty acids; PC=phosphatidylcholines; PG=phosphoglycerides; Phe=phenylalanine; PUFA=polyunsaturated fatty acids; Pyr=pyruvate; S=small; SFA=saturated fatty acids; SM=sphingomyelins; TotFA=total fatty acids; TotCho=total cholines; Tyr=tyrosine; Val=valine; VLDL=very low density lipoproteins; VLDL−D=very low density lipoprotein particle diameter; WC=waist circumference; XL=very large; XS=very small; XXL=extremely large.

Supplementary Figure S10: Associations of genetically-predicted liability to type 2 diabetes with circulating metabolic biomarkers among 125,587 participants aged 35-84 years at recruitment, by age, sex, district and Indigenous American ancestry proportion

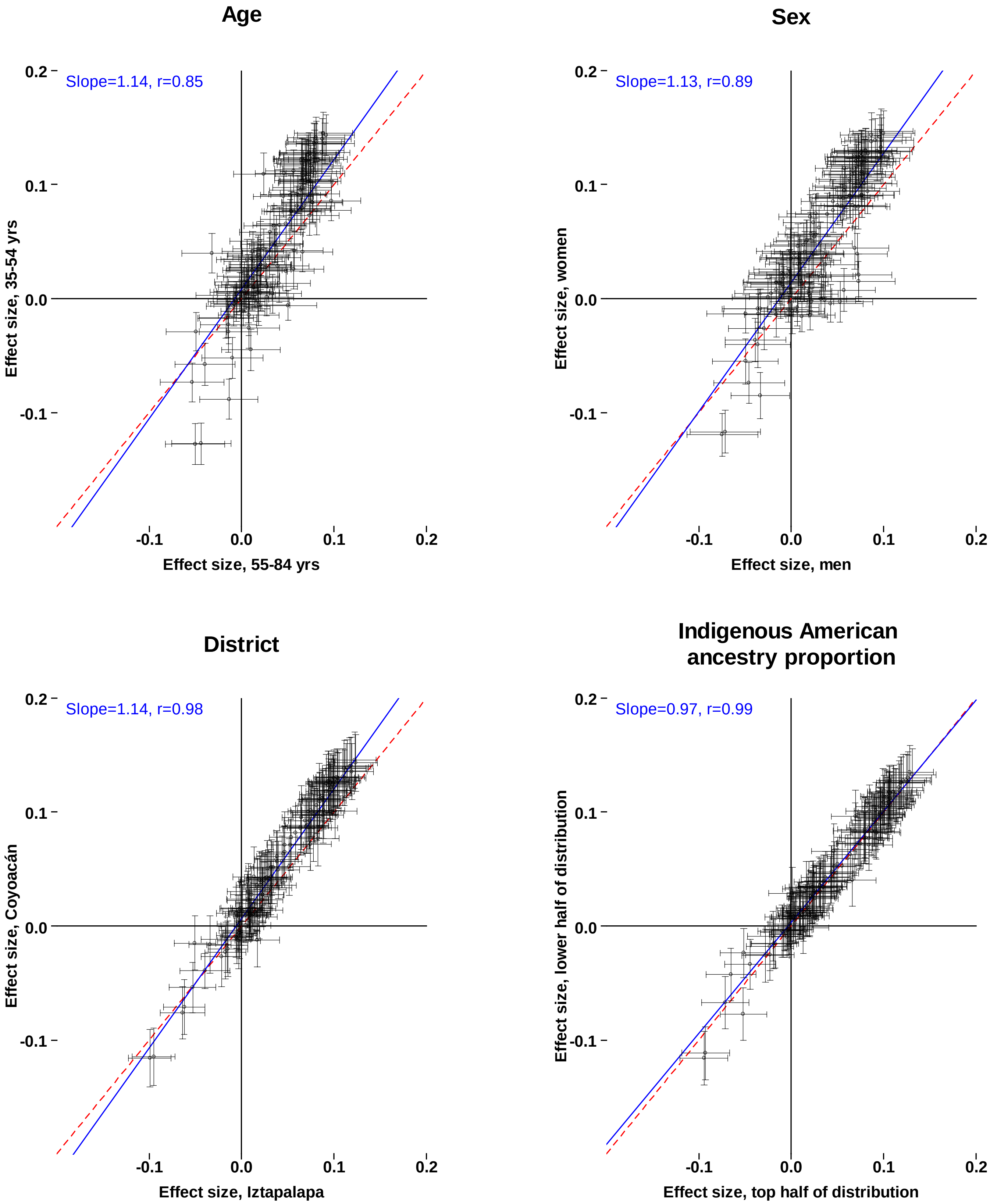

Difference (in SD units) of each log-NMR biomarker per trebling in the genetically-predicted odds of type 2 diabetes, adjusted for age, the first 7 genetic principal components and fasting time. Red dashed line is line of equality. Blue line is line of best fit from a linear regression. Findings based on 86,574 participants for beta-hydroxybutyrate, 121,391 participants for citrate, 121,368 participants for creatinine, 120,576 participants for glutamine, 118,255 participants for pyruvate, 121,374 participants for valine.

Supplementary Figure S11: Associations of pathway-specific type 2 diabetes GRSs with circulating metabolic biomarkers among 125,587 participants aged 35-84 years at recruitment, by age

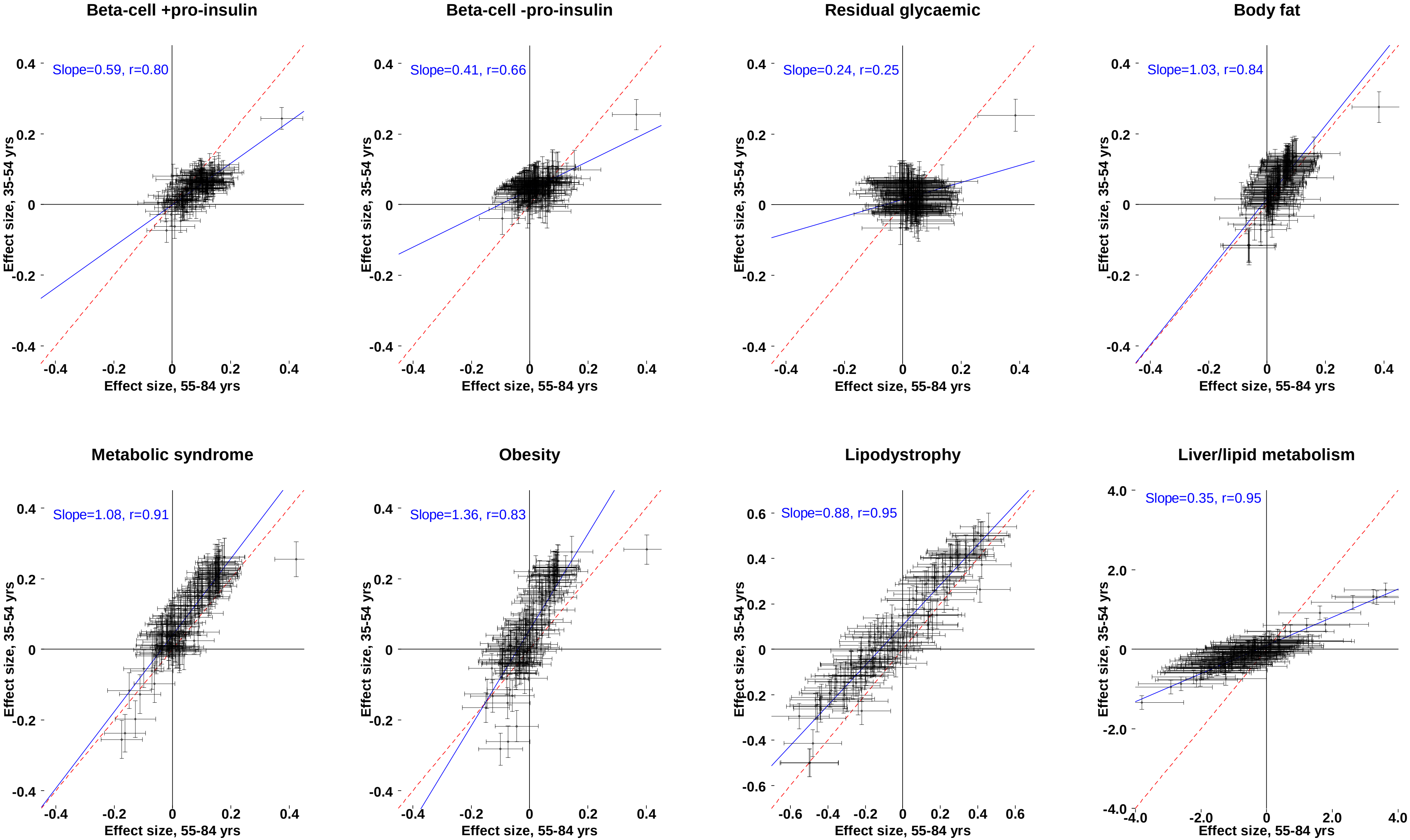

Difference (in SD units) of each log-NMR biomarker are per trebling in the genetically-predicted odds of type 2 diabetes and are adjusted for age, sex, the first 7 genetic principal components and fasting time. Red dashed line is line of equality. Blue line is line of best fit from a linear regression. Findings based on 86,574 participants for beta-hydroxybutyrate, 121,391 participants for citrate, 121,368 participants for creatinine, 120,576 participants for glutamine, 118,255 participants for pyruvate, 121,374 participants for valine.

Supplementary Figure S12: Associations of pathway-specific type 2 diabetes GRSs with circulating metabolic biomarkers among 125,587 participants aged 35-84 years at recruitment, by sex

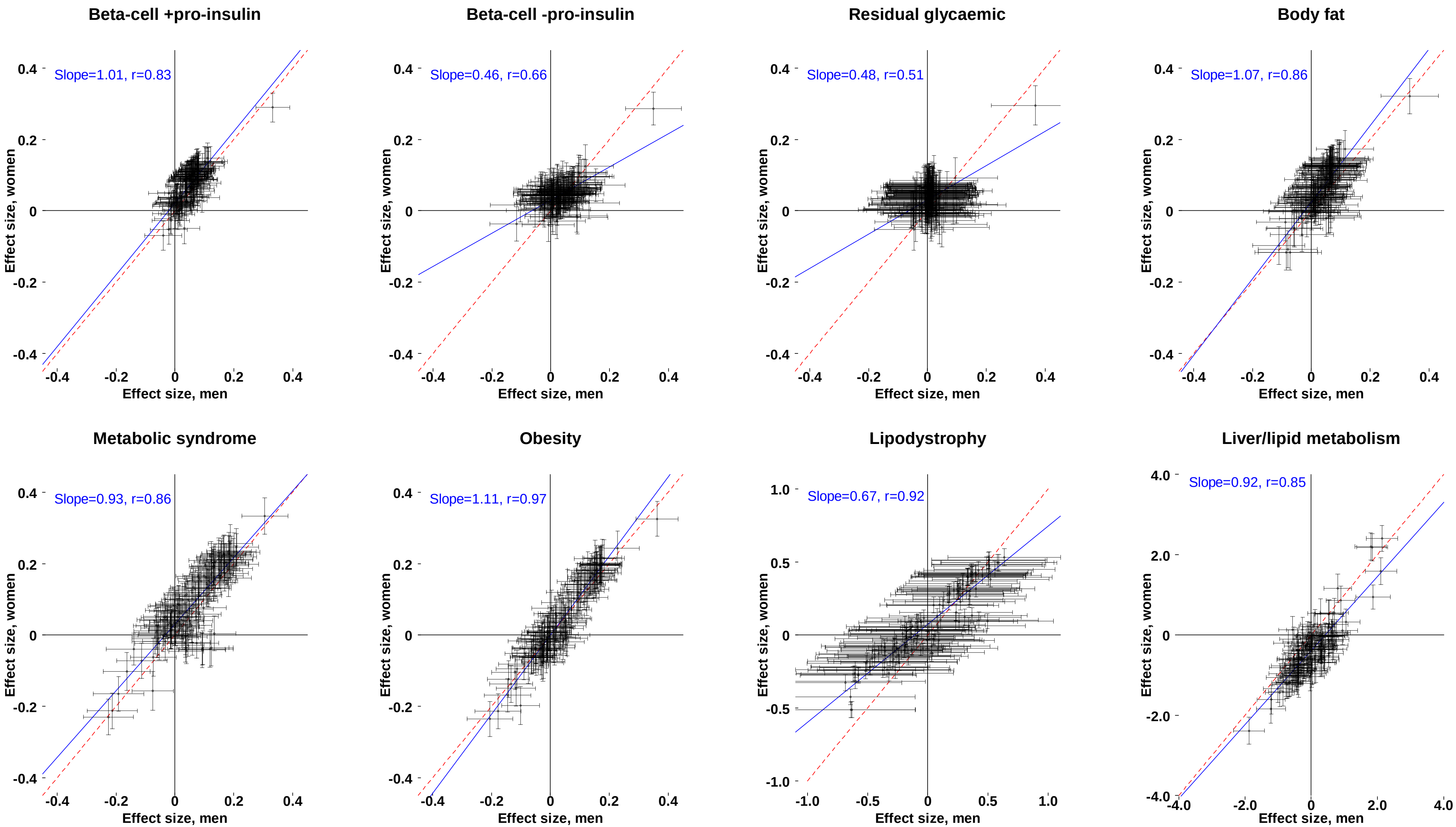

Difference (in SD units) of each log-NMR biomarker are per trebling in the genetically-predicted odds of type 2 diabetes and are adjusted for age, the first 7 genetic principal components and fasting time. Red dashed line is line of equality. Blue line is line of best fit from a linear regression. Findings based on 86,574 participants for beta-hydroxybutyrate, 121,391 participants for citrate, 121,368 participants for creatinine, 120,576 participants for glutamine, 118,255 participants for pyruvate, 121,374 participants for valine.

Supplementary Figure S13: Associations of pathway-specific type 2 diabetes GRSs with circulating metabolic biomarkers among 125,587 participants aged 35-84 years at recruitment, by district

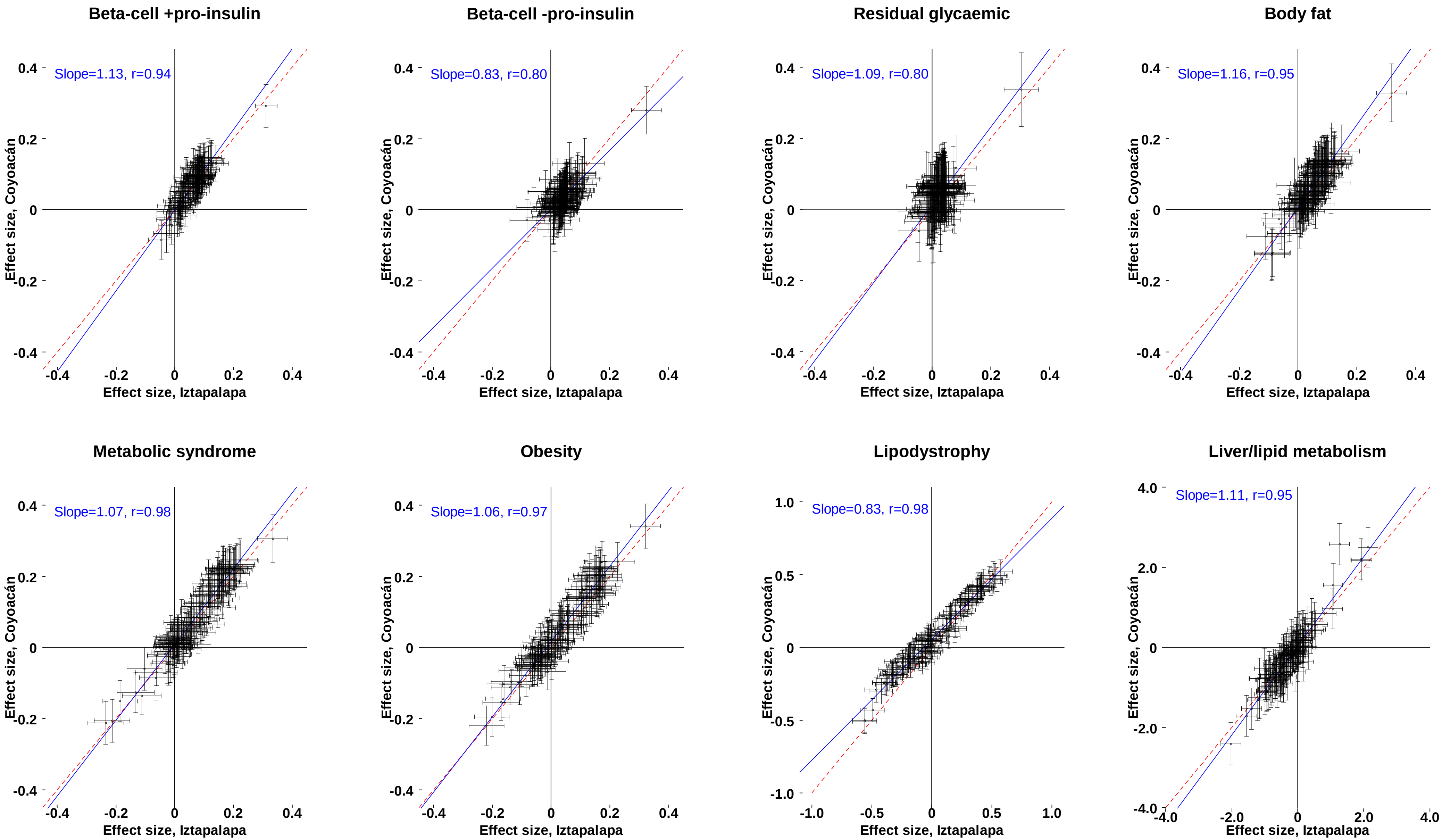

Difference (in SD units) of each log-NMR biomarker are per trebling in the genetically-predicted odds of type 2 diabetes and are adjusted for age, sex, the first 7 genetic principal components and fasting time. Red dashed line is line of equality. Blue line is line of best fit from a linear regression. Findings based on 86,574 participants for beta-hydroxybutyrate, 121,391 participants for citrate, 121,368 participants for creatinine, 120,576 participants for glutamine, 118,255 participants for pyruvate, 121,374 participants for valine.

Supplementary Figure S14: Associations of pathway-specific type 2 diabetes GRSs with circulating metabolic biomarkers among 125,587 participants aged 35-84 years at recruitment, by Indigenous American ancestry proportion

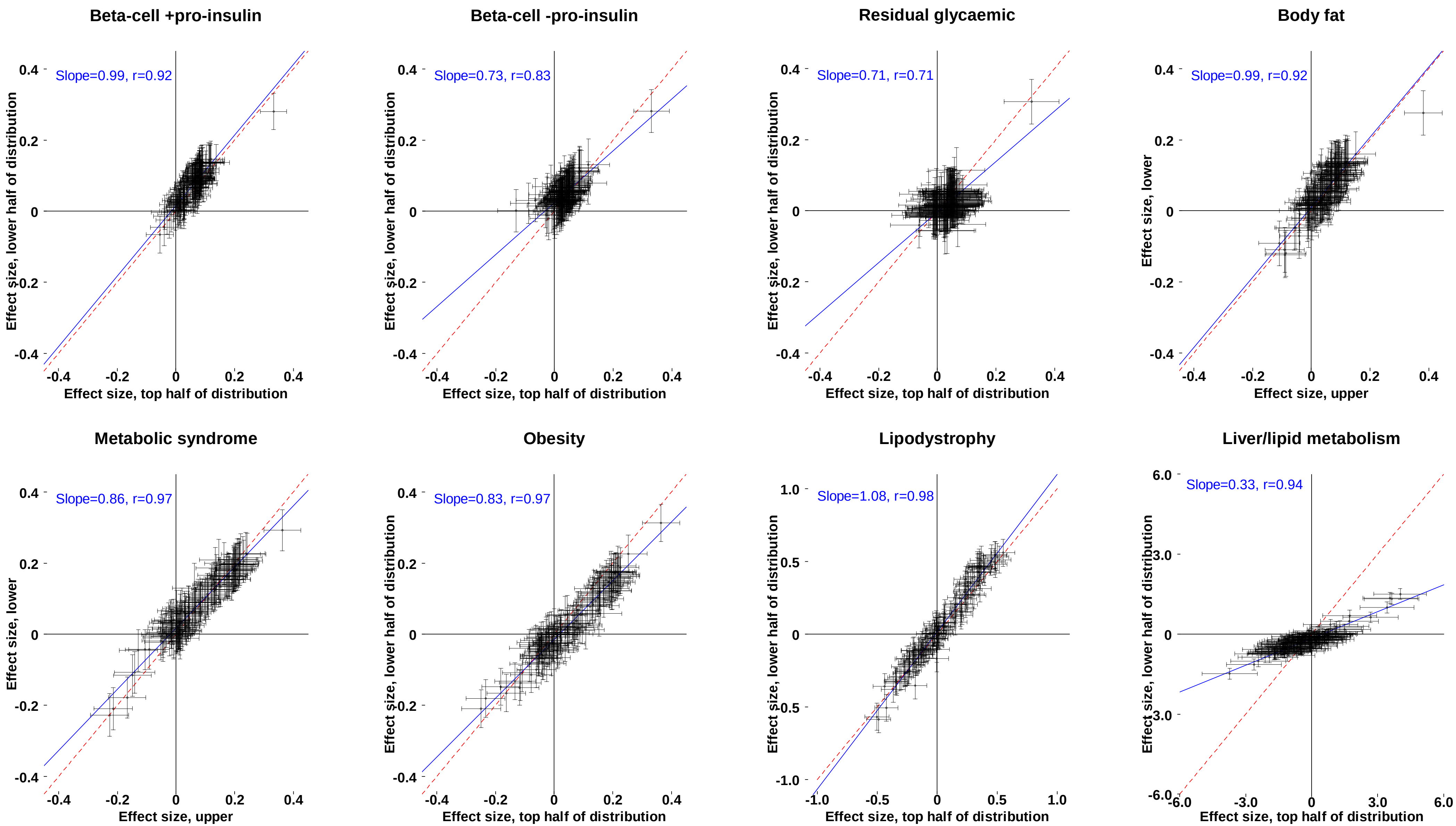

Difference (in SD units) of each log-NMR biomarker are per trebling in the genetically-predicted odds of type 2 diabetes and are adjusted for age, sex, the first 7 genetic principal components and fasting time. Red dashed line is line of equality. Blue line is line of best fit from a linear regression. Findings based on 86,574 participants for beta-hydroxybutyrate, 121,391 participants for citrate, 121,368 participants for creatinine, 120,576 participants for glutamine, 118,255 participants for pyruvate, 121,374 participants for valine.

Supplementary Figure S15: Associations of Hispanic type 2 diabetes GRS with circulating metabolic biomarkers among 125,587 participants aged 35−84 years at recruitment

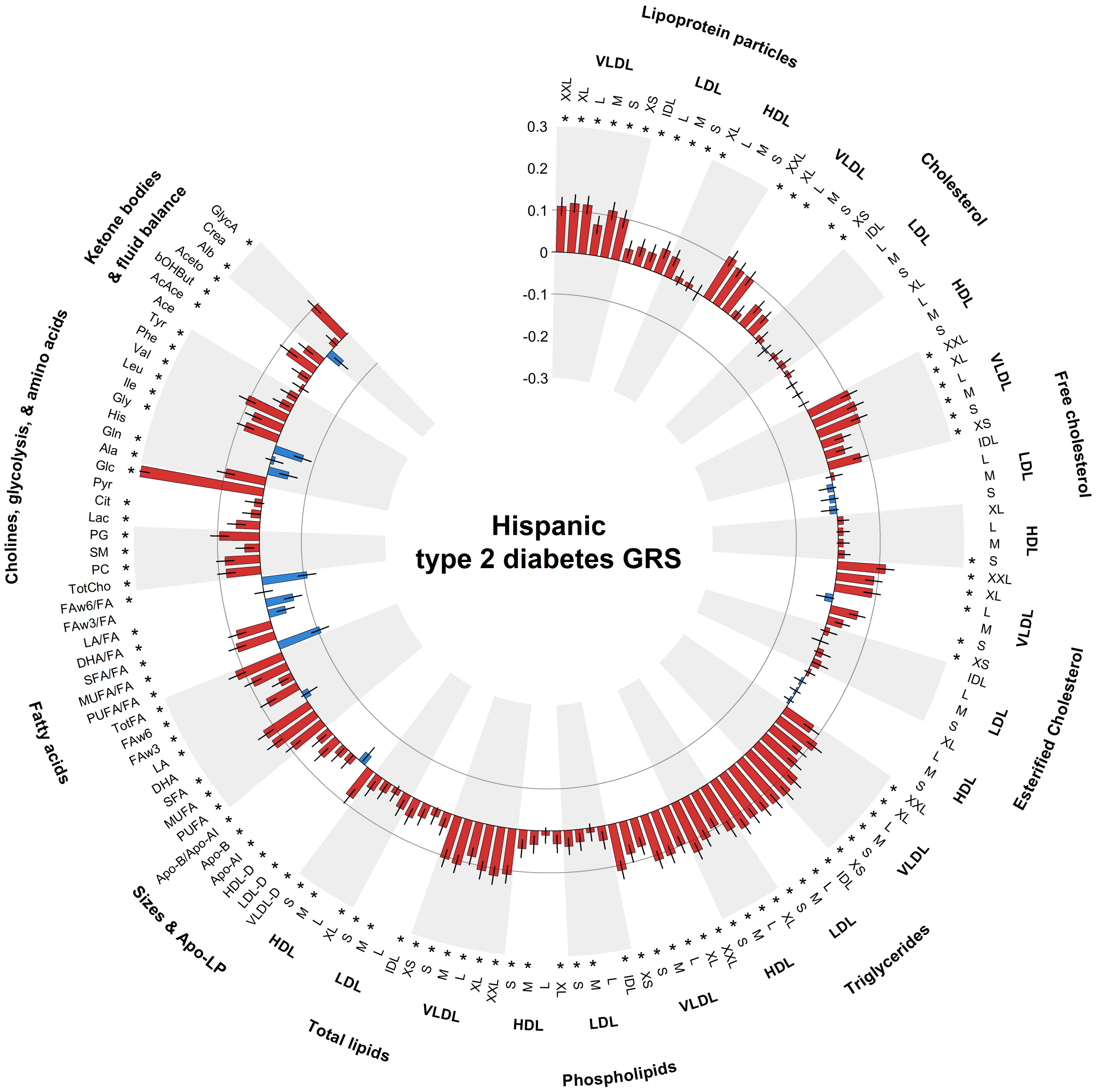

Difference (in SD) units of each log−NMR biomarker are per trebling in the genetically−predicted odds of type 2 diabetes and are adjusted for age, sex, the first 7 genetic principal components and fasting time. *False discovery rate controlled p<0.05. Findings based on 86,574 participants for bOHBut, 121,391 participants for citrate, 121,368 participants for creatinine, 120,576 participants for glutamine, 118,255 participants for pyruvate, 121,374 participants for valine. AcAce=acetoacetate; Ace=acetate; Aceto=acetone; Ala=alanine; Alb=albumin; Apo−A1=apolipoprotein A1; Apo−B=apolipoprotein B; bOHBut=beta−hydroxybutyrate; Cit=citrate; Crea=creatinine; DHA=docosahexaenoic acid; FA=fatty acids; FAw3=omega−3; fatty acids; FAw6=omega−6 fatty acids; Glc=glucose; Gln=glutamine; Gly=glycine; Glyc−A=glycoprotein acetyls; HDL=high density lipoproteins; HDL−D=high density lipoprotein particle diameter; His=histidine; IDL=intermediate density lipoproteins; Ile=isoleucine; L=large; LA=linoleic acid; Lac=lactate; LDL=low density lipoproteins; LDL−D=low density lipoprotein particle diameter; Leu=leucine; LP=lipoprotein; M=medium; MUFA=monounsaturated fatty acids; PC=phosphatidylcholines; PG=phosphoglycerides; Phe=phenylalanine; PUFA=polyunsaturated fatty acids; Pyr=pyruvate; S=small; SFA=saturated fatty acids; SM=sphingomyelins; TotFA=total fatty acids; TotCho=total cholines; Tyr=tyrosine; Val=valine; VLDL=very low density lipoproteins; VLDL−D=very low density lipoprotein particle diameter; XL=very large; XS=very small; XXL=extremely large.

Supplementary Figure S16: Associations of genetically-predicted liability to type 2 diabetes with circulating metabolic biomarkers among participants aged 35-84 years at recruitment, by relatedness

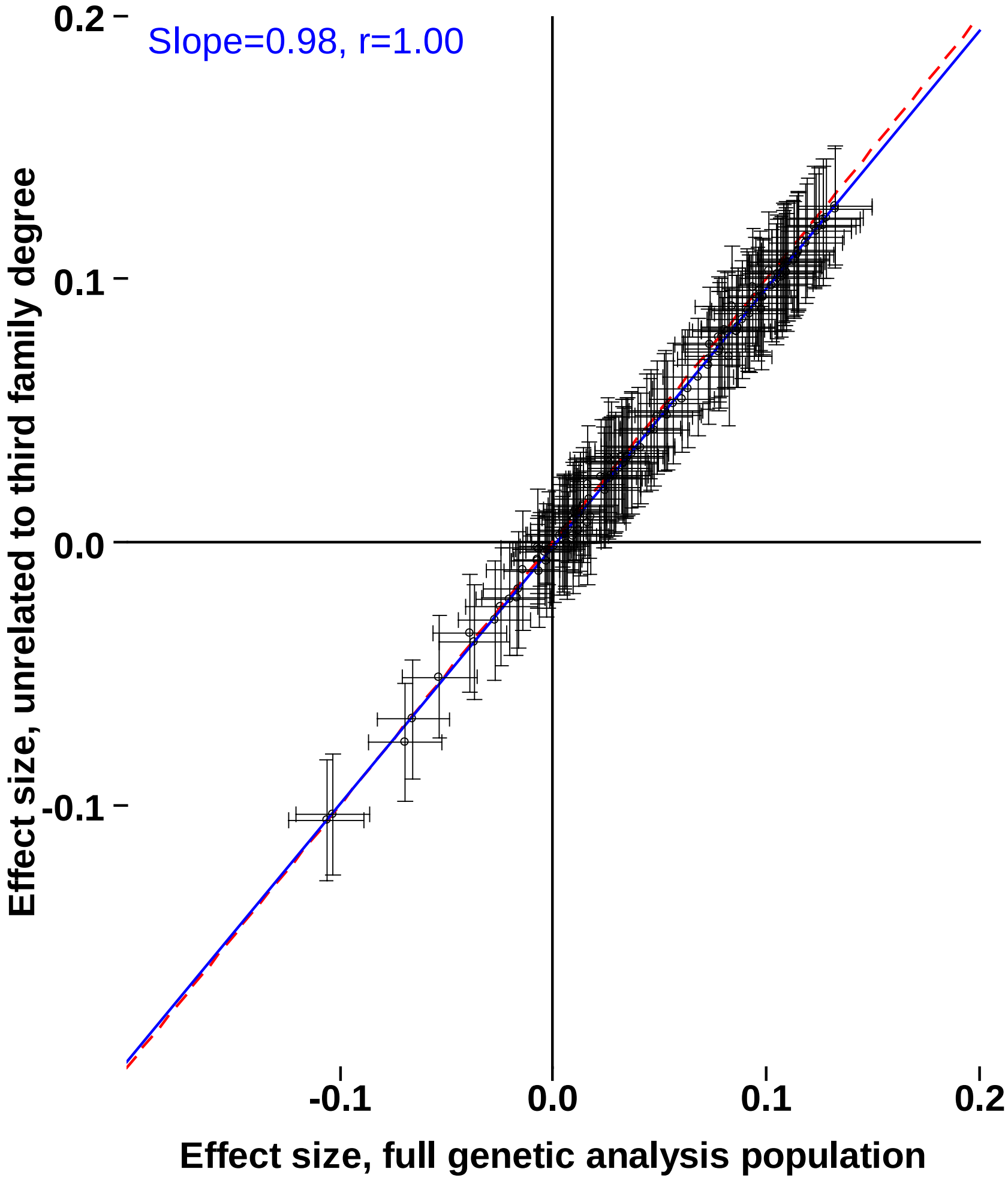

Difference (in SD units) of each log-NMR biomarker are per trebling in the genetically-predicted odds of type 2 diabetes and are adjusted for age, sex, the first 7 genetic principal components and fasting time. Red dashed line is line of equality. Blue line is line of best fit from a linear regression. Findings for the full genetic analysis population based on 125,587 participants, with the exception of 86,574 participants for beta-hydroxybutyrate, 121,391 participants for citrate, 121,368 participants for creatinine, 120,576 participants for glutamine, 118,255 participants for pyruvate, 121,374 participants for valine. Findings for population unrelated to the third family degree based on 71,432 participants, with the exception of 49,346 participants for beta-hydroxybutyrate, 68,991 participants for citrate, 69,009 participants for creatinine, 68,741 participants for glutamine, 67,449 participants for pyruvate, 68,985 participants for valine.

Supplementary Figure S17: Associations of pathway-specific type 2 diabetes GRSs with circulating metabolic biomarkers among participants aged 35-84 years at recruitment, by relatedness

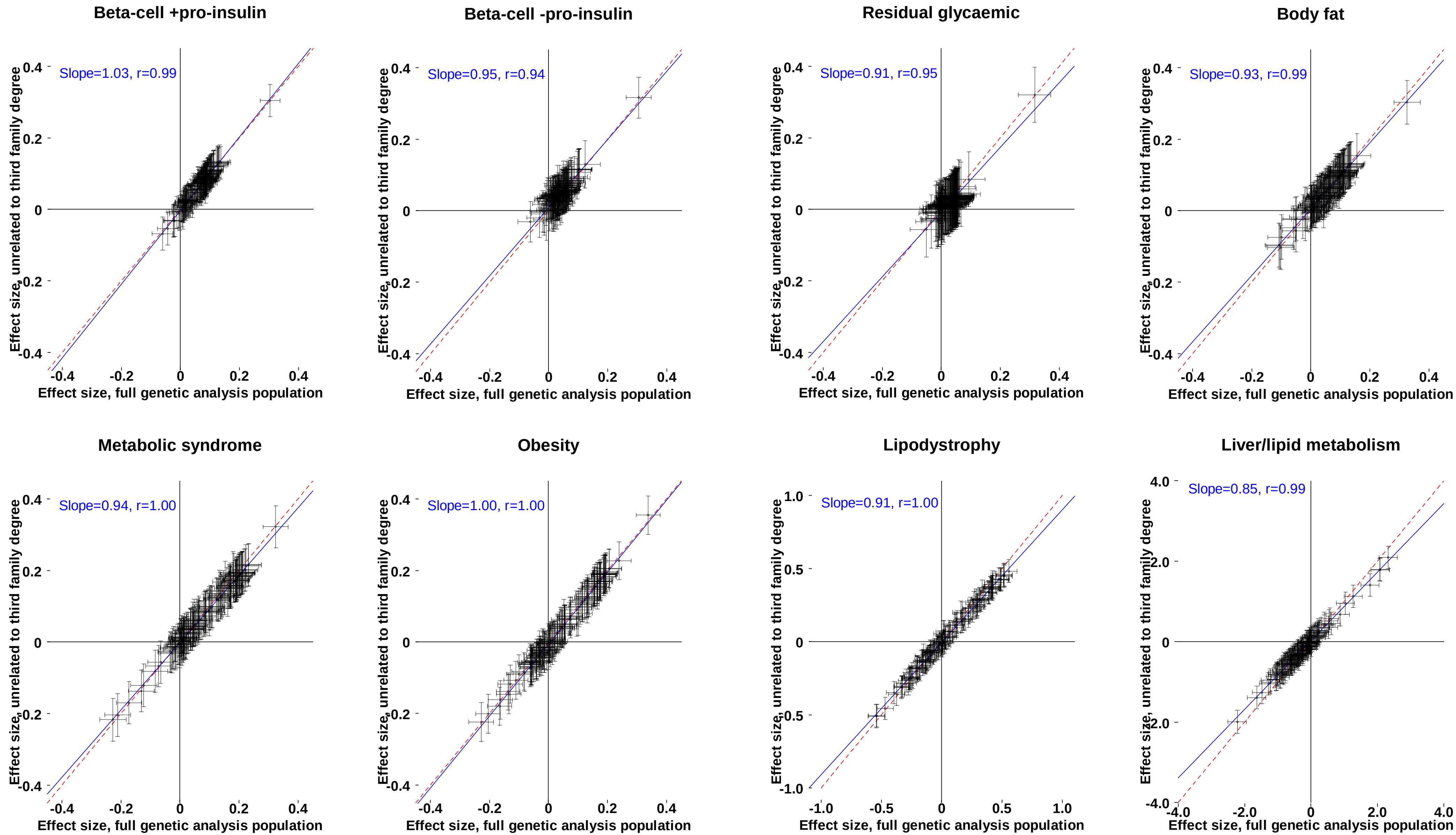

Difference (in SD units) of each log-NMR biomarker are per trebling in the genetically-predicted odds of type 2 diabetes and are adjusted for age, sex, the first 7 genetic principal components and fasting time. Red dashed line is line of equality. Blue line is line of best fit from a linear regression. Findings for the full genetic analysis population based on 125,587, with the exception of 86,574 participants for beta-hydroxybutyrate, 121,391 participants for citrate, 121,368 participants for creatinine, 120,576 participants for glutamine, 118,255 participants for pyruvate, 121,374 participants for valine. Findings for population unrelated to the third family degree based on 71,432 participants, with the exception of 49,346 participants for bOHBut, 68,991 participants for citrate, 69,009 participants for creatinine, 68,741 participants for glutamine, 67,449 participants for pyruvate, 68,985 participants for valine.

Supplementary Figure S18: Associations of genetically−predicted liability to type 2 diabetes with circulating metabolic biomarkers among 97,542 participants aged 35−84 years and without prediabetes at recruitment

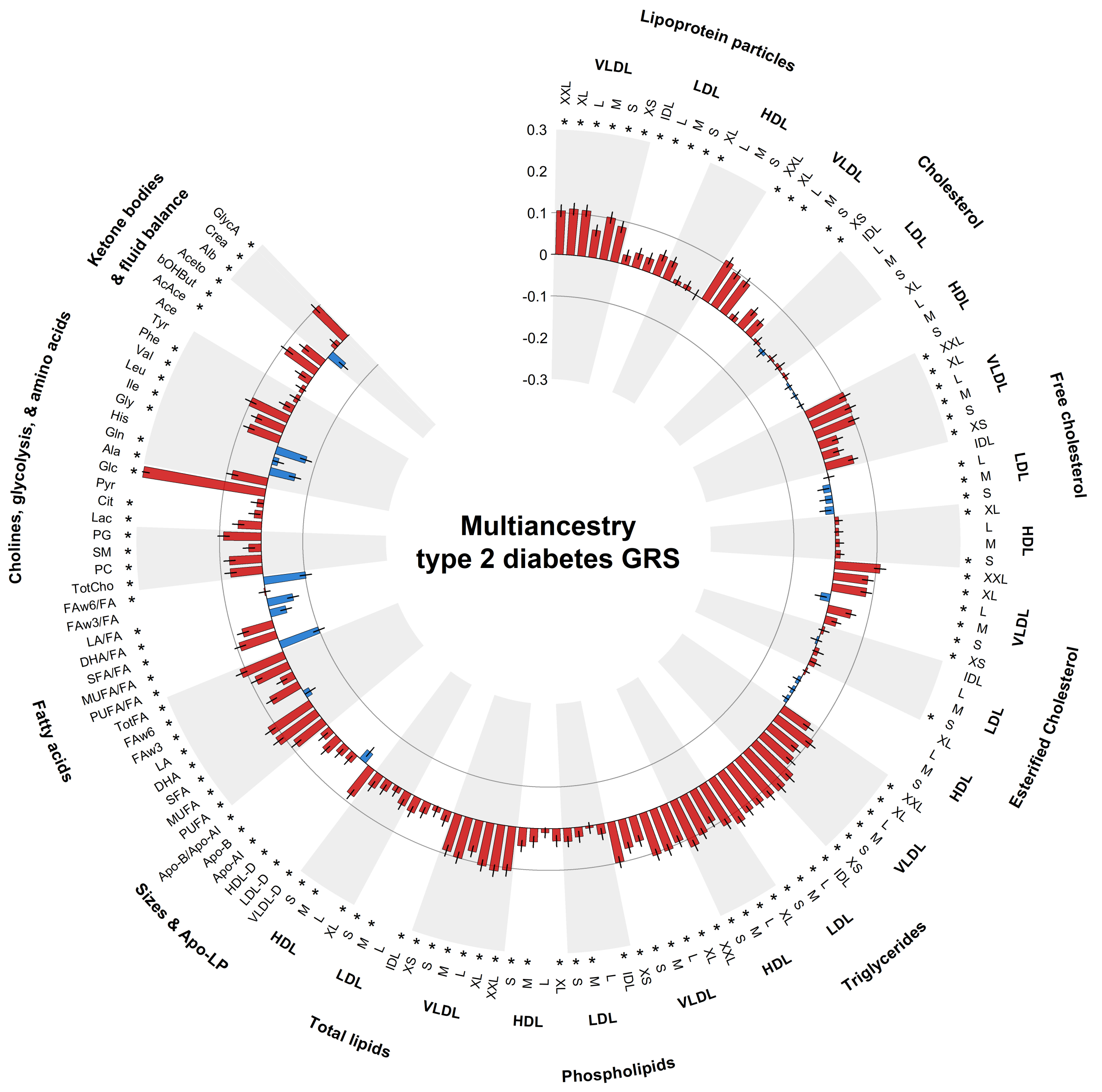

Difference (in SD units) of each log−NMR biomarker are per trebling in the genetically−predicted odds of type 2 diabetes and are adjusted for age, sex, the first 7 genetic principal components and fasting time. *False discovery rate controlled p<0.05. Findings based on 68,022 participants for bOHBut, 94,261 participants for citrate, 94,291 participants for creatinine, 93,724 participants for glutamine, 91,958 participants for pyruvate, 94,297 participants for valine. AcAce=acetoacetate; Ace=acetate; Aceto=acetone; Ala=alanine; Alb=albumin; Apo−A1=apolipoprotein A1; Apo−B=apolipoprotein B; bOHBut=beta−hydroxybutyrate; Cit=citrate; Crea=creatinine; DHA=docosahexaenoic acid; FA=fatty acids; FAw3=omega−3; fatty acids; FAw6=omega−6 fatty acids; Glc=glucose; Gln=glutamine; Gly=glycine; Glyc−A=glycoprotein acetyls; HDL=high density lipoproteins; HDL−D=high density lipoprotein particle diameter; His=histidine; IDL=intermediate density lipoproteins; Ile=isoleucine; L=large; LA=linoleic acid; Lac=lactate; LDL=low density lipoproteins; LDL−D=low density lipoprotein particle diameter; Leu=leucine; LP=lipoprotein; M=medium; MUFA=monounsaturated fatty acids; PC=phosphatidylcholines; PG=phosphoglycerides; Phe=phenylalanine; PUFA=polyunsaturated fatty acids; Pyr=pyruvate; S=small; SFA=saturated fatty acids; SM=sphingomyelins; TotFA=total fatty acids; TotCho=total cholines; Tyr=tyrosine; Val=valine; VLDL=very low density lipoproteins; VLDL−D=very low density lipoprotein particle diameter; XL=very large; XS=very small; XXL=extremely large. Prediabetes defined as HbA1c 5.7–6.4% among participants without previously diagnosed diabetes.

Supplementary text

Study design and participants

Details of the Mexico City Prospective Study (MCPS) design, its methods and population have been described previously (1). Briefly, between April 1998 and September 2004, 159,755 adults (52,644 men and 107,111 women) aged 35 years or older were recruited from households in two urban districts (Coyoacán and Iztapalapa) of Mexico City. Among 112,333 households with eligible residents, at least one individual was enrolled from 106,059 (94%) households. Ethics approval was obtained from the Mexican Ministry of Health, the Mexican National Council for Science and Technology and the University of Oxford, UK. All participants provided written informed consent.

Data collection

Trained nurses administered electronic questionnaires during household visits, collecting data on sociodemographic characteristics, lifestyle factors (eg, smoking, alcohol drinking and physical activity) and personal medical history (including current medication use). Measurements of height, weight, waist and hip circumferences and sitting blood pressure were undertaken using calibrated instruments with standard protocols. A 10 ml non-fasting venous blood sample was collected into an EDTA vacutainer, with time since last meal recorded. Following collection, blood samples were placed in portable insulated boxes with chilled packs, reliably maintaining a temperature of 4-10 °C, and transported on the day of collection to a central laboratory in Mexico City where samples were stored overnight in a refrigerator at 4 °C. Samples were centrifuged and separated into two plasma and one buffy coat aliquots the next morning (on average, approximately 24 hours after collection) and stored locally at −80 °C before being transported on dry ice to Oxford, UK for long-term storage at −150 °C. HbA1c levels were measured in buffy coat samples using a validated high-performance liquid chromatography method (2) on HA-8180 analysers with calibrators traceable to International Federation of Clinical Chemistry standards (3).

Assessment of diabetes status

Diabetes was ascertained through self-report of a prior doctor diagnosis of diabetes or current diabetes medication use (‘previously-diagnosed diabetes’), or an HbA1c

level ≥6.5% (48 mmol/mol) (4) among participants without previously-diagnosed diabetes ('undiagnosed diabetes'). Self-reported age at diagnosis and diabetes medication use were used to differentiate between likely type 1 diabetes (T1D) (diagnosed before 35 years and taking insulin at recruitment) and type 2 diabetes (T2D).

Metabolic biomarker quantification

Metabolomic profiling of baseline plasma samples was undertaken using a high-throughput targeted nuclear magnetic resonance metabolomics platform (5). This generated data on 168 directly measured biomarkers and 81 derived ratios of these, including quantification of lipids, fatty acids, amino acids, ketone bodies and other low-molecular-weight metabolic biomarkers and profiling of lipoprotein subclass distribution, particle size and composition. The present analyses include a subset of 143 of these metabolic biomarkers, prioritising directly measured biomarkers which could not be inferred from others. Metabolic biomarker levels were set to the lower limit of detection if they were below this level and were then log-transformed and standardised.

Genotyping and genetic instrument for T2D

Genotyping of study participants used the Illumina Global Screening array v2 (6). Following quality control (genotype and individual-level missingness, departure from Hardy-Weinberg equilibrium, and Mendel errors), genotyped data were imputed using the TOPMED imputation server, retaining variants with imputation information scores >0.4 (6). Per-individual proportions of Indigenous American, European, African and East Asian ancestry were estimated using ADMIXTURE software (6).

There were 1289 single nucleotide polymorphisms (SNPs) associated with T2D in the Type 2 Diabetes Global Genomics Initiative (T2D-GGI) meta-analysis of genome-wide association studies (7). Of these, 234 SNPs were ambiguous (n = 203) or unavailable (n = 31) in MCPS. The remaining 1055 SNPs were used to construct a genetic risk score (GRS) for genetically-predicted liability to T2D as the sum over these SNPs of their effect allele count multiplied by the log odds ratio (OR) for their effect on T2D in the T2D-GGI multi-ancestry meta-regression (7). Using an unsupervised hard clustering approach, the T2D-GGI identified eight non-overlapping subsets of T2D-associated SNPs based on their associations with

cardiometabolic traits (7), representing distinct biological pathways. Applying these to the 1055 SNPs included in the T2D GRS, 8 pathway-specific T2D GRSs were constructed: beta-cell dysfunction with a positive association with proinsulin (beta-cell +pro-insulin, 78 SNPs), beta-cell dysfunction with a negative association with proinsulin (beta-cell -pro-insulin, 72 SNPs), residual glycaemia (306 SNPs), body fat (220 SNPs), metabolic syndrome (137 SNPs), obesity (203 SNPs), lipodystrophy (37 SNPs), and liver and lipid metabolism (2 SNPs). Separate sensitivity analyses employed a Hispanic T2D GRS, constructed from the same 1055 SNPs but applying weights from Hispanic ancestry groups within the T2D-GGI (7).

Statistical analyses

All analyses excluded participants aged 85 years or older at recruitment, those with likely T1D, with missing metabolic biomarker data (with the exception of 3-hydroxybutyrate, citrate, creatinine, glutamine, pyruvate and valine, for which there were higher numbers of participants with missing data), missing or extreme exposure or covariate data, or those who reported taking lipid-lowering medication. Observational analyses additionally excluded participants with previously-diagnosed chronic diseases other than diabetes (ischaemic heart disease, stroke, chronic kidney disease, cirrhosis, cancer, emphysema). Genetic analyses excluded participants with missing genetic data or genetic data failing quality control checks.

In observational analyses, linear regression models were used to relate previously-diagnosed and undiagnosed T2D (separately and combined) to each metabolic biomarker. These models were adjusted for age (continuous), sex, district (Coyoacán and Iztapalapa), educational level (university or high school, middle school, elementary school, other), smoking status (never, former, occasional, <10 cigarettes per day, ≥10 cigarettes per day), alcohol drinking (never, former, current), height (four equal groups), weight (four equal groups), waist circumference (four equal groups), hip circumference (four equal groups) and fasting duration (four equal groups).

Genetic analyses were reported in accordance with Strengthening the Reporting of Observational Studies in Epidemiology using Mendelian Randomisation (STROBE-MR) guidelines (8). The study protocol was not pre-registered but analyses were planned prior to initiating the study. Logistic regression, with adjustment for age, sex

and the first seven genetic principal components (PCs), was used to assess per allele effects of each of the 1055 SNPs included in the T2D GRS on T2D in MCPS, subsequently comparing the associations with those reported in the T2D-GGI multi-ancestry meta-regression (7). Nagelkerke's pseudo R^2 (9) was used to assess the proportion of T2D variance explained on the liability scale by the T2D GRS, testing the Mendelian randomisation (MR) relevance assumption. Assessment of participant baseline characteristics across fifths of overall and pathway-specific T2D GRS distributions contributed to assessment of the MR independence assumption.

MR analyses estimating the associations of genetically-predicted T2D liability with circulating metabolic biomarkers were conducted using the ratio of coefficients method (10). Linear regression was used to assess associations of the T2D GRS with each metabolic biomarker, adjusting for age, sex, the first 7 genetic PCs and fasting duration. The derived coefficients were divided by the estimated T2D-GRS association from a logistic regression model relating the T2D GRS to observed T2D (combined previously-diagnosed and undiagnosed), adjusting for age, sex and the first 7 genetic PCs. To improve interpretability, the resulting causal estimates and their SEs were multiplied by 1.10 (i.e. ln 3), corresponding to a trebling in the genetically-predicted odds of T2D rather than a ~2.72-fold increase (11). (A trebling in odds may be thought of as an increase in risk from 10% [odds 1/9] to 25% [odds 1/3], or from 25% [odds 1/3] to 50% [odds 1].) Subgroup analyses compared causal effect estimates across strata of age, sex, district and Indigenous American ancestry proportion. Analyses were repeated using the 8 pathway-specific T2D GRSs. To further explore the extent to which associations of genetically-predicted liability to T2D with metabolic biomarkers were due to direct causal associations, where overall or pathway-specific T2D GRSs were found to be associated with potential confounders of the association, multivariable MR was performed, in which the T2D GRS and genetic instruments for potential confounders were included in a single model (12). Significance testing in observational and genetic analyses used the Benjamini-Hochberg method to control the false discovery rate at the 5% level (13).

Sensitivity analyses limited the study population to participants who were unrelated to the third family degree and, separately, to participants without prediabetes (defined as an HbA1c level of 5.7-6.4% [39-47 mmol/mol] (14) in participants without previously-diagnosed T2D). Further sensitivity analyses used a Hispanic T2D GRS

(7). The robustness of MR results to violations of the IV assumptions were explored using standard approaches based on summary data, specifically inverse-variance weighted, MR Pleiotropy Residual Sum and Outlier [MR-PRESSO] (15), weighted median (16), and MR-Egger methods (17), using the associations of individual SNPs with T2D in the T2D-GGI multi-ancestry meta-regression (7) and with metabolic biomarkers in MCPS.

References

1. Tapia-Conyer R, Kuri-Morales P, Alegre-Díaz J, et al. Cohort profile: the Mexico City Prospective Study. Int J Epidemiol 2006; **35**(2): 243-9.

2. Alegre-Díaz J, Herrington W, López-Cervantes M, et al. Diabetes and Cause-Specific Mortality in Mexico City. N Engl J Med 2016; **375**(20): 1961-71.

3. International Federation of Clinical Chemistry. International Federation of Clinical Chemistry (IFCC) Standardization of HbA1c. https://ngsp.org/docs/IFCCstd.pdf (accessed 24 April 2024).

4. International Expert Committee. International Expert Committee report on the role of the A1C assay in the diagnosis of diabetes. Diabetes Care 2009; **32**(7): 1327-34.

5. Soininen P, Kangas AJ, Würtz P, Suna T, Ala-Korpela M. Quantitative serum nuclear magnetic resonance metabolomics in cardiovascular epidemiology and genetics. Circ Cardiovasc Genet 2015; **8**(1): 192-206.

6. Ziyatdinov A, Torres J, Alegre-Díaz J, et al. Genotyping, sequencing and analysis of 140,000 adults from Mexico City. Nature 2023; **622**(7984): 784-93.

7. Suzuki K, Hatzikotoulas K, Southam L, et al. Genetic drivers of heterogeneity in type 2 diabetes pathophysiology. Nature 2024; **627**(8003): 347-57.

8. Skrivankova VW, Richmond RC, Woolf BAR, et al. Strengthening the Reporting of Observational Studies in Epidemiology Using Mendelian Randomization: The STROBE-MR Statement. JAMA 2021; **326**(16): 1614-21.

9. Nagelkerke NJD. A note on a general definition of the coefficient of determination. Biometrika 1991; **78**(3): 691-2.

10. Burgess S, Small DS, Thompson SG. A review of instrumental variable estimators for Mendelian randomization. Stat Methods Med Res 2017; **26**(5): 2333-55.

11. Bragg F, Kuri-Morales P, Trichia E, et al. Type 2 diabetes and cause-specific mortality in Mexico City: a Mendelian randomisation analysis. Lancet Reg Health Am 2025; **45**: 101082.

12. Burgess S, Thompson SG. Multivariable Mendelian randomization: the use of pleiotropic genetic variants to estimate causal effects. Am J Epidemiol 2015; **181**(4): 251-60.

13. Benjamini Y, Hochberg Y. Controlling the False Discovery Rate: A Practical and Powerful Approach to Multiple Testing. Journal of the Royal Statistical Society: Series B (Methodological) 1995; **57**(1): 289-300.

14. American Diabetes Association Professional Practice Committee. 2. Diagnosis and Classification of Diabetes: Standards of Care in Diabetes-2025. Diabetes Care 2025; **48**(1 Suppl 1): S27-s49.

15. Verbanck M, Chen CY, Neale B, Do R. Detection of widespread horizontal pleiotropy in causal relationships inferred from Mendelian randomization between complex traits and diseases. Nat Genet 2018; **50**(5): 693-8.

16. Bowden J, Davey Smith G, Haycock PC, Burgess S. Consistent Estimation in Mendelian Randomization with Some Invalid Instruments Using a Weighted Median Estimator. Genet Epidemiol 2016; **40**(4): 304-14.

17. Bowden J, Davey Smith G, Burgess S. Mendelian randomization with invalid instruments: effect estimation and bias detection through Egger regression. Int J Epidemiol 2015; **44**(2): 512-25.
